# Mitotic Stress Is an Integral Part of the Oncogene-Induced Senescence Program that Promotes Multinucleation and Cell Cycle Arrest

**DOI:** 10.1016/j.celrep.2015.07.055

**Published:** 2015-08-20

**Authors:** Dina Dikovskaya, John J. Cole, Susan M. Mason, Colin Nixon, Saadia A. Karim, Lynn McGarry, William Clark, Rachael N. Hewitt, Morgan A. Sammons, Jiajun Zhu, Dimitris Athineos, Joshua D.G. Leach, Francesco Marchesi, John van Tuyn, Stephen W. Tait, Claire Brock, Jennifer P. Morton, Hong Wu, Shelley L. Berger, Karen Blyth, Peter D. Adams

**Affiliations:** 1 Institute of Cancer Sciences, CR-UK Beatson Laboratories, University of Glasgow, Glasgow G61 1BD, UK; 2 Beatson Institute for Cancer Research, Glasgow G61 1BD, UK; 3 Fox Chase Cancer Center, Philadelphia, PA 19111-2497, USA; 4 Department of Cell and Developmental Biology, Perelman School of Medicine, University of Pennsylvania, Philadelphia, PA 19104-6058, USA; 5 School of Veterinary Medicine, College of Medical, Veterinary and Life Sciences, University of Glasgow, Glasgow G61 1BD, UK

## Abstract

Oncogene-induced senescence (OIS) is a tumor suppression mechanism that blocks cell proliferation in response to oncogenic signaling. OIS is frequently accompanied by multinucleation; however, the origin of this is unknown. Here, we show that multinucleate OIS cells originate mostly from failed mitosis. Prior to senescence, mutant H-RasV12 activation in primary human fibroblasts compromised mitosis, concordant with abnormal expression of mitotic genes functionally linked to the observed mitotic spindle and chromatin defects. Simultaneously, H-RasV12 activation enhanced survival of cells with damaged mitoses, culminating in extended mitotic arrest and aberrant exit from mitosis via mitotic slippage. ERK-dependent transcriptional upregulation of Mcl1 was, at least in part, responsible for enhanced survival and slippage of cells with mitotic defects. Importantly, mitotic slippage and oncogene signaling cooperatively induced senescence and key senescence effectors p21 and p16. In summary, activated Ras coordinately triggers mitotic disruption and enhanced cell survival to promote formation of multinucleate senescent cells.

## Highlights

• Multinucleate OIS cells originate from aberrant mitotic progression
• H-RasV12-expressing cells in mitosis show aberrant expression of mitotic genes
• H-RasV12-induced mitotic stress and increase in Mcl1 allow mitotic slippage
• Mitotic slippage and oncogene signaling cooperate to establish senescence

## Graphical Abstract

**Figure:**
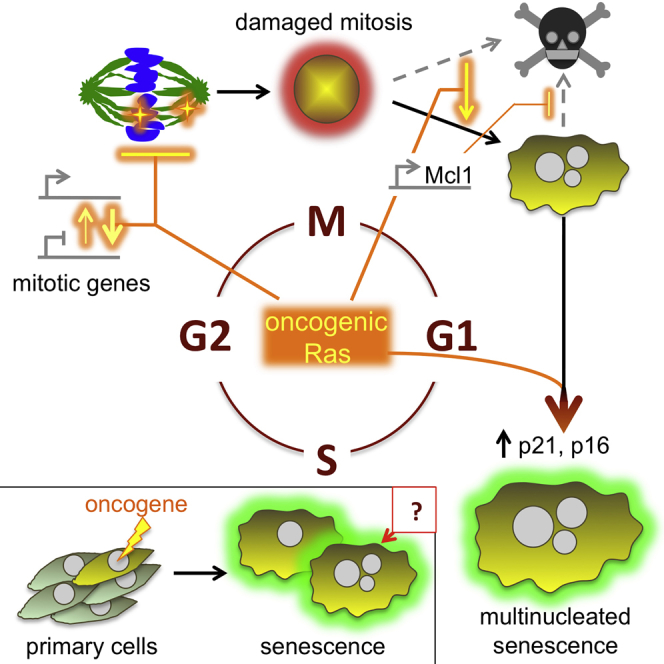

## Teaser

Dikovskaya et al. describe a mechanism of multinucleation during oncogene-induced senescence. The authors show that multinucleate senescent cells mostly originate from failed mitoses. They demonstrate that oncogene-induced mitotic defects, dysregulation of mitotic genes, and Mcl1-dependent apoptosis deficiency are the basis for multinucleation via mitotic slippage that further enhances senescence-associated cell-cycle arrest.

## Introduction

Cellular senescence is an important tumor suppressor mechanism and involves a stable proliferation arrest associated with an altered pro-inflammatory secretory pathway (Salama et al., 2014). In response to acquisition of an activated oncogene, primary human cells enter a proliferation-arrested senescent state called oncogene-induced senescence (OIS) (Braig et al., 2005; Chen et al., 2005; Collado et al., 2005; Michaloglou et al., 2005). Importantly, senescent cells, both in vitro and in vivo, frequently contain multiple nuclei in a single cell body (Salama et al., 2014). Indeed, appearance of multinucleated cells (MNCs) is a key feature of senescence (Vergel et al., 2010).

Pathways induced downstream of activated oncogenes include DNA replication stress and consequent DNA damage signaling. These effectors ultimately converge on the p16/pRB and p53/p21 tumor suppressor pathways (Salama et al., 2014). Senescence-associated proliferation arrest is generally thought to occur largely through a blockade to progression through G1 phase or early S phase (Campisi and d’Adda di Fagagna, 2007). Senescent cells can also be arrested in G2 (Mao et al., 2012), and more recent publications have documented the contribution of the premature activation of mitosis-specific E3-ligase, APC/C, to the onset of senescence (Johmura et al., 2014; Krenning et al., 2014). However, none of these mechanisms adequately explain the origin of multinucleate OIS cells.

Senescent cells within benign and/or early-stage neoplasia are at some risk of progression to malignancy if the senescence barrier is breached (Braig et al., 2005; Chen et al., 2005; Collado et al., 2005; Michaloglou et al., 2005). In this regard, human benign melanocytic nevi, neoplastic lesions of the skin composed largely of OIS melanocytes harboring activated *NRAS* or *BRAF* oncogenes (Gray-Schopfer et al., 2006; Michaloglou et al., 2005), frequently contain multinucleate melanocytes (Berlingeri-Ramos et al., 2010; Leopold and Richards, 1967; Patino et al., 2012; Savchenko, 1988). Multinucleate senescent melanocytes may harbor genome instability, a risk factor for malignancy (Fox and Duronio, 2013), and these cells have been proposed to give rise to highly proliferative, tumor-initiating stem-like cells (Leikam et al., 2015). Given that approximately 25% of melanomas are thought to arise in association with a pre-existing nevus (Smolle et al., 1999; Stolz et al., 1989) it is important to understand the origin of multinucleate, potentially pre-malignant, OIS cells.

Here, we show that activated RAS triggers two processes in pre-senescent primary cells, mitotic stress and upregulation of the anti-apoptotic protein Mcl1. These events together lead to extended mitotic arrest, ultimately followed by slippage out of mitosis to generate multinucleate proliferation-arrested senescent cells. We also present evidence that this process potentiates OIS, likely contributing to frequent multinucleation OIS cells observed in vivo.

## Results

### OIS Is Accompanied by Multinucleation

**Figure 1:**
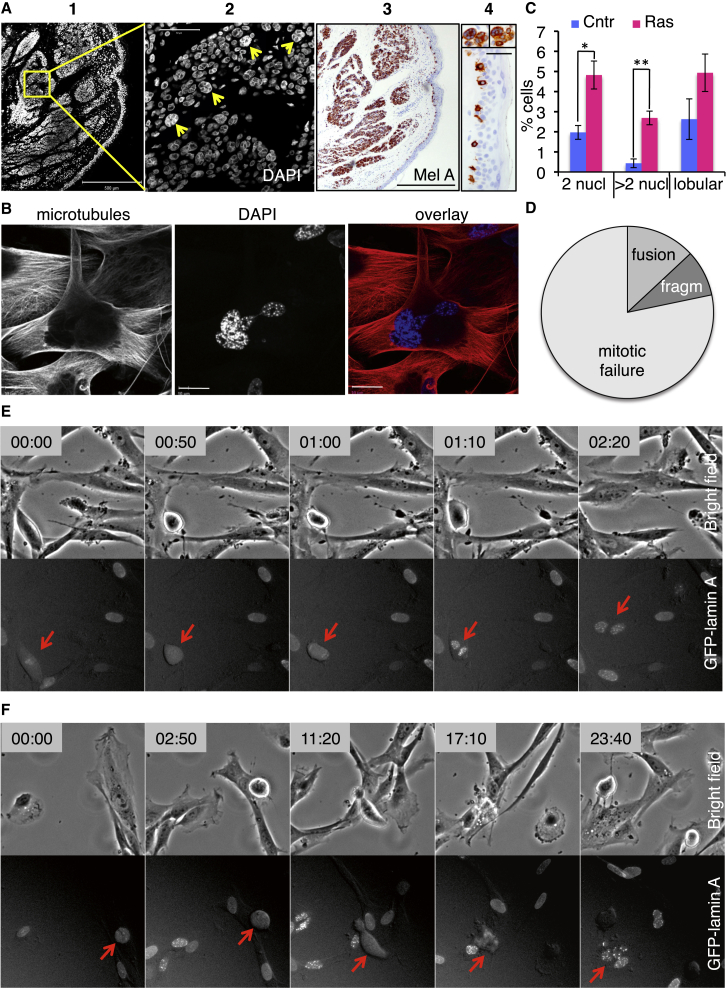
Multinucleated OIS Cells Originate from Aberrant Mitosis (A) MNCs in human nevi. Dermal nevus-containing section of human skin stained with DAPI (panels 1 and 2) or for melan A (Mel A; panels 3 and 4). Panel 2 is a magnified insert with multinucleated nevus cells (arrows). Panel 4 shows a magnification of melan-A-positive multinucleated nevus cells (top) and a section of overlaying epidermis with mononucleated melanocytes (bottom). Scale bars, 500 μm for panels 1 and 3, 50 μm for panel 2, and 100 μm for panel 4. 17% of nevus cells (out of 334) and 0% epidermis melanocytes (out of 365) are multinucleated in this specimen. (B) Multinucleated senescent 12-day-induced ERRAS cell, stained for microtubules (left, red on overlay) and DAPI (middle, blue on overlay). Scale bars, 30 μm. (C) 15-day ERRAS induction (Ras) increases the percentage of MNC with two nuclei, more than two nuclei, and lobular nuclei. Data indicate means ± SEM from three independently derived ERRAS cell populations. ^∗^p = 0.021; ^∗∗^p = 0.005 (paired Student’s t test). Cntr, control. (D) Origin of multinucleation in GFP-Lamin A-expressing ERRAS cells observed throughout 12 days of Ras induction and classified as originating from mitotic failure (light gray), cell fusion (medium gray), and interphase fragmentation (fragm, dark gray). n = 23 multinucleation events (see also Figure S1I). (E and F) In (E), cytokinesis failure leading to binucleation is shown. (F) Prolonged mitotic arrest followed by slippage, generating a highly multinucleated cell. For (E) and (F), bright-field image (top) and corresponding GFP fluorescence (bottom) are shown at selected times (in hours:minutes, or hh:mm). Arrows indicate cells undergoing multinucleation. See also Figure S1 and Movies S1, S2, S3, S4, S5, S6, and S7.

To confirm previous reports of multinucleate senescent melanocytes in benign human nevi, we stained nevi with DAPI to detect DNA. This clearly revealed melan-A-positive nevus cells with multiple nuclei, while an overlaying epidermis contained only mononucleate melanocytes (Figure 1A). To investigate the origin of multinucleation in OIS, we generated primary human fibroblasts (IMR90) expressing tamoxifen-activatable oncogenic H-RasV12 fused to the estrogen receptor (ER) ligand-binding domain (Reuter and Khavari, 2006) (Figure S1A), hereinafter referred to as ERRAS cells. In this model, H-Ras signaling is readily induced with tamoxifen (hereinafter referred to as activated Ras or induced ERRAS cells), while uninduced cells serve as a control. As reported previously (Barradas et al., 2009; Lin et al., 1998; Reuter and Khavari, 2006; Young et al., 2009), activation of oncogenic H-RasV12 induced downstream MEK signaling (Figure S1A) and, after a transient proliferation burst, led to a gradual decrease in DNA synthesis (Figure S1B). Within 2 weeks, cell growth ceased, and cells displayed characteristic markers of senescence, such as senescence-activated β-galactosidase (SA-β-gal) and senescence-associated heterochromatic foci (SAHF) (Figures S1C and S1D) as previously described (Dimri et al., 1995; Narita et al., 2003). This was accompanied by statistically significant 2.5- and 6.2-fold increases in proportion of MNCs with two and more than two nuclei, respectively (Figures 1B, 1C, and S1E). Thus, this in vitro system recapitulates the multinucleation phenotype observed in OIS in vivo.

### Multinucleate OIS Cells Arise from Failed Mitoses

To delineate the process of multinucleation in OIS, we constitutively expressed a fluorescent-tagged nuclear envelope protein, GFP-Lamin A, in ERRAS cells. As expected, GFP fluorescence localized to the nucleus and outlined the nuclear envelope (Figure S1F), similar to endogenous Lamin A, with some nuclear foci as previously described (Hübner et al., 2006). Importantly, there was no difference in nuclear morphology between cells expressing GFP and GFP-Lamin A (Figure S1G). Fluorescent Lamin A facilitated continuous tracking of individual nuclei over several days, and mitotic cells (i.e., cells in M-phase of the cell cycle) were clearly identifiable by dispersal of the GFP-Lamin A fluorescence on breakdown of the nuclear envelope, rounded cell morphology, and compaction of chromatin (Movie S1). Mitotic cells were easily distinguished from cells undergoing apoptosis, in which nuclear GFP-Lamin A persisted until cessation of all cellular blebbing (Figure S1H; Movie S2). Long-term (3–4 days) time-lapse imaging of ERRAS cells expressing GFP-Lamin A revealed that, upon Ras activation, the majority of MNCs originate from failed mitosis, although a small number of cell fusions and fragmentation of lobulated interphase nuclei were also detected (Figures 1D and S1I; Movies S3 and S4). We observed two types of multinucleation events linked to mitosis. One was a binucleation upon cytokinesis failure after a mitosis of a normal duration (Figure 1E; Movie S5). It was also observed at comparable frequency in control cells (Figure S1I) and so was not specific to OIS. A second type, seen only in induced ERRAS cells (Figure S1I), followed a prolonged mitotic arrest and produced highly multinucleated cells (Figure 1F; Movie S6). It was accompanied by vigorous cell movement and was morphologically recognized as mitotic slippage (Brito and Rieder, 2006), an exit from mitosis into G1 without cell division (Rieder and Maiato, 2004). While mitotic potential of the entire population declined with the duration of Ras activation (Figure S1J), the percentage of mitoses that produced MNCs via slippage greatly increased (Figure S1K). MNCs often survived for at least several days (Figures 6B and S1E; Movie S7), likely contributing to multinucleation of senescent cells. We conclude that, during progression toward OIS, viable MNCs arise predominantly from failed mitoses.

### H-RasV12 Activation in Primary Cells Causes Mitotic Defects and Disruption of the Mitotic Gene Expression Program

**Figure 2:**
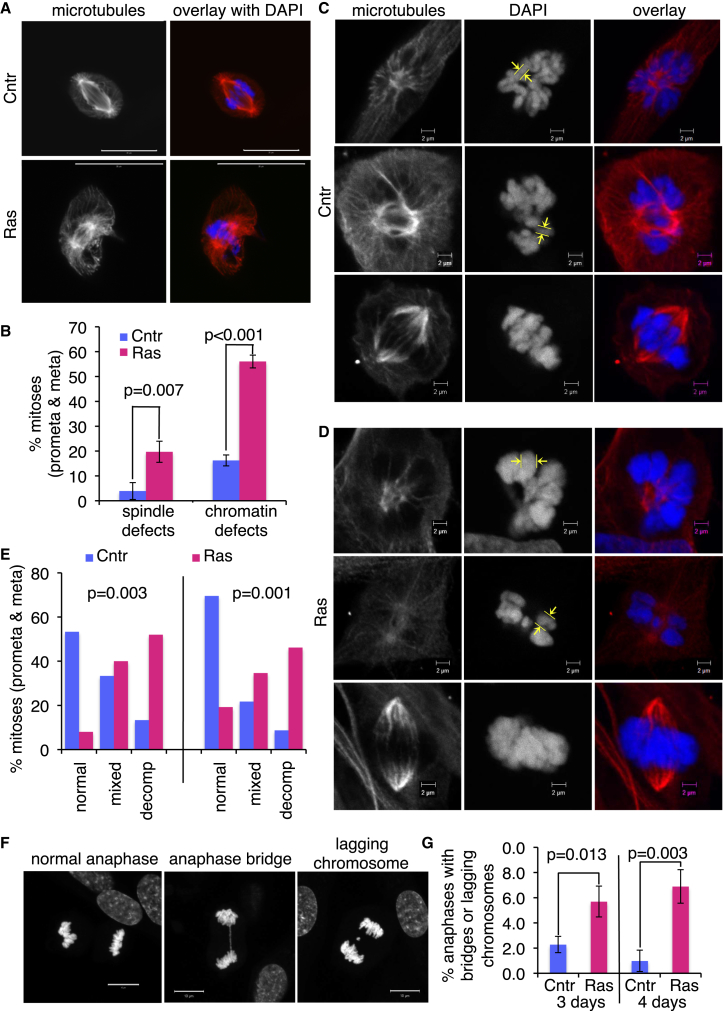
Mitotic Abnormalities in Pre-senescent Cells with Activated H-RasV12 (A) Mitotic spindles in control (top, Cntr) and induced (bottom, Ras) ERRAS cells, stained for microtubules (left panels, red in overlay) or DAPI (blue in overlay). Scale bars, 20 μm. (B) Quantification of chromatin and spindle defects (exemplified in A and D) in prometaphases (prometa) and metaphases (meta) of control (Cntr) or 5-day-induced (Ras) ERRAS cells. Data indicate means ± SD and p values from three replicates, 53–65 mitoses each. (C and D) Normal mitotic chromatin (C) or Ras-induced chromatin defect (D) in mitotic cells from prophase to metaphase. Single optical sections of 3D confocal images of control (Cntr) (C) or 3-day-induced ERRAS (D) cells stained for microtubules (left panels, red in overlay) or DAPI (middle panels, blue in overlay). Scale bars, 2 μm. Yellow frames show the visible width of chromosomes. (E) Quantification of chromatin decompaction in high-resolution 3D confocal images in two independent experiments. DAPI-stained prometaphases (prometa) and metaphases (meta) in control (Cntr) or 3-day-induced (Ras) ERRAS cells were scored for decompacted (decomp), partially decompacted (mixed), or normal chromatin (normal). Two independent experiments are shown. 15–26 mitoses per condition per experiment. p values for distribution changes calculated with two-way ANOVA. (F) Normal anaphase (left), aberrant anaphase with bridge (middle), or anaphase with a lagging chromosome (right). Scale bars, 10 μm. (G) Quantification of anaphase defects, shown in (F), in 3- and 4-day-induced (Ras) or control (Cntr) ERRAS cells. Data indicate means ± SD and p values from three replicas, 155–532 anaphases each.

Consistent with observed mitotic failure in induced ERRAS cells, we found a significant increase in spindle defects (Figures 2A and 2B) and abnormal chromatin morphology (Figures 2B–2E) in prometaphase and metaphase cells 3–5 days after Ras activation. Specifically, mitotic spindles were either misshapen or had low microtubule content (Figure 2A; data not shown), and prometaphase and metaphase chromatin appeared de-compacted (Figures 2C–2E; compare the diameters of individual chromosomes indicated in Figures 2C and 2D). Furthermore, the proportion of anaphases with either lagging chromosomes or anaphase bridges (Figure 2F) was significantly higher in cells after Ras activation (Figure 2G). Thus, H-RasV12 activation triggers mitotic abnormalities in primary human fibroblasts.

**Figure 3:**
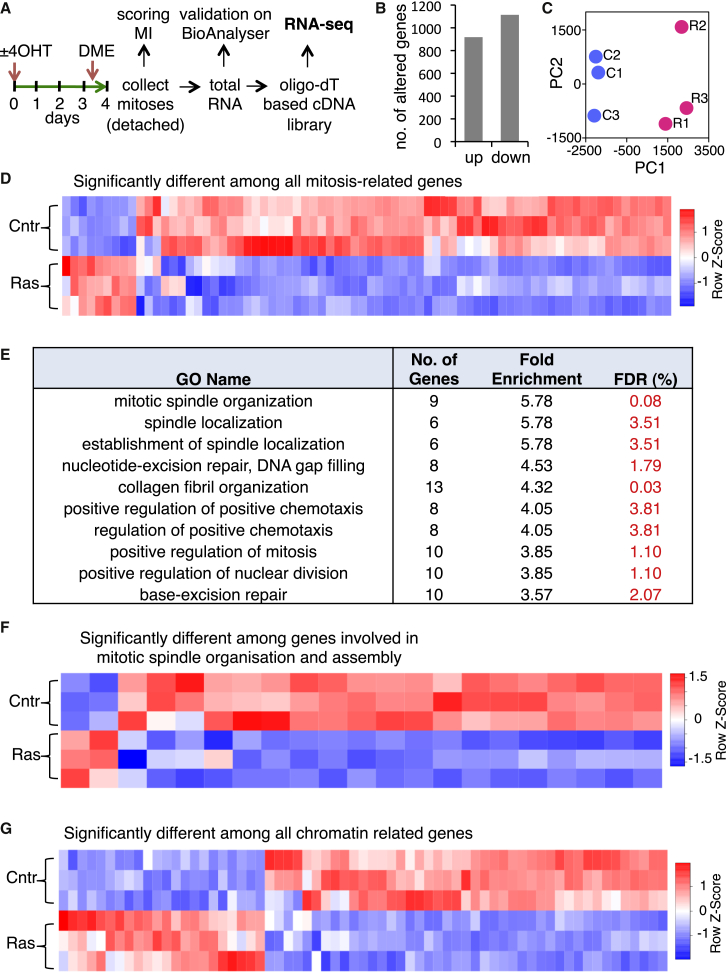
Activated H-RasV12 Dysregulates a Subset of Mitotic Genes in Pre-senescent Cells (A) Experimental layout. 4-day-induced (+4OHT) or control (−4OHT) ERRAS cells were arrested in mitosis with DME (12–16 hr). Mitotic (detached) cells were selectively collected by a washout, and >90% mitotic index (MI) was confirmed by microscopic scoring. Total RNA was isolated and validated on the Bioanalyzer. A cDNA library was constructed from poly(A) RNA and subjected to RNA-seq. The experiment was independently repeated three times. See Table S1 for alignment statistics. (B) Total number of significantly upregulated genes (up) and downregulated genes (down) genes (5% FDR) in Ras-induced mitotic cells compared to control mitotic cells. (C) PCA of Ras-induced (R1, R2, and R3) and control (C1, C2, and C3) mitoses based on expression of each known coding gene by FPKM. PC1 and PC2, principal components 1 and 2, respectively. (D) Heatmap of significantly differentially expressed genes (5% FDR) within the mitotic-related gene set (collated from several GSAE/MSigDB entries, Broad Institute) between Ras-induced mitotic cells and control mitotic cells. Genes are represented in columns, and samples are represented in rows. In the FPKM-based column Z-score, intensity represents higher (red) to lower (blue) expression. (E) DAVID GO analysis of differentially expressed genes between Ras-induced mitotic cells and control mitotic cells. The top ten most enriched ontologies are shown (FDR <5%). (F) Heatmap of gene expression for genes within mitotic-spindle-related GO terms, derived and represented as in (D). (G) Heatmap of gene expression for genes within the chromatin gene set derived and represented as in (D). See also Tables S1 and S2.

**Figure 4:**
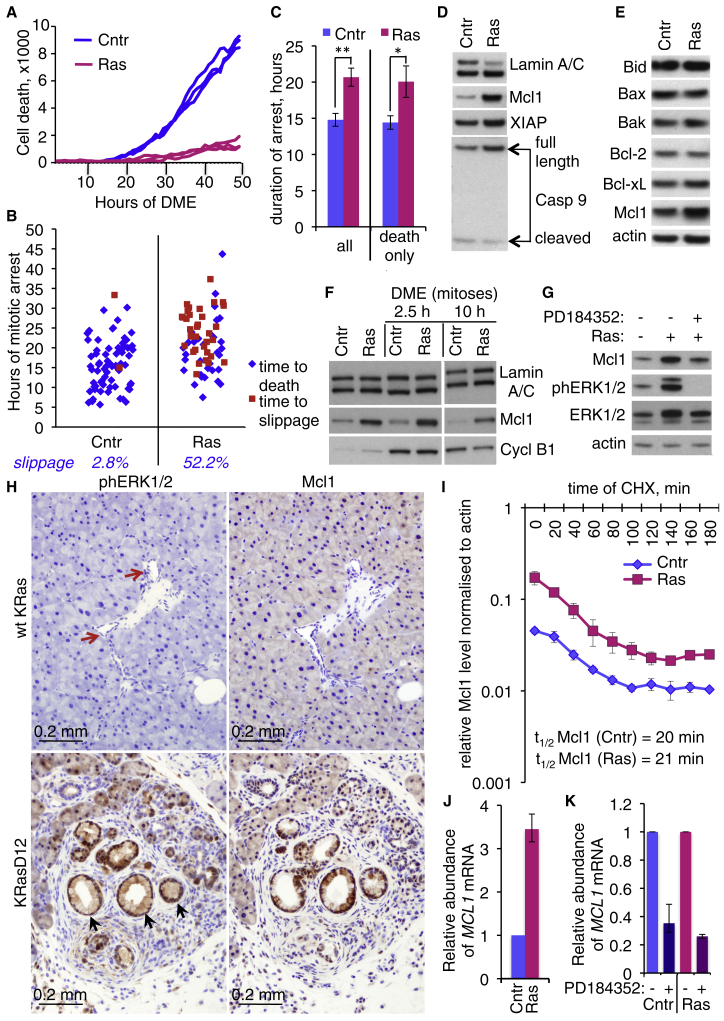
Activated H-RasV12 Confers Resistance to Mitotic Death and Upregulates Mcl1 (A) H-RasV12 protects from cell death caused by DME. Kinetics of cell death in DME-treated 4-day-induced (Ras, red) or control (Cntr, blue) ERRAS cells, measured by Sytox Green inclusion. The y axis shows numbers of dead cells recognized as fluorescent objects within four Incucyte images minus background. Triplicate measurements are shown. (B) Enhanced mitotic slippage in cells with activated Ras. Control (Cntr) or 4-day-induced (Ras) ERRAS cells were imaged for 3 days under DME, and duration of mitotic arrest prior to cell death (blue dots) or prior to slippage (red dots) was quantified in randomly selected 71 (Cntr) or 67 (Ras) mitoses. Percentage of slippage is shown below. The experiment was repeated at least five times with similar outcome. (C) Mean duration (±SEM) of DME-induced mitotic arrest in all cells (all, left) or leading to cell death (death only, right) in control (Cntr) or 3- to 5-day-induced (Ras) cells, quantified from four independent experiments. ^∗^p value = 0.025; ^∗∗^p value = 0.003 (paired Student's t test). (D and E) Mcl1 protein level is increased in 4 days induced ERRAS (Ras), compared to control cells (Cntr). Cell lysates were blotted with panel of antibodies against apoptotic regulators and Lamin A/C or actin as loading controls. (F) 4 days of induction increases Mcl1 in mitotically arrested ERRAS cells. Control (Cntr) or induced (Ras) ERRAS cells were treated with DME for 2.5 or 10 hr as indicated, collected by shake-off or left untreated (first two lanes), and immunoblotted for Mcl1 and Cyclin B1. Lamin A/C is a loading control. (G) Reduction in Mcl1 protein level in 7-day-induced ERRAS upon ERK inhibition by 4 hr of PD184352. Simultaneous reduction in ERK1/2 phosphorylation at Thr202/Tyr204 is shown. Actin is a loading control. (H) Colocalization of high-level Mcl1 and phospho-ERK in mouse pre-malignant mPanIN. Sections of pancreas from 4-month-old *PDX1*-*Cre* mice, wild-type (WT) for *KRas* (top) or heterozygous for *KRas*^*G12D*^ (bottom), with pancreatic ducts (top, red arrows) or mPanINs (bottom, black arrows) stained for phospho-Thr202/Tyr204 ERK1/2 (left) or Mcl1 (right). (I) Mcl1 protein stability is not altered by Ras induction. Relative amount of Mcl1 protein quantified from Mcl1/actin immunoblots from induced (Ras) or control (Cntr) ERRAS cells at indicated time in cyclohexamide (CHX). Data indicate mean ± SD from triplicate samples. The calculated half-lives of Mcl1 protein for both conditions are shown. (J) qPCR analysis of *MCL1* mRNA level in control (Cntr) and 4-day-induced (Ras) ERRAS cells. (K) Effect of 4 hr of PD184352 on *MCL1* mRNA level in control or 4-day-induced ERRAS cells measured by qPCR. (J and K) Mean (±SD) relative abundance (with control set up as 1) of mRNA from three technical replicas, normalized to *GAPDH*. See also Figures S2 and S3.

To identify potential cause(s) of mitotic defects during the establishment of OIS, we performed RNA sequencing (RNA-seq) gene expression profiling of cells captured in mitosis 4 days after Ras activation (Figure 3A). Our isolation procedure yielded >90% mitotic cells from both induced and control ERRAS cells. RNA-seq revealed that Ras induction significantly altered the abundance of approximately 2,000 gene transcripts in mitotic cells (Figure 3B). Principal-component analysis (PCA) showed that replicates were highly consistent (Figure 3C). Consistent with Ras-induced defects in mitosis, we found that, out of 371 genes included in mitosis-related Gene Ontology (GO) terms, 74 were significantly (5% false discovery rate [FDR]) altered in mitoses with activated Ras (Figure 3D; Table S2). This constituted a statistically significant (empirical p value < 0.0001) 2.17-fold enrichment of alterations in this gene set over randomly expected changes. Furthermore, out of 328 transcripts that were highly up- or downregulated in normal mitosis compared to the unsynchronized control ERRAS cell population [that are potentially relevant to mitotic processes (Cho et al., 2001)], 64 (approximately 20%) were significantly (5% FDR) altered by H-RasV12 in mitotic cells (2.11-fold increase over random, empirical p value < 0.0001). More importantly, mitotic spindle-related gene ontologies (namely, “mitotic spindle organization,” “spindle localization,” and “establishment of spindle localization”) were the top three most altered GO terms (Figures 3E and 3F; Table S2), consistent with the diverse spindle defects in induced ERRAS cells (Figures 2A and 2B). Changes in the spindle-related gene set were, significantly (empirical p < 0.0001), 3.18-fold enriched over randomly expected. In line with the observed chromatin defect in these cells (Figures 2C–2E), the expression of chromatin regulators was also significantly (5% FDR) changed (Figure 3G; Table S2) (1.26-fold enrichment, empirical p = 0.0318). Underscoring the specificity of these changes, the spindle checkpoint GO term was not significantly altered in Ras-induced mitotic cells (empirical p = 0.75), consistent with efficient mitotic arrest in these cells (see Figures 4B and 4C). Thus, H-RasV12 activation dysregulates expression of a specific subset of mitotic genes linked to the observed mitotic abnormalities in pre-senescent cells.

### Activated H-RasV12 Suppresses Death and Promotes Slippage out of Aberrant Mitosis to Generate MNCs

To examine the fate of defective mitoses in primary cells with activated Ras, we induced widespread mitotic defects with an Eg5 inhibitor, Dimethylenastron (DME), which prevents centrosome separation and formation of a bipolar spindle. In many cell types, this engages the spindle checkpoint to arrest cells in mitosis and eventually leads to cell death (Rath and Kozielski, 2012). DME induced efficient mitotic arrest with characteristic monopolar spindles in both control and induced ERRAS cells (Figure S2A), consistent with an intact spindle checkpoint (discussed earlier). However, cells with activated Ras were much less sensitive to the cytotoxic effect of DME (Figure 4A). Ras attenuated DME-induced caspase-3 activation among all cells (Figure S2B) and specifically in phospho-H3-positive mitotic cells (Figure S2C). Consistently, DME treatment conferred a selective advantage on pre-senescent induced ERRAS cells over parental IMR90 cells in mixed culture (Figure S2D). Live cell imaging of individual mitoses revealed that activated Ras extended mitotic arrest and delayed death in mitosis under DME (Figures 4B and 4C). In addition, Ras activation dramatically increased the proportion of cells that exited mitotic arrest via mitotic slippage (Figure 4B). Consistent with nuclear fragmentation during slippage (Zhu et al., 2014), induced ERRAS cells that survived DME treatment displayed widespread multinucleation (Figure S2E). We conclude that activated H-RasV12 confers resistance to apoptosis triggered by aberrant mitosis. Instead, such cells tend to slip out of mitosis to generate MNCs.

### H-RasV12 Protects from Mitotic Death via ERK-Dependent Increase in Mcl1

To find the cause of Ras-induced resistance to mitotic cell death, we assessed the accompanied changes in regulators of mitotic cell death and apoptosis in general. In ERRAS cells, one such protein, Mcl1, showed a marked and sustained upregulation upon Ras activation (Figures 4D, 4E, and S3A). A more modest rise in XIAP level was also detected (Figure 4D), as previously published (Liu et al., 2005). Expression of other apoptotic regulators—namely Bid, Bax, Bak, Bcl-2, and Bcl-xL—was not altered by activated Ras. Mcl1 also increased upon tamoxifen-induced activation of ER-RasV12 in BJ fibroblasts (Figures S3B and S3C) or upon retroviral transduction of IMR90 cells with constitutively active H-RasV12 (Figure S3D). Importantly, cells arrested in mitosis also displayed the Ras-mediated rise in the level of Mcl1 (Figure 4F).

Elevated Mcl1 required continuous H-RasV12 signaling, since tamoxifen withdrawal from induced ERRAS cells reduced Mcl1 protein level, concomitant with the decline in MEK1/2 phosphorylation (Figure S3E). Importantly, Mcl1 was also reduced by MEK inhibitor PD184352, which abolishes phosphorylation of extracellular signal-regulated kinases 1 and 2 (ERK1/2) downstream of activated Ras (Figures 4G and S3F). In comparison, inhibition of mammalian target of rapamycin (mTOR) (with Everolimus), Nuclear Factor κB (NF-κB) (with parthenolide), or reactive oxygen species (ROS) (with N-acetylcysteine) had little effect on Mcl1 protein (Figure S3F). Furthermore, the high level of Mcl1 frequently overlapped with strong phospho-ERK1/2 staining in an in vivo mouse model of OIS in the pancreas, specifically in *KRAS*^*G12D*^-triggered pancreatic intraepithelial neoplasias (mPanINs) that contain senescent-like cells (Caldwell et al., 2012; Collado and Serrano, 2010; Hingorani et al., 2003; Morton et al., 2010) (Figure 4H).

Since activated ERK is known to stabilize Mcl1 protein (Ding et al., 2008; Domina et al., 2004), we asked whether activated Ras increases Mcl1 protein stability. Surprisingly, we found no difference in the half-life of Mcl1 protein between induced and control ERRAS cells treated with the protein synthesis inhibitor cycloheximide (Figure 4I). On the other hand, the mRNA level of *MCL1* increased 3- to 4-fold upon Ras activation (Figures 4J and S3G) and was efficiently reduced by MEK inhibitor PD184352 (Figure 4K). Together, this suggests that oncogenic H-RasV12 upregulates Mcl1 in primary cells via ERK-dependent increase in mRNA abundance.

**Figure 5:**
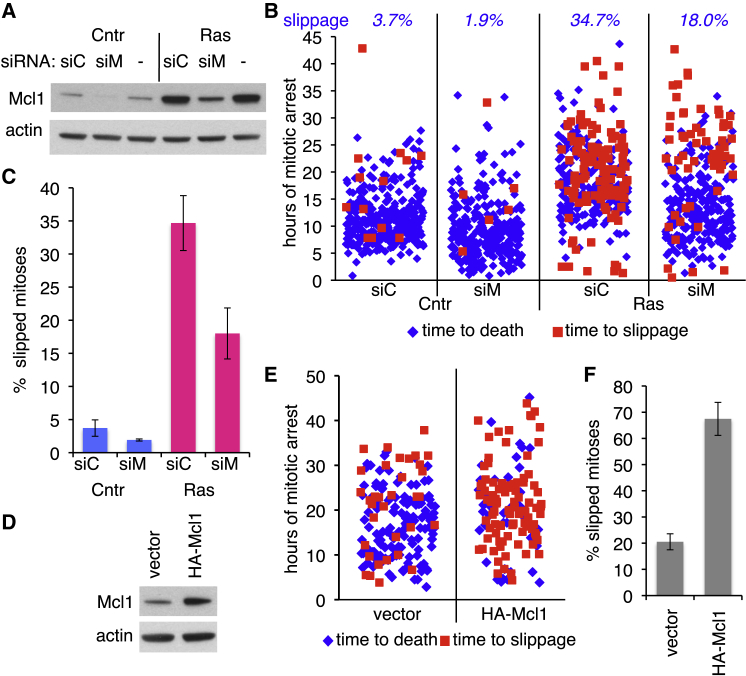
Increased Mcl1 Is Responsible for H-RasV12-Enhanced Mitotic Slippage (A) Mcl1 depletion by siRNA in control (Cntr) or induced (Ras) ERRAS cells. siC, non-targeting siRNA; siM, Mcl1-targeting siRNA; -, no transfection. Actin is a loading control. (B) Time-lapse analysis of duration and outcome of individual mitoses in control (Cntr) or 4-day-induced (Ras) ERRAS cells transfected with either Mcl1-targeting (siM) or non-targeting (siC) siRNA, treated with DME for 3 days. 207–337 mitoses per condition. Percentage of slippage is shown at top. (C) Percentage of mitotic slippage quantified from (B). Data indicate mean ± SEM from three biological replicates, 63–115 mitoses each. (D) Mcl1 level in ERRAS cells infected with retrovirus expressing HA-Mcl1 or vector only. Actin is a loading control. (E) Time-lapse analysis of duration and outcome of individual mitoses in DME-treated HA-Mcl1 or vector-expressing uninduced ERRAS cells, 141–165 mitoses per condition. (F) Percentage of mitotic slippage quantified from (E). Data indicate mean ± SEM from three replicates, 43–61 mitoses each.

To test the requirement for a high level of Mcl1 in Ras-induced resistance to mitotic death, we depleted Mcl1 from control and induced ERRAS cells using small interfering RNA (siRNA) (Figure 5A). Tracking individual mitoses in time-lapse images revealed that depletion of Mcl1 reduced slippage out of DME-induced mitotic arrest and increased mitotic cell death (Figures 5B and 5C). Furthermore, ectopic expression of Mcl1 (Figure 5D) in control cells increased slippage and reduced death in DME-treated cells (Figures 5E and 5F), recapitulating the effect of Ras activation. Thus, in ERRAS cells, Ras-mediated upregulation of Mcl1 is necessary and sufficient for enhanced survival and increased slippage of damaged mitoses, contributing to generation of MNCs by activated H-RasV12.

### H-RasV12-Expressing Slipped Cells Are Senescent

**Figure 6:**
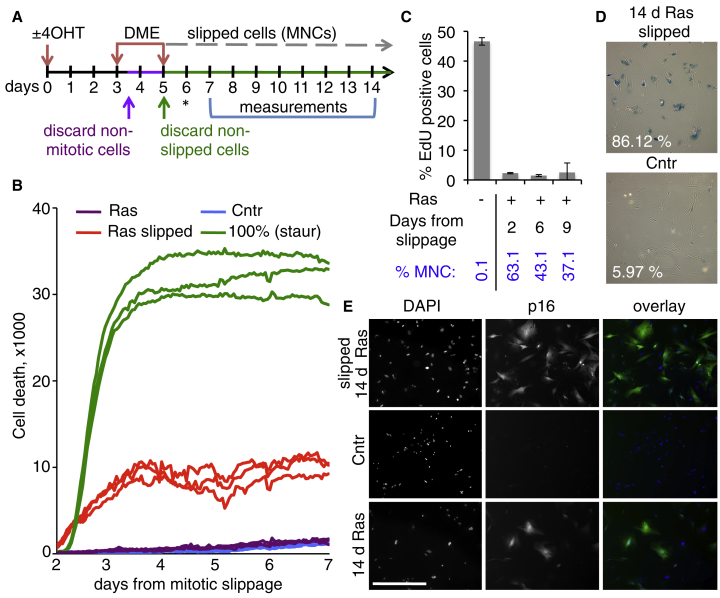
Survival and Senescence of Cells with Activated H-RasV12 following Mitotic Slippage (A) Experimental layout. Control (−4OHT) or 3-day-induced ERRAS cells (+4OHT) were arrested with DME for 9 hr (control cells were seeded in excess to account for lower level of mitotic slippage). Mitoses were collected and re-plated under DME for a further 40 hr. After washing away the remaining mitoses and cell debris, the slipped (attached) control and induced cells were re-seeded at the same cellular density (time corresponding to 5 days after induction). Time of siRNA transfection for Figures S3I and S3J is indicated by an asterisk. Measurements were performed during period indicated by bracket. (B) Cell death after slippage in induced ERRAS cells (Ras slipped), measured by incorporation of Sytox Green viability dye. Staurosporin treatment of control cells plated at the same initial density, 100% (staur), was used to visualize the maximal level of cell death in this assay (100% cell death was confirmed by visual examination). Untreated induced cells (Ras) as well as untreated control cells (Cntr) seeded at the same density are also shown. The data were acquired simultaneously with data in Figure S3J, which uses the Ras slipped and 100% (staur) data again as controls. Triplicate measurements are shown. (C) Slippage induces durable cell-cycle arrest in induced ERRAS cells. Percentage of replicating cells measured by EdU incorporation at indicated times after slippage (mean ± SD from seven replicates). Percentages of multinucleate cells in the samples are given below (% MNC). (D) SA-β-gal staining of 14-day-induced ERRAS cells 9 days after slippage (top), compared to untreated control ERRAS (bottom). Percentages of β-gal-positive cells are given. d, days. (E) p16 immunofluorescence in 14-day-induced ERRAS cells 9 days after slippage (top). p16 staining in control (middle row) and 14-day-induced (senescent, bottom) ERRAS cells are shown as negative and positive controls, respectively. Scale bar, 200 μm. Overlay colors: DAPI, blue; p16, green. See also Figure S3.

To determine the fate of the Ras-induced slipped MNCs, we incubated purified mitotically arrested cells with DME for an additional 40 hr and collected and reseeded cells that slipped (reattached) in this period, as shown in Figure 6A. Following this protocol, approximately two thirds of the induced post-slippage ERRAS cells survived for at least 9 days (Figures 6B–6E). They were multinucleated (Figures 6C and S2E); proliferation arrested as measured by 5-ethynyl-2′-deoxyuridine (EdU) incorporation (Figure 6C); SA-β-gal-, p21-, and p16 positive and cyclin B1 negative (Figures 6D–6E and S3H); and displayed large flat cell morphology (data not shown), all of which indicating that they are senescent. siRNA-mediated knockdown of Mcl1, abundant in these cells (Figures S3H and S3I), moderately promoted cell death (Figure S3J). Thus, a large proportion of Ras-expressing slipped cells becomes multinucleate senescent cells.

### Mitotic Slippage and Oncogene Activation Cooperate in Establishing Senescence

**Figure 7:**
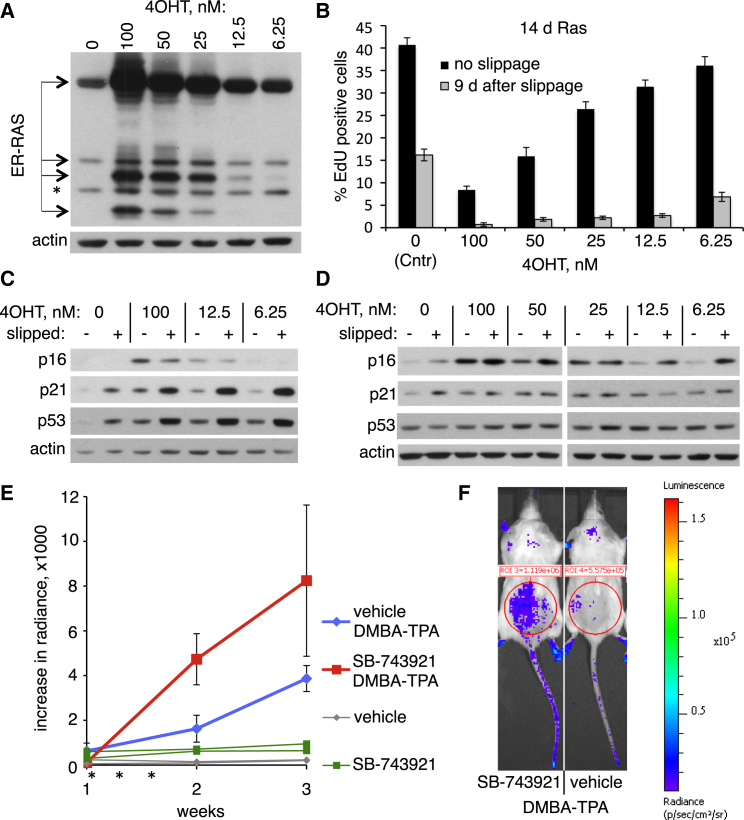
Mitotic Slippage and Low-Level Oncogene Signaling Cooperate to Upregulate p21 and p16 and Induce Senescence-Associated Cell Cycle Arrest (A) Titration of ER-H-RasV12 level in ERRAS cells by differential 4OHT concentration. Lysates from ERRAS cells induced for 7 days with indicated 4OHT concentrations (0–100 nM) were blotted with anti-Ras antibody that recognizes both endogenous Ras (asterisk) and ER-RasV12 fusion. Actin is a loading control. (B) Combination of mitotic slippage and low-level (subthreshold for OIS onset) H-RasV12 expression induces effective cell-cycle arrest in ERRAS cells. The experiment was conducted as in Figure 6A but using several concentrations of 4OHT. Percentage of replicating cells measured by EdU incorporation in control ERRAS cells (0, Cntr) or ERRAS cells treated with indicated concentrations of 4OHT are shown at 9 days after slippage (14 days of H-RasV12 induction). Data indicate means ± SD from seven replicate experiments. d, days. (C and D) Immunoblots of the lysates from samples shown in (B) collected at 2 (C) or 9 (D) days after slippage, corresponding to 7 (C) or 14 (D) days of Ras induction, to detect changes in protein amounts of p53, p21, and p16. Actin is a loading control. (E) Activation of p16-promoter-driven luciferase expression in p16^Luc/wt^ transgenic mice, treated with a combination of ectopic DMBA-TPA protocol and Eg5 inhibitor SB-743921. Mice received DMBA (week 0, not shown) followed by TPA during weeks 1–3. Three i.p. injections of SB-743921 or vehicle were given during the first week of TPA (shown as asterisks). Luciferase activity was measured weekly using the IVIS in vivo imaging system. Average increases in luciferase activity (as compared to week 0) ± SEM in vehicle-treated cohort (n = 8, blue) or in SB-743921-treated cohort (n = 9, red) are shown. Difference between cohorts is significant at week 2, with p < 0.05. Changes in luciferase expression in two individual vehicle-treated (gray) and two individual SB-743921-treated (green) mice without DMBA-TPA application are shown as controls. (F) Example of p16-promoter-driven luciferase expression activated by the DMBA-TPA protocol (third week) with (left) or without (right) SB-743921 injections in p16^Luc/wt^ mice, as measured by luminescence in the presence of D-licuferin substrate. The color key to radiance of luminescence is on the right. The measurement regions (cycles) corresponding to the areas subjected to DMBA/TPA applications are shown. See also Figures S4 and S5.

Next, we asked if mitotic slippage can promote establishment of OIS. To visualize any contribution of mitotic slippage to OIS-associated cell-cycle arrest, we decreased expression of oncogenic H-RasV12 in induced ERRAS cells by titrating down tamoxifen (Figure 7A). Resulting low levels of H-RasV12 were unable to induce cell-cycle arrest (Figure 7B, 12.5 nM and 6.25 nM tamoxifen, no slippage). Likewise, slippage alone (protocol as in Figure 6A, −4OHT), failed to induce stable proliferation arrest characteristic of senescence, and instead caused only transient cell-cycle arrest in cells that survived DME treatment (Figures 7B, S4A, and S4B). Enlarged nuclear volume and lobulated nuclei or micronucleation in the proliferating EdU-positive cells confirmed that these cells had likely slipped out of DME-induced mitotic arrest (Figure S4C). In addition, these slipped cells displayed only weak SA-β-gal staining (Figure S4D, left panel) and little p16 (Figure S4E, left panels), underscoring that they are not senescent. However, combined low-level H-RasV12 expression and DME-enforced slippage effectively induced long-term cell-cycle arrest (Figure 7B, 12.5 nM and 6.25 nM 4OHT + slippage; Figure S4A). Thus, mitotic slippage synergizes with low-level oncogenic Ras in establishing senescence-associated long-term cell-cycle arrest.

To gain insight into potential molecular drivers of such synergy, we examined the effect of DME-induced slippage and low-level oncogenic H-RasV12 on key effectors of cellular senescence. We found that 2 days after slippage (7 days of Ras induction), p53 and its target p21, but not p16, were cooperatively upregulated by low-level H-RasV12 and mitotic slippage (Figure 7C; proportions of S-phase cells at this stage are shown in Figure S4F). However, 9 days after slippage (Figure 7B, 14 days of Ras induction) expression of p16 was, instead, cooperatively upregulated by slippage and activated Ras (Figure 7D).

P16 is induced in mouse neoplasia, where it correlates with OIS (Burd et al., 2013; Kuilman et al., 2010). Using p16-reporter mice, heterozygous transgenic mice expressing a luciferase reporter gene under control of the p16 promoter (p16^luc/wt^) (Burd et al., 2013), we asked whether oncogene-induced p16 is altered by failed mitosis in vivo. Luminescence of p16 reporter was activated at the site of a 10-day-old wound (Figure S5A), consistent with upregulation of p16 during wound healing (Burd et al., 2013; Demaria et al., 2014; Jun and Lau, 2010). To induce senescence associated with an activated Ras oncogene, we subjected mice to a DMBA/TPA skin carcinogenesis protocol. This treatment is associated with *H-RasQ61L* mutation, formation of skin papillomas containing senescent cells, and, ultimately, progression to skin carcinomas (Fujiki et al., 1989; Quintanilla et al., 1986; Sun et al., 2007). The DMBA-TPA protocol by itself led to induction of the p16 promoter at the site of treatment (Figures 7E and S5B). Mitotic defects were induced by intraperitoneal (i.p.) injection of SB-743921, an Eg5 inhibitor with the highest efficiency and the lowest toxicity in vivo (Rath and Kozielski, 2012) that has undergone a phase 1 clinical trial in humans (Holen et al., 2011). On its own, SB-743921 induced mitotic aberrations but had no significant effect on p16-promoter-driven luciferase activity (Figures 7E and S5C). Remarkably, however, SB-743921 treatment together with DMBA-TPA induced a further significant increase in p16 activation (Figures 7E and 7F). This is consistent with our in vitro observation that mitotic disruption and an activated oncogene cooperate to upregulate p16.

## Discussion

Here, we report that frequently observed multinucleate OIS cells stem in large part from specific oncogene-induced mitotic abnormalities, in conjunction with oncogene-induced upregulation of Mcl1 that promotes survival and slippage out of aberrant mitoses. Furthermore, we show that progression through mitotic slippage can enhance senescence induced by weak oncogenic signaling.

Previously, multinucleated senescent cells were suggested to be generated via endomitosis/cytokinesis failure (Leikam et al., 2008; Takahashi et al., 2006), cell-cell fusion (Leikam et al., 2008), and “amitosis” (fragmentation of polyploid nuclei in interphase) (Walen, 2006). Combining long-term time-lapse imaging with fluorescent labeling of nuclei in cells undergoing OIS, we achieved a superior resolution that allowed an unbiased tracking of any detected multinucleated cells back to their origin. This unequivocally showed that multinucleation is predominantly due to mitotic failure. We further describe a spontaneous mitotic arrest and slippage in the course of OIS that generates senescent cells with multiple nuclei, a process not found in control primary cells.

Remarkably, we found that H-RasV12 expression in primary cells induces a range of mitotic defects that are well reflected in accompanying changes in gene expression, specifically in mitotic spindle and chromatin regulatory genes. We also established that activated Ras alters the fate of aberrant mitoses, from predominant cell death to frequent mitotic slippage. We determined that this depends on ERK-mediated increase in the level of anti-apoptotic protein Mcl1, in line with an established role of Mcl1 in mitosis (Harley et al., 2010; Topham and Taylor, 2013). Based on the aforementioned findings, we suggest that multinucleation in OIS results from oncogene-induced dysregulation of mitotic genes combined with Mcl1-dependent resistance to mitotic death, causing cells with excessive mitotic defects to exit mitosis via slippage, ultimately forming senescent MNCs. Although such multinucleation is a relatively rare event, genome aberrations associated with multinucleation can be a source of genome instability, which is, in turn, a contributor to malignancy (Fox and Duronio, 2013). Indeed, a recent study showed that multinucleate senescent melanocytes could give rise to tumor-initiating cells (Leikam et al., 2015). This is important, given that approximately 25% of melanomas are thought to arise in association with a pre-existing nevus (Smolle et al., 1999; Stolz et al., 1989)

Our data implicate aberrant mitosis in the establishment of OIS. MNCs are likely to be an extreme manifestation of the more widespread defects associated with mitotic aberrations (“mitotic stress”) during transition to senescence (compare Figures 1C and 2B). Aberrant mitotic progression was shown to generate de novo DNA damage (Colin et al., 2015; Ganem and Pellman, 2012; Hayashi and Karlseder, 2013). Considering that DNA damage response is a major driver of OIS (Di Micco et al., 2006), it is conceivable that damage associated with Ras-induced mitotic stress could also contribute to the establishment of senescence, in the same way that Ras-induced DNA replication stress triggers senescence (Di Micco et al., 2006). Indeed, we show that mitotic stress has the ability to potentiate pro-senescence oncogenic signaling. Our data suggest that p21 and, later, p16 are cooperatively upregulated by aberrant mitotic progression and sub-threshold H-RasV12, and, thus, it is possible that mitotic stress and oncogenic signaling cooperate to reach the critical level of senescence mediators.

In summary, our work has delineated a cellular process underlying multinucleation in OIS that involves impaired mitosis combined with increased Mcl1-dependent survival and subsequent slippage from aberrant mitosis, followed by a cell-cycle arrest. We propose that oncogene-induced mitotic stress cooperates acutely with other senescence effector pathways to induce OIS. However, in the long term, such multinucleate senescent cells may carry increased risk of malignant progression.

## Experimental Procedures

### Cells

Primary human fibroblasts IMR90 and BJ cells were retrovirally transduced with ER-H-RasV12-encoding pLNC-Ras:ER (Barradas et al., 2009) to generate ERRAS cells. ER-H-RasV12 was activated by continuous treatment with 100 ng/ml (unless indicated otherwise) 4-hydroxytamoxifen (4OHT). Hemagglutinin epitope tag (HA) containing pLZRS-HA-Mcl1, pLZRS control vector, GFP-fused Lamin A, or GFP alone was introduced to ERRAS cells or parental IMR90 cells via retroviral constructs. See the Supplemental Experimental Procedures for details. Cells were arrested in mitosis with 1 μM Eg5 inhibitor III (DME, Calbiochem/Merck, catalog number 324622) for indicated time and harvested by shake-off. For mitotic slippage, mitoses were further incubated with DME for up to 3 days, and any unattached cells and debris were washed away before attached (slipped) cells were harvested.

### siRNA

Mcl1 depletion was performed with siGENOME SMARTpool Mcl1-targeting siRNA, while non-targeting siRNA was used as a control (see details in the Supplemental Experimental Procedures).

### Cell Death/Apoptosis Assays

Apoptosis was detected by fluorescence of the NucView 488 caspase-3 substrate (Biotium) and quantified by flow cytometry (see details in the Supplemental Experimental Procedures). When required, it was combined with the identification of mitotic cells (by phospho-histone H3 staining). Alternatively, cells plated at identical densities were incubated in the presence of Sytox Green viability dye (Invitrogen #S7020), and kinetics of Sytox Green incorporation (reflecting cell death) was imaged, measured, and analyzed using the IncuCyte FLR imaging system (Essen Bioscience). Each data point represents the number of dead cells (automatically recognized as fluorescent objects) within four image acquisition windows after subtracting the background when necessary.

### Measurement of DNA Synthesis

BrdU incorporation after a 5-hr BrdU pulse was measured as described in the Supplemental Experimental Procedures. Alternatively, cells on 96-well plates were pulsed with EdU for 3 hr, followed by EdU detection using the Click-IT EdU imaging kit (Life Technologies). Plates were scanned with the Operetta High Content Imaging System (PerkinElmer) and analyzed as described in the Supplemental Experimental Procedures.

### Immunofluorescence

Cells cultured on glass coverslips were fixed by the appropriate method, immunostained as described in the Supplemental Experimental Procedures, and counterstained with DAPI. Image acquisition, processing, and analysis are detailed in the Supplemental Experimental Procedures.

### SA-β-gal Staining

Cells on coverslips were fixed with PBS containing 2% formaldehyde and 0.2% glutaraldehyde for 5 min, washed in PBS, and stained overnight at 37°C in staining solution containing 150 mM NaCl, 2 mM MgCl_2_, 5 mM K_3_Fe(CN)_6_, 5 mM K_4_Fe(CN)_6_, 40 mM Na_2_HPO_4_, and 1 mg/ml X-Gal (Sigma Aldrich #16664).

### Immunoblotting

Cellular lysates were prepared and analyzed on SDS-PAGE as described in the Supplemental Experimental Procedures. Protein stability was determined from the time-course measurements of Mcl1 and actin levels in immunoblots of cells treated with 10 μg/ml cycloheximide (Sigma) in triplicates, as described in the Supplemental Experimental Procedures.

### PCR and qPCR

Total RNA was extracted with the RNeasy Plus Mini Kit (QIAGEN), followed by DNase I treatment. cDNA was produced using oligo-dT primers. Semi-qPCR was performed with Mcl1 and APRT primers and visualized as detailed in the Supplemental Experimental Procedures. Real-time PCR was performed using separately designed *MCL1L*-specific primers (see Supplemental Experimental Procedures) with the SYBR-Green PCR Master Mix (Life Technologies) on the BioRad Chromo4 thermo cycler. Triplicate C(t) data for *MCL1* and a housekeeping gene (*GAPDH*) were analyzed using the REST program to calculate changes in gene expression.

### RNA-seq and Data Analysis

Total RNA isolated from mitotically arrested or unsynchronized cells was used to generate the cDNA library. Samples were sequenced on an Illumina NextSeq500. Paired-end reads were aligned to the human genome (hg19) using the splicing-aware aligner Tophat2. Reference splice junctions were provided by a reference transcriptome (Ensembl build 73). FPKM (fragments per kilobase million) values were calculated using Cufflinks. Differential gene expression was determined using the cuffdiff maximum likelihood estimate function. Genes of significantly changing expression were defined as FDR-corrected p value ≤ 0.05. See details in the Supplemental Experimental Procedures. RNA-seq data are available at GSE70668.

Histopathology, immunohistochemistry/tissue immunofluorescence, and details of animal studies are described in the Supplemental Experimental Procedures.

All animal work was carried out according to UK Home Office regulations, in line with the European Directive 2010 and approved by ethical review (University of Glasgow).

## Accession Numbers

The RNA-seq data reported in this paper have been deposited to the NCBI GEO and are available under accession number GEO: GSE70668.

## Supplemental Information

Document S1. Supplemental Experimental Procedures, Figures S1–S5, and Table S1

Table S2. RasV12-Induced Changes in Mitotic Transcripts, Related to Figure 3 This table lists genes (first column) involved in mitosis-related process (indicated as “Mitosis” in a second column, corresponding to heatmap shown in Figure 3D for significantly changed genes in H-RasV12-expressing mitoses), organization and assembly of mitotic spindle (indicated as “Mitotic spindle” in a second column, corresponding to Figure 3F for significantly changed genes in H-RasV12-expressing mitoses), or chromatin maintenance or function (indicated as “Chromatin” in a second column corresponding to Figure 3G for significantly changed genes in H-RasV12-expressing mitoses). Corresponding mean expression levels for uninduced (Control) and 4-day RasV12-activated (Ras) samples (from three replicas), log fold change, and false discovery rate (q value) are shown.

Movie S1. Mitosis, Related to Figure 1 Typical example of normal mitosis (indicated by arrow) in a GFP-Lamin A-expressing, 2-day-induced ERRAS cell. Bright-field image (top) and corresponding GFP fluorescence (bottom) are shown in parallel. Time (in hours:minutes, or hh:mm) is indicated at the top. Note the dispersal of nuclear-envelope-associated GFP signal as cell enters mitosis (at the 01:00 time point).

Movie S2. Cell Death, Related to Figures 1 and S1 Typical example of death in a GFP-Lamin A-expressing ERRAS cell. Bright-field images (left) and a corresponding GFP fluorescence (right) with cell of interest indicated by an arrow are shown in parallel. Time (in hours:minutes, or hh:mm) is indicated at the top. Note that nuclear-envelope-associated fluorescence persists until after nuclear and cytoplasm compaction and cellular immobilization (from the 00:50 time point onward) and is only lost simultaneously with the last (terminal) bleb (at the 04:10 time point).

Movie S3. Cell Fusion, Related to Figure 1 Typical example of cell fusion in induced GFP-Lamin A-expressing ERRAS cell. Bright-field images (left) and a corresponding GFP fluorescence (right) of 9-day-induced ERRAS cells are shown in parallel. Time (in hours:minutes, or hh:mm) is indicated at the top. Note that the two separate cells (indicated by arrows on GFP fluorescence images) fuse at the 05:50 time point, forming one binucleate cell that spreads (last frame) with two nuclei in close proximity.

Movie S4. Nuclear Fragmentation in Interphase, Related to Figure 1 An example of separation of lobulated nucleus in GFP-Lamin A-expressing, 2-day-induced ERRAS cell to two nuclei during interphase. Bright-field images (left) and a corresponding GFP fluorescence (right) are shown in parallel. Time (in hours:minutes, or hh:mm) is indicated at the top. Note that the nucleus of one of the cells (indicated by arrows in GFP images, a daughter cell generated from mitosis at 02:10) acquires 8-shaped form (at the 21:20 time point) and eventually separates into two (possibly connected) nuclei within one cell (last frame, right top corner), without intermittent loss of nuclear envelope fluorescence.

Movie S5. Binucleation, Related to Figure 1 Typical example of binucleation resulting from cytokinesis failure in induced GFP-Lamin A-expressing ERRAS cell. Bright-field images (left) and corresponding GFP fluorescence (right) are shown in parallel. Time (in hours:minutes, or hh:mm) is indicated at the top. Note the cell (indicated by arrow in the GFP time lapse) that enters mitosis at 1:30 and forms two nuclei without cell division at 02:30. Cell spreads at 02:50 as binuclear and remains as such until the end of the time lapse. While furrowing is not observed in this time-lapse sequence, we cannot exclude a transient furrow formed in the time between image acquisitions.

Movie S6. Multinucleation, Related to Figure 1 Typical example of multinucleation after prolonged mitotic arrest and slippage in induced GFP-Lamin A-expressing ERRAS cell. Bright-field images (right) and a corresponding GFP fluorescence (left) are shown in parallel. Time (in hours:minutes, or hh:mm) is indicated at the top. Note the cell (indicated by arrow in the GFP time lapse) that enters mitosis at the 01:10 time point and remains rounded for many hours until elongation (from approximately 09:30), constriction of the cell body in several places (at 13:00), and its apparent fragmentation (from 15:30). Nuclear envelopes start to reform in several parts of the cell (from 17:30) and the cell spreads as multinucleate (visible from 22:40).

Movie S7. Survival of Multinucleated Cell, Related to Figure 1 An example of Ras-induced GFP-Lamin A-expressing cells that remain viable for a long time after multinucleation via mitotic slippage. Bright-field images (top) and a corresponding GFP fluorescence (bottom) are shown in parallel. Note the cell that undergoes mitotic slippage at 02:40 and remains alive, motile, and multinucleated until the end of the time lapse (indicated by arrow in the GFP-fluorescent images). Time (in hours:minutes, or hh:mm) is indicated at the top.

Document S2. Article plus Supplemental Information
