# Supplementary material for: Mitotic Stress Is an Integral Part of the Oncogene-Induced Senescence Program that Promotes Multinucleation and Cell Cycle Arrest
Source: Cell Rep. 2015 Aug 20;12(9):1483–96. doi: 10.1016/j.celrep.2015.07.055 (PMC4562906; doi:10.1016/j.celrep.2015.07.055)
Supplement: Document S1. Supplemental Experimental Procedures, Figures S1–S5, and Table S1 [file mmc1.pdf]

Cell Reports
Supplemental Information

Mitotic Stress Is an Integral Part of the Oncogene-Induced Senescence Program that Promotes Multinucleation and Cell Cycle Arrest

Dina Dikovskaya, John J. Cole, Susan M. Mason, Colin Nixon, Saadia A. Karim, Lynn McGarry, William Clark, Rachael N. Hewitt, Morgan A. Sammons, Jiajun Zhu, Dimitris Athineos, Joshua D.G. Leach, Francesco Marchesi, John van Tuyn, Stephen W. Tait, Claire Brock, Jennifer P. Morton, Hong Wu, Shelley L. Berger, Karen Blyth, and Peter D. Adams

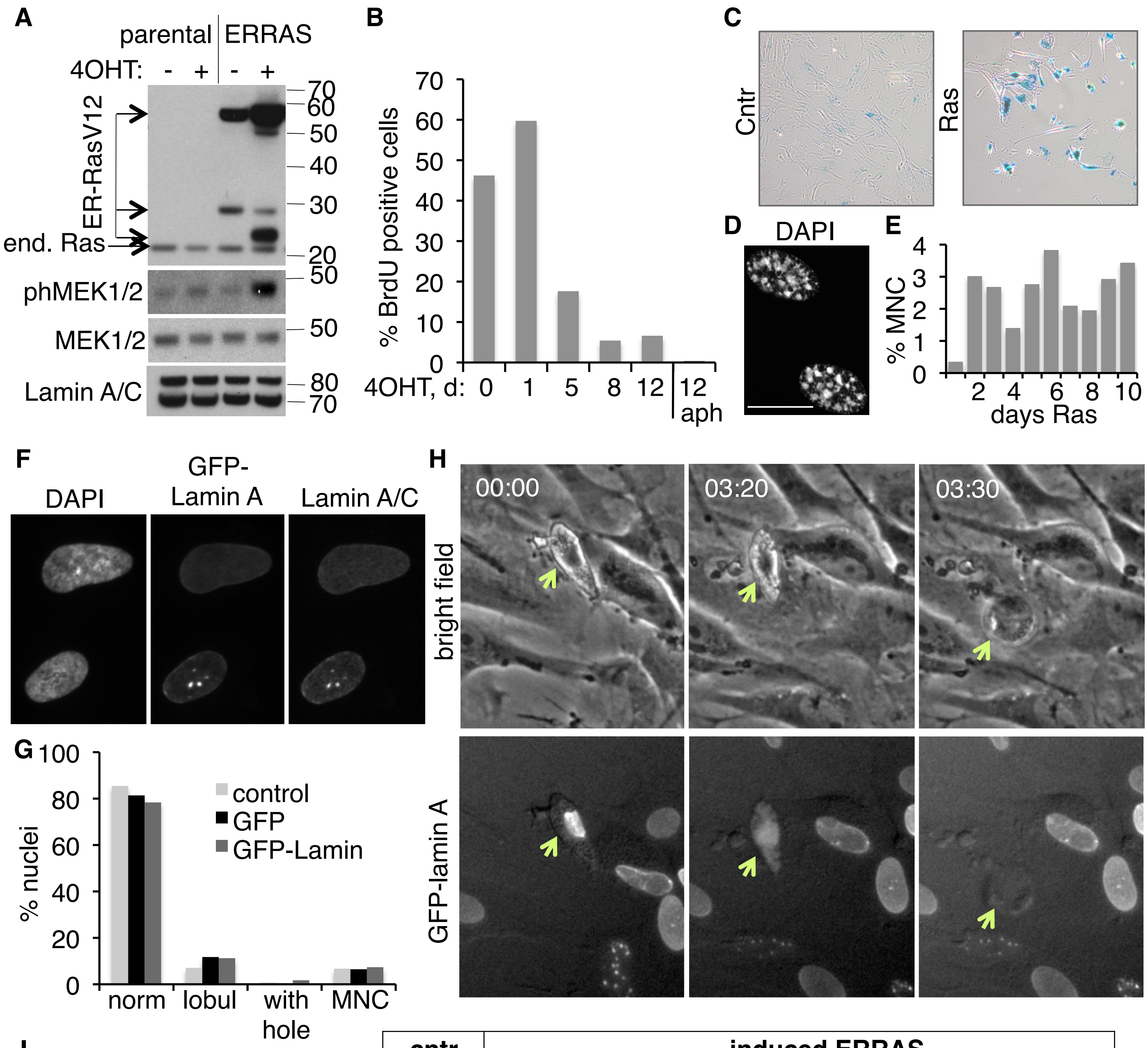

I

	cntr ERRAS	induced ERRAS				
		0-2 days	2-4 days	4-7 days	9-13 days	total (ind)
**cell fusion**	0	0	2	0	1	**3**
**slippage from mitotic arrest**	0	2	4	5	0	**11**
**cytokinesis failure**	6	2	1	3	1	**7**
**fragmentation of nucleus**	1	0	2	0	0	**2**
**total multinucleations**	**7**	**4**	**9**	**8**	**2**	**23**
**time-lapses tracked**	16	12	20	15	6	53
**number of cell divisions per time-lapse***	49.3 ±12.2	85.3 ±25.6	41 ±23.2	20.7 ±11.4	0.3 ±0.8	

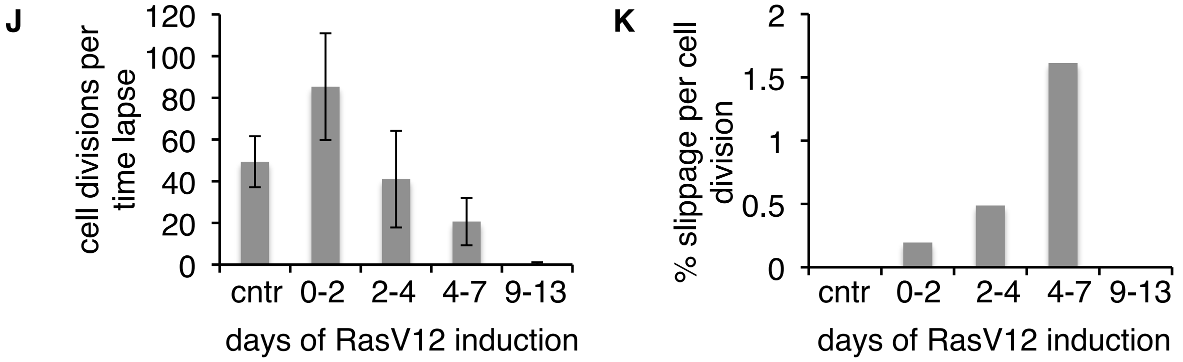

Figure S1

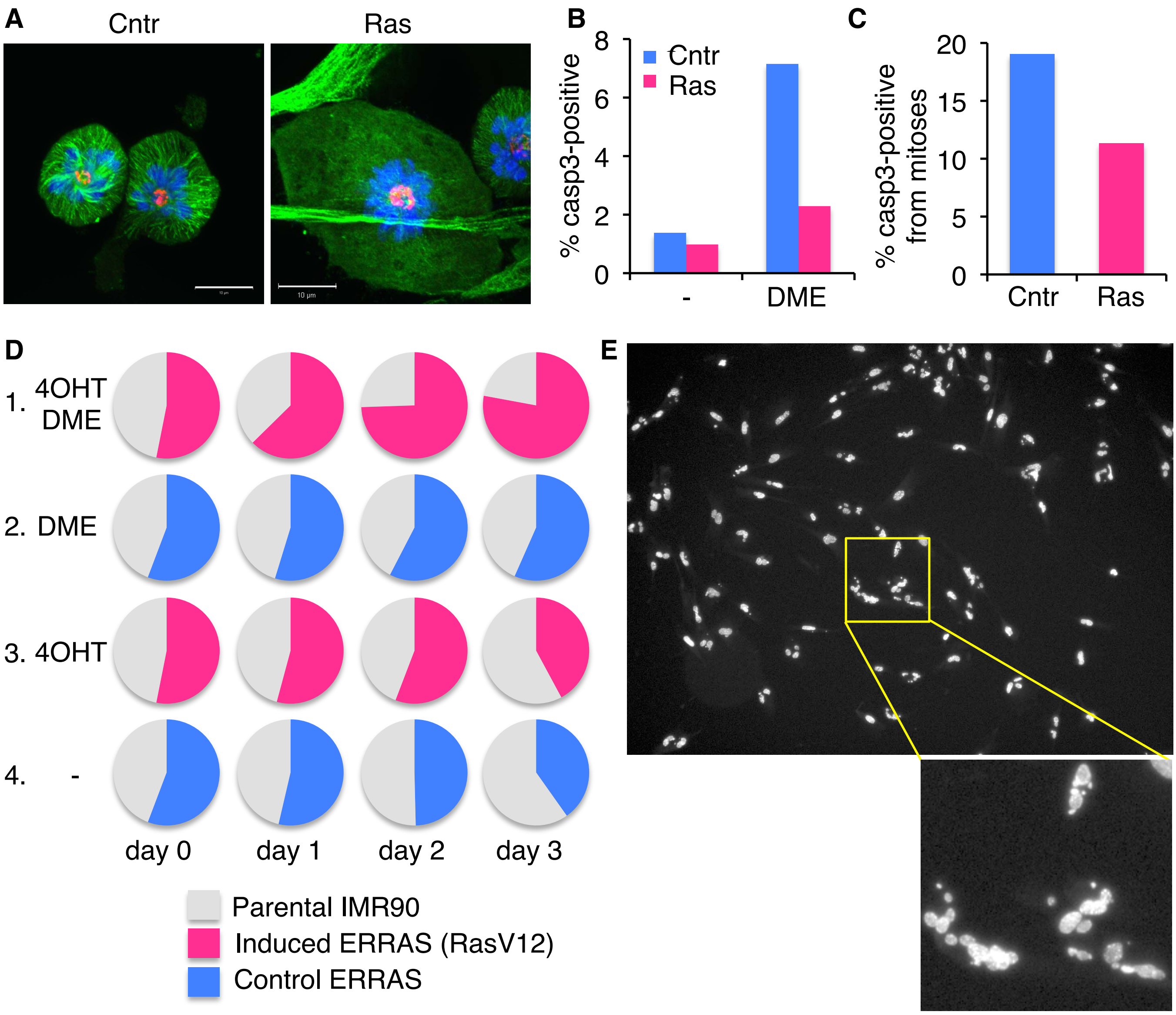
A
Cntr
Ras
10 µm
B
8
6
4
2
0
% casp3-positive
Cntr
Ras
-
DME
C
20
15
10
5
0
% casp3-positive from mitoses
Cntr
Ras
D
1. 4OHT DME
2. DME
3. 4OHT
4. -
day 0
day 1
day 2
day 3
Parental IMR90
Induced ERRAS (RasV12)
Control ERRAS
E

Figure S2

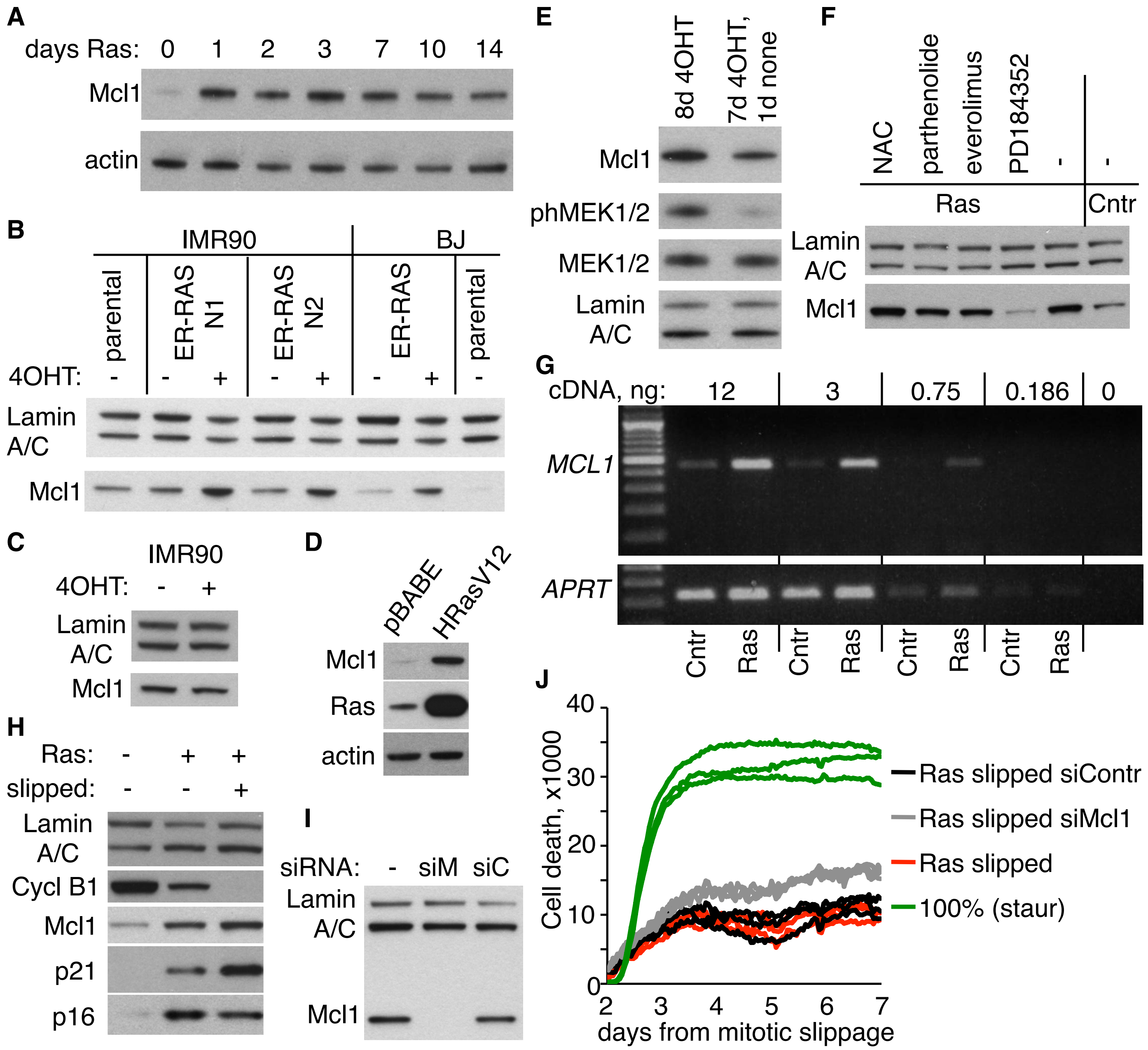
A
days Ras: 0 1 2 3 7 10 14
Mcl1
actin
B
IMR90
BJ
parental
ER-RAS N1
ER-RAS N2
ER-RAS
parental
4OHT: - - + - + - + -
Lamin A/C
Mcl1
C
IMR90
4OHT: - +
Lamin A/C
Mcl1
D
pBABE
HRasV12
Mcl1
Ras
actin
E
8d 4OHT
7d 4OHT, 1d none
Mcl1
phMEK1/2
MEK1/2
Lamin A/C
F
NAC
parthenolide
everolimus
PD184352
-
-
Ras
Cntr
Lamin A/C
Mcl1
G
cDNA, ng: 12 3 0.75 0.186 0
MCL1
APRT
Cntr Ras Cntr Ras Cntr Ras Cntr Ras
H
Ras: - + +
slipped: - - +
Lamin A/C
Cycl B1
Mcl1
p21
p16
I
siRNA: - siM siC
Lamin A/C
Mcl1
J
Cell death, x1000
days from mitotic slippage
Ras slipped siContr
Ras slipped siMcl1
Ras slipped
100% (staur)

Figure S3

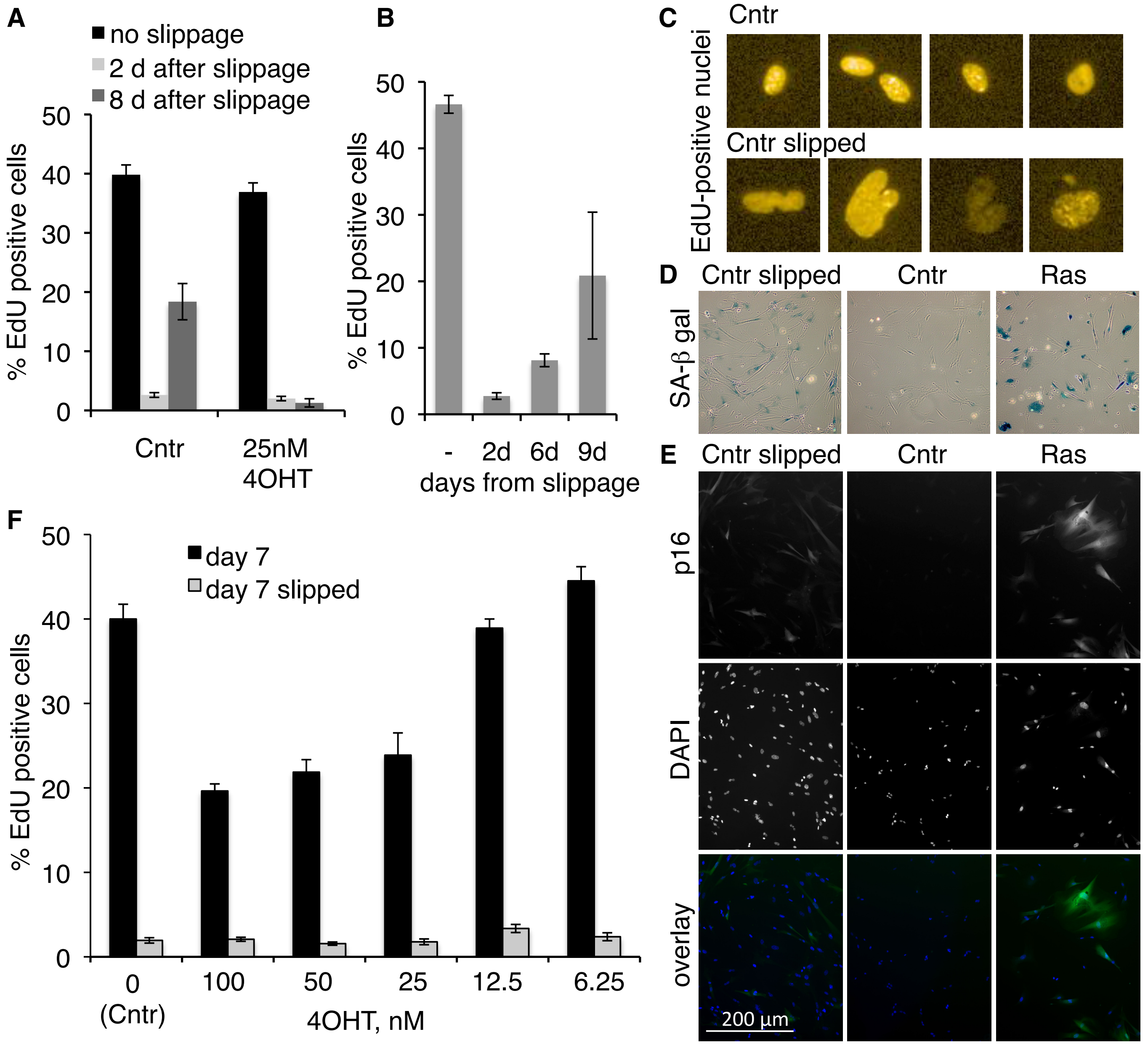
A
no slippage
2 d after slippage
8 d after slippage
% EdU positive cells
50
40
30
20
10
0
Cntr
25nM 4OHT
B
% EdU positive cells
-
2d
6d
9d
days from slippage
C
EdU-positive nuclei
Cntr
Cntr slipped
D
SA-β gal
Cntr slipped
Cntr
Ras
E
Cntr slipped
Cntr
Ras
p16
DAPI
overlay
200 µm
F
day 7
day 7 slipped
% EdU positive cells
0 (Cntr)
100
50
25
12.5
6.25
4OHT, nM

Figure S4

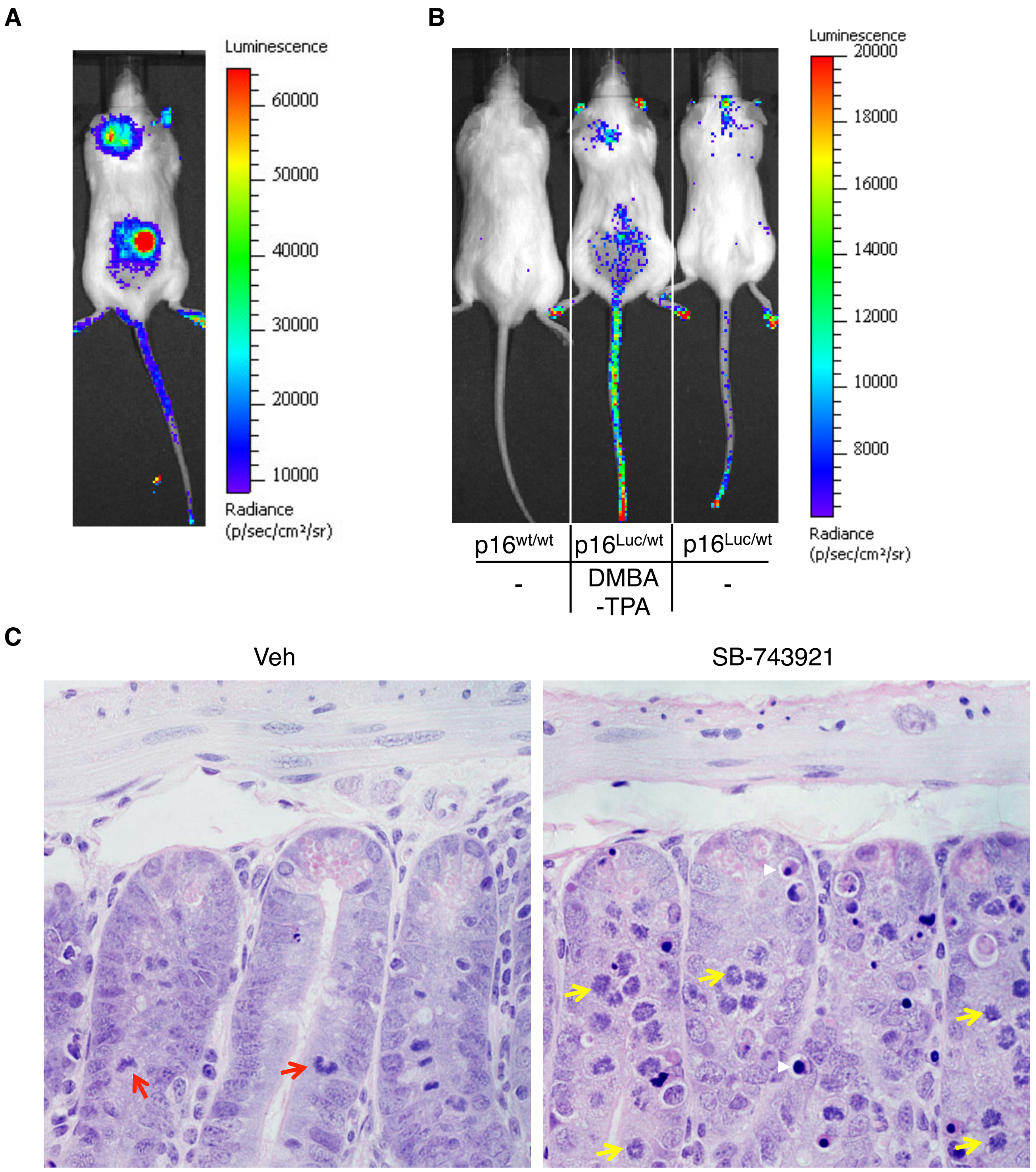
A
Luminescence
60000
50000
40000
30000
20000
10000
Radiance
(p/sec/cm²/sr)
B
Luminescence
20000
18000
16000
14000
12000
10000
8000
Radiance
(p/sec/cm²/sr)
p16wt/wt
p16Luc/wt
p16Luc/wt
-
DMBA
-TPA
-
C
Veh
SB-743921

Figure S5

Supplemental Figure legends

Figure S1, **Related to Figure 1. Induction of OIS and multinucleation in ERRAS cells. A.** Induction of expression of H-RasV12 and downstream MEK1/2 phosphorylation by tamoxifen (4OHT) in ERRAS cells but not in parental IMR90 cells. Untreated cells or cells treated with 4OHT for 1 day were lysed and blotted for Ras (endogenous and ectopic), MEK1/2 and phospho-Ser270/221 MEK1/2. Lamin A/C is a loading control. **B.** Decline in number of BrdU-incorporating cells upon H-RasV12 activation. ERRAS cells induced for indicated times were pulsed with 10 μM BrdU for 5 hours and stained with anti-BrdU antibodies. Proportion of BrdU-positive cells was calculated from 242-332 cells scored per time point. Aphidicolin-induced S-phase block during BrdU pulse was included as a negative control ("aph"). **C.** SA β-gal staining of control (left) or 14 days induced ERRAS cells (right). **D.** SAHF formation visualised by DAPI staining in 6 days induced ERRAS cells. Size bar 20 μm **E.** Kinetic of multinucleation shown as a percent of ERRAS cells with more than 2 nuclei, scored throughout first 10 days of H-RasV12 activation. Cells were fixed and stained with DAPI and antibodies against Lamin A/C and tubulin. 261-307 cells were scored per each timepoint. **F.** Immunofluorescence of IMR90 cells stably expressing GFP-Lamin A (middle, GFP fluorescence) stained with anti-lamin A/C antibodies (right) and DAPI (left). **G.** No effect of GFP-Lamin A expression on nuclear morphology. Nuclear morphology of untreated IMR90 cells or puromycin-selected IMR90 cells expressing GFP-Lamin A or GFP alone stained with DAPI and Lamin A/C antibody. Percent of cells with normal nucleus (norm), with lobulated nuclei (lobul), with nuclei containing a hole (with hole) or with two or more nuclei (MNC) scored from at least 230 cells for each condition. **H.** Example of GFP-lamin

A expressing ERRAS cells undergoing cell death. Selected frames from time-lapse sequence, with bright field images shown on a top and corresponding GFP signal shown on the bottom. Yellow arrows point to a cell undergoing apoptosis. Note that nuclear GFP-lamin A is detectable for several hours in cell that is morphologically at the late stage of apoptosis. Time stamp, hh:mm. See also Movie S2. **I.** Table summarising the multinucleation events observed in time-lapse images from GFP-Lamin A expressing control ERRAS cells or ERRAS cells induced for indicated times. Approximately 50-200 cells were observed in each time-lapse. *Average ± standard deviation was counted from 6 representative time-lapses for each condition. See also Movies S3-S6. **J.** Plot showing mean (± SD) number of cell divisions per time lapse, as in table H, last row. **K.** Percent of cell divisions (out of total number of dividing cells) that gave rise to MNC via slippage, calculated for indicated time intervals of H-RasV12 activation.

Figure S2, Related to Figure 4. Effects of DME treatment in control and induced ERRAS cells. A. DME arrests both 4 days H-RasV12-induced (Ras) and control (Cntr) ERRAS cells with monopolar spindles. Cells were incubated for 18 hours with DME, fixed and stained for microtubules (green), centrosome-associated pericentrin (red) and chromatin (DAPI, blue). Size bar 10 μm. **B.** H-RasV12 activation reduces apoptosis caused by DME treatment. Induced (Ras) or control (Cntr) ERRAS cells were incubated with DME for 17 hours before detecting apoptotic cells with NucView 488 caspase 3 substrate by flow cytometry. Cell debris were excluded from analysis. **C.** As in B, but only phospho-H3-positive cells in the DME-treated population were analysed. **D.** Activated H-RasV12 provides cells with a selective advantage in the presence of DME. IMR90

cells co-expressing inducible H-RasV12 and GFP (red or blue indicate a proportion of induced or control cells respectively) were mixed with equal number of GFP-negative parental cells (proportion indicated by grey). The proportion of GFP-positive cells in the mixed cultures was measured by flow cytometry immediately after mixing and for three consecutive days. In the presence of both 4OHT and DME, H-RasV12 expressing cells exhibited a selective advantage over parental H-RasV12 negative cells (row 1). However, when H-RasV12 was not induced (row 2), DME did not alter the ratio between the two cell types. In the absence of DME, both induced (row 3) and control (row 4) ERRAS cells had a slight growth disadvantage and were eventually outgrown by the parental IMR90 cells. **E.** Treatment of H-RasV12 expressing cells with DME leads to multinucleation. Induced ERRAS cells were treated with DME as in Fig 6A, and two days after drug removal (7 days of H-RasV12 induction) surviving cells were fixed, stained with DAPI and imaged using Operetta scanner. Insert shows a magnified fragment.

Figure S3, Related to Figures 4 and 6. Upregulation of Mcl1 by activated H-RasV12 contributes to survival of post-slippage cells. A. Increased Mcl1 level is sustained throughout the 2 weeks of OIS establishment in induced ERRAS cells. Days of 4OHT are shown at the top. Actin used as a loading control. **B.** Two independently derived lines of ERRAS IMR90 cells and one line of ERRAS BJ cells show similar level of Mcl1 upregulation upon 4 days of H-RasV12 activation with 4OHT. Level of Mcl1 in parental IMR90 and BJ cells is provided for comparison. Lamin A/C is used as loading control. **C.** No effect of 7 days of 4OHT on Mcl1 protein level in parental IMR90 cells. **D.** Upregulation of Mcl1 by constitutive H-

RasV12 in IMR90 cells. IMR90 were infected with H-RasV12 or control (pBABE) retrovirus and selected under puromycin for 8 days before cells were lysed and immunoblotted for Mcl1, Ras and actin (loading control). **E.** Increase in Mcl1 level requires continuous H-RasV12 expression. 4OHT was removed for 1 day from induced ERRAS cells at day 7 of induction, and Ras signalling (by phospo-MEK1/2) and Mcl1 level were compared to that in ERRAS cells under 8 days of continuous 4OHT-mediated activation. Lamin A/C is used as a loading control. **F.** Effect of indicated inhibitors on Mcl1 level. Lysates from control (Cntr) or 5 days induced ERRAS cells (Ras, -), or 5 days induced ERRAS cells treated with 10 mM NAC (for 17.5 hours), 20 μM parthenolide (for 17.5 hours), 50 μM Everolimus (for 4 hours) or 5 μg/ml PD184352 (for 4 hours) were separated on SDS-PAGE and immunoblotted for Mcl1 and lamin A/C (as a loading control). **G.** mRNA level of *MCL1* is increased in 4 days induced ERRAS cells, visualized by semi-quantitative PCR. The reactions were performed with decreasing amounts of source cDNAs as indicated. 443 bp band (top) detects *MCL1L* variant. *MCL1S* variant that would be recognized as 195 bp band is below detection limits. *APRT* is used as an internal control. **H.** High level of Mcl1 in slipped cells with activated H-RasV12. Cell lysates from control or 5 days induced ERRAS cells with or without DME-enhanced slippage were blotted for Mcl1 and indicators of cell cycle arrest (cyclin B1, p21 and p16), and lamin A/C as a loading control. **I.** Mcl1 depletion by siRNA in induced post-slipped ERRAS cells. Slipped ERRAS cells at day 6 of H-RasV12 induction / day 1 after slippage (asterisk in Figure 6A) were transfected with Mcl1-targeting (siM) or non-targeting (siC) siRNA or left untreated. Lysates were collected 4 days later and blotted for Mcl1 and Lamin A/C (as a loading control). **J.** Cell death kinetics measured as in Figure 6B in

induced slipped ERRAS cells after transfection with non-targeting (Ras slipped siContr, black) or Mcl1-targeting (Ras slipped siMcl1, grey) siRNA. Data were acquired simultaneously and at the same cell initial densities as data shown in Figure 6B, and identically analysed, allowing a direct comparison between these two datasets. Cell death kinetics of induced slipped ERRAS cells without transfection (Ras slipped, red, the same data as in 6B) and of staurosporine-treated control cells (100% (staur), green, the same data as in 6B) are shown for comparison.

Figure S4, Related to Figure 7. Mitotic slippage in IMR90 cells does not lead to senescence, but cooperates with H-RasV12 to induce senescence-associated cell cycle arrest.

A. Combination of slippage and subthreshold (for OIS onset) H-RasV12 expression induces effective cell cycle arrest. Percent of replicating cells measured by EdU incorporation at 2 (light grey) or 8 (dark grey) days after slippage (corresponding to 7 or 13 days after induction of H-RasV12) or without slippage (black) in control ERRAS cells (Cntr) or ERRAS cells treated with 25 nM 4OHT. Mean±SD from 7 replicates. **B.** Slippage alone does not induce long-term cell cycle arrest in control ERRAS cells. Percent of replicating cells measured by EdU incorporation at indicated times after DME-induced slippage are shown. Mean±SD from 7 replicates. **C.** Images of EdU-positive nuclei in control ERRAS cells (top) and control ERRAS cells 9 days after slippage (bottom), acquired and presented at the same magnification. Cells were pulsed with EdU for 3 hours before fixation and EdU detection. **D.** SA β-gal staining of control cells 9 days after slippage (left). Untreated uninduced control ERRAS (middle) and senescent

17 days induced ERRAS (right) are given as negative and positive control, respectively. **E.** p16 immunofluorescence in control ERRAS cells 9 days after slippage (left). Overlay colours: DAPI - blue, p16 - green. p16 staining in control (middle) and 14 days-induced (senescent, right) ERRAS cells are shown as negative and positive controls, respectively. Size bar 200 μm. **F**. Combination of mitotic slippage and low-level (sub-threshold for OIS onset) H-RasV12 expression induces effective cell cycle arrest in ERRAS cells (7 days time point). The experiment was conducted as in Fig 7B. Percent of replicating cells measured by EdU incorporation at 2 days after slippage (7 days of induction of H-RasV12) in control ERRAS cells (0, Cntr) or ERRAS cells treated with indicated concentrations of 4OHT. Mean±SD from 7 replicate experiments.

Figure S5, Related to Figure 7. SB-743921 induces mitotic arrest and activates the p16 promoter *in vivo*. A. Activation of p16 promoter-driven luciferase expression in $p16^{Luc/wt}$ transgenic mice upon 10 days of wound healing (at the back). The p16 promoter activity was measured by luminescence in the presence of D-licuferin substrate. The second area of luciferase activation (at the neck) is at the site of analgesic injection. The luminescence is shown on the relative scale, colour key to the radiance are provided on the right. **B.** Example of p16 promoter-driven luciferase expression activated by the DMBA-TPA protocol (3rd week, middle) in the $p16^{Luc/wt}$ mouse, as compared to untreated $p16^{Luc/wt}$ littermate (right). DMBA and TPA were applied to the shaved area at the back of the mouse. The area at the neck corresponds to the site of D-luciferin injection. The $p16^{wt/wt}$ mouse imaged in the same way is shown as a control (left). The p16 promoter activity was measured by luminescence in the presence of D-licuferin

substrate. The luminescence is shown on the relative scale, colour key to the radiance are provided on the right. Note that the scale is different from that shown in Fig 7F. **C.** SB-743921 causes mitotic arrest *in vivo*. Images of intestinal crypts from mice that received intraperitoneal injection of vehicle (left panel) or SB-743921 (right panel) for 3 days (Mon-Wed-Fri). H&E staining of paraffin-embedded intestine collected 6 hours after last injection revealed extensive mitotic arrest in SB-743921 but not vehicle treated mice. Red arrows point to the normal mitoses (vehicle-treated), yellow arrows point to representative drug-arrested mitotic cells (SB-743921-treated); white arrowheads point to representative apoptotic cells.

Supplemental Tables

Table S1 (Related to Figure 3). Alignment Statistics

Replicate	Sample	Raw Sequence reads	Read Length	Aligned Reads (% of Raw Sequence reads)	Non-Duplicate Reads (% of Aligned Reads)
1	Control	23,316,203	76PE	18,922,073 (81.15%)	15,772,985 (83.36%)
2	Control	22,254,971	76PE	18,149,692 (81.55%)	15,038,872 (82.86%)
3	Control	28,424,455	76PE	22,917,818 (80.63%)	18,570,931 (81.03%)
1	Ras	26,605,228	76PE	21,768,827 (81.82%)	17,900,549 (82.23%)
2	Ras	19,788,367	76PE	16,210,313 (81.92%)	13,526,592 (83.44%)
3	Ras	22,969,273	76PE	18,874,025 (82.17%)	15,417,674 (81.69%)

Supplemental Table legends

Table S1, related to Figure 3. RNA-seq alignment statistics.

Table lists the alignment statistics for each RNA-seq sample. The total reads generated, number (and %) of aligned reads, number (and %) of uniquely aligned reads (as a fraction of aligned reads) is given.

Table S2, related to Figure 3. RasV12 induced changes in mitotic transcripts. Table lists genes (first column) involved in mitosis-related process (indicated as "mitosis" in a second column, corresponding to heat map shown in Figure 3D for significantly changed genes in H-RasV12 expressing mitoses), organization and assembly of mitotic spindle (indicated as "Mitotic spindle" in a second column, corresponding to Figure 3F for significantly changed genes in H-RasV12 expressing mitoses), or chromatin maintenance or function (indicated as "Chromatin" in a second column, corresponding to Figure 3G for significantly changed genes in H-RasV12 expressing mitoses). Corresponding mean expression levels for uninduced (Control) and 4 days H-RasV12 activated (Ras) samples (from 3 replicas), log fold change and False Discovery Rate (q-value) are shown.

Supplemental Movies

Movie S1, Related to Figure 1. Mitosis. Typical example of normal mitosis (indicated by arrow) in a GFP-Lamin A-expressing 2 days-induced ERRAS cell. Bright field image (top) and corresponding GFP fluorescence (bottom) are shown in parallel. Time (hh:mm) is indicated at the top. Note the dispersal of nuclear envelope-associated GFP signal as cell enters mitosis (01:00 time point).

Movie S2, Related to Figure 1 and S1. Cell death. Typical example of death in a GFP-Lamin A-expressing ERRAS cell. Bright field images (left) and a corresponding GFP fluorescence (right) with cell of interest indicated by an arrow, are shown in parallel. Time (hh:mm) is indicated at the top. Note that nuclear envelope-associated fluorescence persists until after nuclear and cytoplasm compaction and cellular immobilisation (from 0:50 time point onwards) and is only lost simultaneously with the last (terminal) bleb (4:10 time point).

Movie S3, Related to Figure 1. Cell fusion. Typical example of cell fusion in induced GFP-Lamin A expressing ERRAS cell. Bright field images (left) and a corresponding GFP fluorescence (right) of 9 days induced ERRAS cells are shown in parallel. Time (hh:mm) is indicated at the top. Note that the two separate cells (indicated by arrows on GFP fluorescence images) fuse at 5:50 time point forming one binucleate cell that spreads (last frame) with two nuclei in close proximity.

Movie S4, Related to Figure 1. Nuclear fragmentation in interphase. An example of separation of lobulated nucleus in GFP-Lamin A-expressing 2 days-induced ERRAS cell to two nuclei during interphase. Bright field images (left) and a corresponding GFP fluorescence (right) are shown in parallel. Time (hh:mm) is indicated at the top. Note that nucleus of one of the cells (indicated by arrows in GFP images, a daughter cell generated from mitosis at 2:10) acquires 8-shaped form (21:20 time point), and eventually separates into two (possibly connected)

nuclei within one cell (last frame, right top corner), without intermittent loss of nuclear envelope fluorescence.

Movie S5, Related to Figure 1. Binucleation. Typical example of binucleation resulting from cytokinesis failure in induced GFP-Lamin A-expressing ERRAS cell. Bright field images (left) and corresponding GFP fluorescence (right) are shown in parallel. Time (hh:mm) is indicated at the top. Note the cell (indicated by arrow in the GFP time-lapse) that enters mitosis at 1:30 and forms 2 nuclei without cell division at 2:30. Cell spreads at 2:50 as binuclear and remains as such until the end of the time-lapse. While furrowing is not observed in this time-lapse sequence, we cannot exclude a transient furrow formed in the time between image acquisitions.

Movie S6, Related to Figure 1. Multinucleation. Typical example of multinucleation after prolonged mitotic arrest and slippage in induced GFP-Lamin A-expressing ERRAS cell. Bright field images (right) and a corresponding GFP fluorescence (left) are shown in parallel. Time (hh:mm) is indicated at the top. Note the cell (indicated by arrow in the GFP time-lapse) that enters mitosis at 01:10 time point and remains rounded for many hours until elongation (from approximately 9:30), constriction of cell body in several places (at 13:00) and its apparent fragmentation (from 15:30). Nuclear envelopes start to reform in several parts of the cell (from 17:30) and the cell spreads as multinucleate (visible from 22:40).

Movie S7, Related to Figure 1. Survival of multinucleated cell. An example of Ras-induced GFP-Lamin A expressing cells that remains viable for a long time after multinucleation via mitotic slippage. Bright field images (top) and a corresponding GFP fluorescence (bottom) are shown in parallel. Note the cell that undergoes mitotic slippage at 02:40 and remains alive, motile and multinucleated until the end of the time-lapse (indicated by arrow in the GFP-fluorescent images). Time (hh:mm) is indicated at the top.

.

Supplemental Experimental Procedures

Cell culture and treatment. IMR90 cells obtained from ATCC were maintained in DMEM supplemented with 20% fetal calf serum, 2 mM L-glutamine and 50 U/ml Pen-Strep, at 3% oxygen level. BJ cells obtained from ATCC were maintained in the same media except 10% fetal calf serum. ERRAS cells were generated by infecting low passage IMR90 or BJ cells with retrovirus carrying pLNC-Ras:ER (Barradas et al., 2009) (encoding ER-HRasV12, kind gift of Jesús Gil, Imperial College, London) that was packaged in Phoenix helper cells (Gary Nolan, Stanford University) as described (Swift et al., 2001) and cultured as above, except in phenol-free DMEM and in the presence of 0.5 mg/ml G418. For H-RasV12 activation, ERRAS cells were treated with 100 ng/ml (unless indicated otherwise) 4-hydroxytamoxifen (4OHT). Mcl1-overexpressing and appropriate control ERRAS cells were generated by infecting ERRAS cells with retrovirus carrying either pLZRS-HA-Mcl1 or pLZRS vector control (both plasmids kind gift of Stephan Tait, University of Glasgow), followed by Zeocin selection. GFP-lamin A expressing cells were generated by retroviral transduction of IMR90 or ERRAS cells with pBABE-puro-GFP-wt-Lamin A ((Scaffidi and Misteli, 2008), Addgene plasmid 17662). Retrovirus infected cells were selected and maintained under 1 μg/ml puromycin. GFP-expressing cells were generated by retroviral transduction of pQCXIP-GFP (Ye et al., 2007), followed by puromycin selection as above. For constitutive RasV12 expression, IMR90 cells were retrovirally transduced with pBabe-puro-H-RasV12 or control pBabe-puro (both plasmids kind gift of William Hahn, Dana Farber Cancer Institute, Addgene palsmid N9051) and selected with puromycin as above.

Staurosporine (Sigma), Everolimus (LC Laboratories), parthenolide (Sigma), PD184352 (Tocris Bioscience) were added to full growth media at indicated concentration for indicated time. N-acetylcysteine (NAC, Sigma) was dissolved in full media up to final concentration 10 μg/ml and filtered after adjusting pH to 7.2. For mitotic arrest, cells were treated with Eg5 inhibitor III (DME, Dimethylenastron, Calbiochem/Merck Cat.N. 324622) at 1 μM final concentration for indicated times. For harvesting mitoses, unattached cells arrested with DME for several hours were collected with 8-10 rounds of vigorous but careful pipetting in drug-containing growth media. Mitotic index was determined by visual scoring of cells with mitotically compacted chromosomes after 10 min incubation with PBS supplemented with small amounts of Hoechst DNA stain and Propidium Iodide, followed by fixation in 4% Paraformaldehyde in PBS. For harvesting cells slipped out of mitosis, collected mitoses were further incubated in full media containing DME for up to 3 days and any unattached cells and cell debris were thoroughly washed away before attached (slipped) cells were harvested by trypsinisation.

Apoptosis assay. Cells were incubated with media containing 5 μM NucView 488 Caspase 3 substrate (Biotium) for 40 min. Cells were collected, fixed in PBS containing 2% paraformaldehyde for 15 min and permeabilised with 0.1% triton X100 in PBS. After co-staining with required antibodies (e.g. anti-phospho-Histone H3 (Ser10), Millipore), the cellular level of activated caspase 3 substrate was measured by flow cytometry using BD FACSCalibur (BD Biosciences) and analysed using FlowJo software (Tree star).

siRNA. Cells were transfected with 50 nM Mcl1-targeting siRNA (siGENOME SMARTpool, M-004501-08-0005) or non-targeting siRNA (siGENOME Pool N2, D-001206-14-05, both from Dharmacon/Thermo Scientific) using Oligofectamine reagent (Invitrogen). For time-lapse imaging or cell death kinetics, cells seeded onto 24 well plates were transfected with 30 nM siRNA using 0.9 μl Lipofectamine 2000 (Invitrogen) per well 1-2 days before imaging.

Immunofluorescence. Cells cultured on glass coverslips were fixed with 4% paraformaldehyde in PBS or PHEM (60 mM PIPES, 4 mM $MgSO_4$, 25 mM HEPES, 10 mM K-EGTA, pH=6.9) and permeabilised with 0.1-0.5% triton X100 (for pericentrin, phospho H3 or p16 detection). Alternatively, cells were treated with 100% ice-cold methanol (for microtubules and lamin A/C detection) or 2.5% glutaraldehyde followed by 0.1% NaBrH4 in PBS (for visualisation of anaphase bridges). Immunostaining was performed in PBS containing 2% BSA, 0.1% triton X100 and sodium azide with following antibodies: anti-lamin A/C (Cell Signaling 2032S, 1:200), anti-alpha tubulin (DM1A, Sigma T6199, 1:1000), anti-pericentrin (AbCam ab4448, 1:500), anti-p16 (JC8, Santa Cruz, sc-56330, 1:100), and anti-phospho histone H3 (Ser10) (Millipore 06-570, 1:1000). Fluorescently-labelled secondary antibodies (Invitrogen) were used at 1:400 dilution. Cells were counterstained with 0.2 μg/ml DAPI (Sigma) in PBS and mounted with Prolong Gold mounting media (Invitogen).

Imaging and file processing

Immunofluorescently stained fixed cells were imaged either with epi-fluorescent Nikon Eclipse 80i microscope equipped with Hamamatsu ORCA-ER camera and

operated by MetaMorph software (Molecular Devices), or with Zeiss 710 (Axioimager) confocal microscope with 7 laser lines and Zeiss Quazar detectors, operated by ZEN software. B-gal and IHC sections were imaged using Olympus BX51 microscope equipped with Olympus DP70 digital camera and Cell*D imaging system (Olympus). Time-lapse microscopy was performed with Nikon Eclipse TE2000-E microscope equipped with Ocolab environmental chamber that maintains temperature, CO_2 and moisture. Images were acquired using CoolSNAP camera (Photometrics) operated by MetaMorph, at 10-30 min time intervals, as indicated. Routinely, 4 non-overlapping time-lapse sequences were acquired within each well of a multiwell plate with cells. For fluorescent time-lapse imaging, glass-bottom multiwell plates were used. Images acquired in MetaMorph were further processed in Image J that included linear color adjustment (identical between control and experimental samples, when used for comparison of signal intensity), cropping and combining single channels into a multi-colored or stacked image. For time-lapse microscopy, selected image sequences were assembled into movies (.avi file format) within Image J, converted into .m4v file format using HandBrake software and further converted into .mov file format in QuickTime player 7. Confocal images were processed using ZEN software, exported and further adjusted in Photoshop or Image J in a linear fashion.

Measuring duration of mitosis/mitotic arrest and frequency of mitotic slippage

Mitotically arrested cells in time-lapse images of DME-treated populations or untreated mitoses were tracked using Image J. The duration of mitotic arrest was calculated from the time of cell rounding (with signs of chromatin condensation

when visible) to either cell death (at the onset of extensive terminal blebbing) or slippage (cell flattening). For each experimental point, we scored all identifiable mitotic cells in 3-4 time-lapse sequences from the same well. The data were further analysed in Microscoft Excel.

Immunoblotting. Adherent cells were washed twice with PBS and boiled in 2xSDS lysis buffer containing 4% SDS, 12.5 mM TrisHCl pH 6.8 and 20% glycerol. Subsequently DTT was added up to 200 mM final concentration. Alternatively, cells were lysed in cold MEBC buffer containing 0.5% NP40, 50 mM Tris HCl, pH7.5, 100 mM NaCl, 5 mM EDTA, 5 mM K-EGTA. For detection of phospho-epitopes and/or unstable proteins, lysis buffer was supplemented with phosphatase and protease inhibitors (1 mM sodium orthovanadate, 50 ng/ml PMSF, protease inhibitor cocktail (Sigma P8340) used at 1:100 and phosphatase inhibitor cocktail (Sigma, P0044) used at 1:1000). Lysates were separated on 10% or 4-12% SDS-PAGE and transferred onto Whatman Protran BA85 nitrocellulose membrane (Sigma Aldrich). Total protein and protein markers were detected with reversible Ponceau S staining (Sigma, 81462) and immunoblotted in blocking solution containing 5% non-fat milk and 0.1% Tween 20 in PBS with following primary antibodies: anti-Ras (BD Transduction laboratories 610001, 1:500) anti-MEK1/2 (Cell Signaling/NEB 9122, 1:2000), anti-phospho Ser217/221 MEK1/2 (Cell signaling/NEB 9121, 1:1000), anti-p44/p42 MAPK (ERK1/2) (Cell Signaling 9102, 1:2500), anti-phospho Thr202/Tyr204 p44/p42 MAPK (ERK1/2) (Cell signaling/NEB 9101, 1:1000), anti-lamin A/C (Cell Signaling 2032S, 1:1000-1:2000), anti-XIAP (Cell Signaling 2045, 1:1000), anti-caspase 9 (Cell Signaling 9502S, 1:1000), anti-Mcl1 (BD

Pharmingen 559027 or Cell Signaling/NEB 9941, 1:1000 or 1:2000), anti-actin (Sigma 1978, 1:400000), anti-Bax, anti-Bid, anti-Bak (Cell Signaling/NEB 9942, all 1:1000); anti-Bcl2, anti-Bcl-xL (Cell Signaling/NEB 9941, all 1:1000), anti-cyclin B1 V152 (Santa Cruz sc-53236, 1:1000), anti-p21 (Santa Cruz sc-817, 1:1000), anti-p53 (DO-1, Santa Cruz sc-126, 1:1000) and anti-p16 (BD Pharmingen, 51-1325GR, 1:500). HRP-labeled secondary antibodies (Cell Signaling) were used at 1:5000 dilution in blocking solution, and detected with ECL kit (Pierce). For quantitative detection, IRDye 680 or 800 labeled secondary antibodies (Li-COR Biosciences) were used at dilution 1:5000 and detected with LiCOR scanner.

Protein stability measurements. Control or 1 day induced ERRAS cells were treated in triplicates with 10 μg/ml cycloheximide (Sigma) for indicated time and lysed in 2xSDS lysis buffer with protease and phosphatase inhibitors (see *Immunoblotting*). 5 μg lysates were separated on 10% PAGE, immunoblotted simultaneously for Mcl1 and actin using fluorescent secondary antibodies, scanned and quantified using LiCOR imaging system. Relative level of Mcl1 was calculated after normalization to actin signal intensity in the same sample. Mcl1 half-life value ($t_{1/2}$) was calculated from initial 80 min of exponential decay, as follows:

$$t_{1/2}=\ln(2)/k;\ \ k=(\ln(A_t)-\ln(A_{t+\Delta t}))/\Delta t,$$

where A_t – relative Mcl1 amount at time t, and Δt – time interval between two measurements.

Measurement of DNA synthesis. Cells seeded onto glass coverslips were pulsed for 5 hours with 10 μm BrdU, fixed with 4% PFA in PBS and permeabilised with 0.5% TritonX100 in PBS before DNase I treatment and immunostaining with anti-BrdU antibodies (DAKO). When indicated, 10 μM aphidicolin was added 1 hour before and during BrdU treatment. Cells were counterstained with DAPI and examined using Nikon Eclipse 80i microscope equipped with Hamamatsu ORCA-ER camera, operated by MetaMorph software (Molecular Devices). BrdU-positive and BrdU-negative nuclei were scored visually. Alternatively, cells seeded onto black-walled 96 well CellBIND plates (Corning) were pulsed with 10 μM EdU for 3 hours before fixation and EdU detection using Click-IT® EdU imaging kit (Life Technologies) according to the manufacturer instructions, and staining with DAPI. Plates were scanned with Operetta High Content Imaging system (PerkinElmer) at 10x magnification, with 11 images taken in each well, and percent of EdU positive cells per well was determined automatically using Harmony software. When required (eg for Fig S2E and Fig S4C), individual images were exported. We routinely seeded 8 wells per sample, of which one well was not pulsed with EdU and served as negative control.

PCR and qPCR.

Primers:

assay	target	sequence
PCR	*MCL1L*, *MCL1S* (Gao and Koide, 2013)	5'- ATCTCTCGGTACCTTCGGGAGC -3'<br>5'- CCTGATGCCACCTTCTAGGTCC -3'
PCR	*APRT*	5'-TGGAGATTCAGAAAGACGCCC-3'

		5’-GCCCTGTGGTCACTCATACTGC-3’
qPCR	*MCL1L*	5'- TAAGGACAAAACGGGACTGG -3'<br>5'- ACATTCCTGATGCCACCTTC -3'
qPCR	*GAPDH*	5’- GAGAGACCCTCACTGCTG -3’<br>5’- GATGGTACATGACAAGGTGC -3’

Total RNA was extracted using RNeasy plus kit (Qiagen), followed by DNase I treatment. cDNA was produced using oligo-dT primers. Semi-quantitative PCR was performed with indicated amount of cDNA, using Recombinant Taq DNA polymerase kit (Life Technologies, 10342020) as per manufacturer instructions with following parameters: 5 min at 95^0C, 30 cycles with 30 sec 94^0C, 30 sec 58^0C and 50 sec 72^0C each, with final elongation step for 7 min at 72^0C before cooling down to 4^0C. Products were electrophoretically separated in 2% agarose gel containing ethidium bromide and visualized using GeneGenuis gel imaging system (Syngene). Real-time PCR was performed using separately designed primers with the SYBR®-Green master mix (Life Technologies) on the BioRad Chromo4 thermo cycler. The qPCR products were validated both electrophoretically and by examining melting curves. Each reaction was done in triplicates, and obtained C(t) data for *MCL1* species and a housekeeping gene (*GAPDH*) were analysed using REST program to calculate changes in gene expression.

Mice

For generation of mouse pancreatic intraepithelial neoplasias (mPanINs) we used male and female progenies of mice heterozygous for *Lox-Stop-Lox-KRas*^{G12D}

transgene targeted to the endogenous Ras locus, crossed with pancreas-specific *Pdx1*-Cre mice. Resulting PDX1Cre$^{+/0}$::LSL-KRas$^{G12D/wt}$ mice on mixed C57Bl6 background (Hingorani et al., 2003) spontaneously developed pre-malignant PanINs (senescent lesions) that eventually give rise to metastatic adenocarcinomas (Hingorani et al., 2003; Morton et al., 2010). PDX1Cre$^{+/0}$::LSL-KRas$^{wt/wt}$ mice were used as a control.

For measuring activity of p16 promoter *in vivo*, we used males and females heterozygous Albino C57Bl6 luciferase reporter mice, in which gene body of endogenous p16^{INK4a} was replaced by a luciferase gene (Burd et al., 2013). Ras mutations were induced by ectopic application of 150 μl of 166 μg/ml 7,12-Dimethylbenz(a)anthracene (DMBA, Sigma Aldrich) in acetone to shaved dorsal skin of 7-9 week old mice, followed by 1 week rest before triweekly ectopic applications of 150 μl of 20.83 μg/ml 12-O-Tetradecanoylphorbol-13-Acetate (TPA, LC laboratories) in acetone, the protocol used for initiation of cutaneous two-stage carcinogenesis (Filler et al., 2007). Mice received the total of three 100 μl intaperitoneal injections of 0.5 mg/ml SB-743921 (Selleckchem) in 2% Cremophor, 98% sterile water pH 5.0 (final dose 2.5 mg/kg per injection) or equal volume of vehicle during the first week of TPA treatment, 6 hours after TPA application each.

In a pilot study, 7 weeks old FVB or C57Bl6 female mice received 2 weekly courses of 3 x 100 μl intaperitoneal injections of 0.5 mg/ml SB-743921 (Selleckchem) in 2% Cremophor, 98% sterile water pH 5.0 (final dose 2.5 mg/kg per injection) or equal volume of vehicle, and their health was monitored for another two weeks. Two mice per strain/treatment were sacrificed 6 hours after third injection, and the effectiveness of drugs was assessed by quantifying

mitotic and apoptotic indices in Haematoxylin & Eosin stained paraffin sections of intestinal crypts.

As a positive control for p16 activation, we used p16$^{Luc/wt}$ mouse 10-17 days after cutaneous wounding. The 5 mm diameter wound was created by dorsal skin incision of an anesthetized mouse. Subcutaneous injection of rimadyl prior the biopsy and oral rimadyl for 3 days after the biopsy was used for analgesia.

All mice were maintained in pathogen-free facilities proactive in environmental enrichment. All animal work was carried out according to UK Home Office regulations, in line with the EU directive 2010 and approved by ethical review (University of Glasgow).

In vivo luciferase detection.

Mice subcutaneously injected with 100 μl of 30 mg/ml RediJect D-Luciferin Bioluminescent Substrate (Perkin Elmer) were anesthetized with isoflurane and, 5 min after luciferin injection, imaged using IVIS Spectrum system (Caliper Life Sciences, Perkin Elmer). The radiance within equally sized dorsal regions (the areas subjected to the DMBA and TPA applications) was calculated using Living Image software (Caliper Life Sciences), before subtracting the background, derived by identical measurements of similarly treated p16$^{wt/wt}$ Albino C57Bl6 mouse (without a transgene).

Histopathology, immunohistochemistry and tissue immunofluorescence

4 μm sections cut from formalin fixed paraffin embedded blocks were baked onto poly-lysine slides for 60 minutes at 60°C. Sections were stained with

Haematoxylin & Eosin for morphological assessment or processed for immunohistochemistry (IHC) or immunofluorescence (IF) with the following antibodies: Melan A (Dako, M7196), phosho-ERK1/2 (Cell Signalling, 9101), Mcl1 (Proteintech 16225-1-AP). All sections were dewaxed in xylene, rehydrated through graded ethanols and washed in deionised water before heat-induced epitope retrieval using a Dako Pre-treatment module, either by heating for 25 minutes at 98^{0}C in 10mM Sodium Citrate pH6 retrieval buffer (Thermo, TA-250-PM1X) (for pERK1/2 IHC and for IF) or in 1mM Tris-EDTA pH9 retrieval buffer (TA-250-PM4X) (for Melan A); alternatively, antigen retrieval was performed in a microwave-heated pressure cooker for 5 minutes in citrate buffer pH6 (Dako) (for Mcl1).

Endogenous biotin and peroxidase activity were quenched using Dako's peroxidase block (S2023), and sections were further blocked in 10% normal goat serum (Dako) in antibody diluent (Dako) (for Mcl1) or in 5% normal goat serum (for IF). Primary antibodies applied for 45 minutes at room temperature or at 4^{0}C overnight (for Mcl1) were followed by appropriate secondary antibody: for pERK1/2 and Mcl1, Rabbit EnVision (Dako, K4003), for Melan A, Mouse EnVision (Dako, K4001). IHC sections were washed in TBST before application of Dako Liquid DAB (K4011) for 10 minutes to allow visualisation of the antigen. The reaction was terminated in deionised water before counterstaining the nuclei using Gills Haematoxylin. The sections were rinsed in water, dehydrated through graded ethanol, taken through xylene and then mounted using DPX mounting media (CellPath). The IF sections were stained in DAPI (Sigma) diluted 1:1000 in PBS. IHC staining was carried out on a Dako Autostainer Link48.

Statistical analysis

P-values were obtained using paired or unpaired two-tailed T-test, chi-square or two-way ANOVA, using PRISM or Microsoft Excel software.

RNA sequence and data analysis

RNA was isolated from cell pellets using RNAeasy kit (Qiagen), and DNA was removed using RNase-free DNase set (Qiagen). Quality of RNA was assessed on Agilent 2100 Bioanalyser using Agilent RNA 6000 Nano Kit (Agilen Technologis). To prepare the oligo-dT - based library, we used Illumina TruSeq RNA Sample Prep Kit, v2.0. The library was sequenced on a NextSeq500, using a NextSeq500 Mid Output kit 150 cycles flowcell. Reads were trimmed using Trim Galore (v0.3.0) (http://www.bioinformatics.babraham.ac.uk/projects/trim_galore/) and quality assessed using FastQC (v0.10.0) (http://www.bioinformatics.bbsrc.ac.uk/projects/*fastqc/*). Paired-end reads were aligned to the human genome (hg19) using a splicing-aware aligner (tophat2) (Kim et al., 2013). Duplicate reads were identified using the Picard tools (1.98) script mark duplicates (http://picard.sourceforge.net.). Reference splice junctions were provided by a reference transcriptome (Ensembl build 73), and novel splicing junctions were determined by detecting reads that span exons that are not in the reference annotation. Aligned reads were processed to assemble transcript isoforms, and abundance was estimated using the maximum likelihood estimate function (cuffdiff) from which differential expression and splicing can be derived (Trapnell et al., 2013). Genes of significantly changing expression were defined as FDR corrected p-value <0.05. For generation of RNA-

seq heatmaps, the FPKM value was calculated for each gene based on aligned reads, using Cufflinks (Trapnell et al., 2013). Z-Scores were generated from FPKMs. Hierarchical clustering was performed using the R library heatmap.2 and the distfun=’pearson’ and hclustfun=’average’. Principal component analysis (PCA) was performed using the FPKM values of all ensembl 73 genes of status 'known' and biotype 'coding'. For ontological analysis, differentially expressed genes were analysed using the DAVID (Huang da et al., 2009) tool Functional Annotation Chart, with the default human background. To calculate the significance of gene set enrichment, empirical p-values were generated using the USeq (v7.1.2) tool IntersectLists (Nix et al., 2008). The -t value used was 22,008, as the total number of genes of status 'known' and biotype 'coding' in ensembl genes 73. The number of iterations used was 10,000.

Supplemental References

Cruickshanks, H.A., McBryan, T., Nelson, D.M., Vanderkraats, N.D., Shah, P.P., van Tuyn, J., Singh Rai, T., Brock, C., Donahue, G., Dunican, D.S., *et al.* (2013). Senescent cells harbour features of the cancer epigenome. Nat Cell Biol *15*, 1495-1506.

Filler, R.B., Roberts, S.J., and Girardi, M. (2007). Cutaneous two-stage chemical carcinogenesis. CSH Protoc *2007*, pdb prot4837.

Gao, Y., and Koide, K. (2013). Chemical perturbation of Mcl-1 pre-mRNA splicing to induce apoptosis in cancer cells. ACS Chem Biol *8*, 895-900.

Gao, Y., and Koide, K. (2013). Chemical perturbation of Mcl-1 pre-mRNA splicing to induce apoptosis in cancer cells. ACS Chem Biol *8*, 895-900.

Huang da, W., Sherman, B.T., and Lempicki, R.A. (2009). Systematic and integrative analysis of large gene lists using DAVID bioinformatics resources. Nat Protoc *4*, 44-57.

Kim, D., Pertea, G., Trapnell, C., Pimentel, H., Kelley, R., and Salzberg, S.L. (2013). TopHat2: accurate alignment of transcriptomes in the presence of insertions, deletions and gene fusions. Genome Biol *14*, R36.

Nix, D.A., Courdy, S.J., and Boucher, K.M. (2008). Empirical methods for controlling false positives and estimating confidence in ChIP-Seq peaks. BMC Bioinformatics *9*, 523.

Scaffidi, P., and Misteli, T. (2008). Lamin A-dependent misregulation of adult stem cells associated with accelerated ageing. Nat Cell Biol *10*, 452-459.

Swift, S., Lorens, J., Achacoso, P., and Nolan, G.P. (2001). Rapid production of retroviruses for efficient gene delivery to mammalian cells using 293T cell-based systems. Curr Protoc Immunol *Chapter 10*, Unit 10 17C.

Trapnell, C., Hendrickson, D.G., Sauvageau, M., Goff, L., Rinn, J.L., and Pachter, L. (2013). Differential analysis of gene regulation at transcript resolution with RNA-seq. Nat Biotechnol *31*, 46-53.

Ye, X., Zerlanko, B., Kennedy, A., Banumathy, G., Zhang, R., and Adams, P.D. (2007). Downregulation of Wnt signaling is a trigger for formation of facultative heterochromatin and onset of cell senescence in primary human cells. Mol Cell *27*, 183-196.
