# Supplementary figures and images for: Mitotic Stress Is an Integral Part of the Oncogene-Induced Senescence Program that Promotes Multinucleation and Cell Cycle Arrest
Source: Cell Rep. 2015 Aug 20;12(9):1483–96. doi: 10.1016/j.celrep.2015.07.055 (PMC4562906; doi:10.1016/j.celrep.2015.07.055)

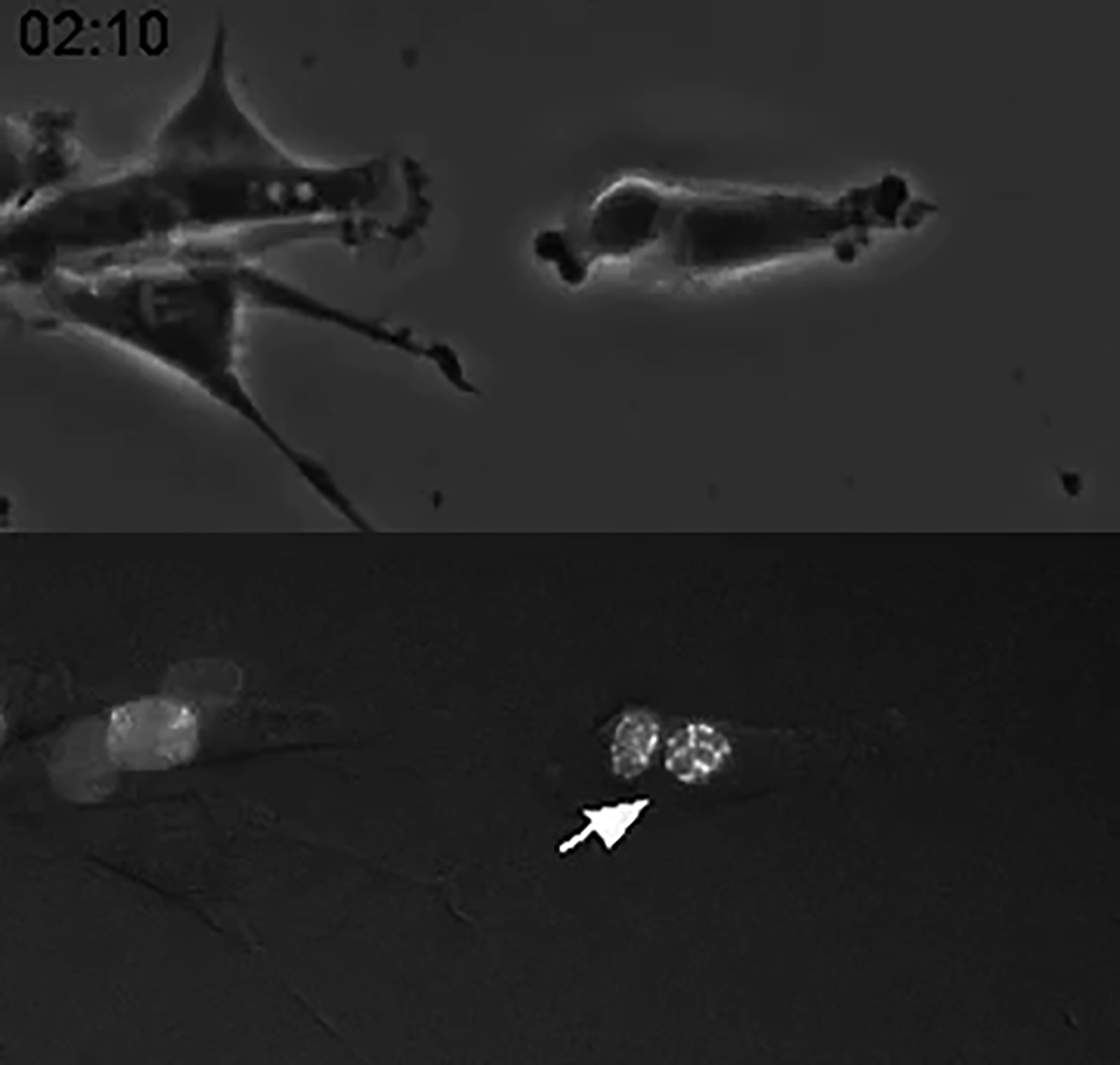

Supplement: Movie S1. Mitosis, Related to Figure 1 — Typical example of normal mitosis (indicated by arrow) in a GFP-Lamin A-expressing, 2-day-induced ERRAS cell. Bright-field image (top) and corresponding GFP fluorescence (bottom) are shown in parallel. Time (in hours:minutes, or hh:mm) is indicated at the top. Note the dispersal of nuclear-envelope-associated GFP signal as cell enters mitosis (at the 01:00 time point). [file mmc3.jpg]

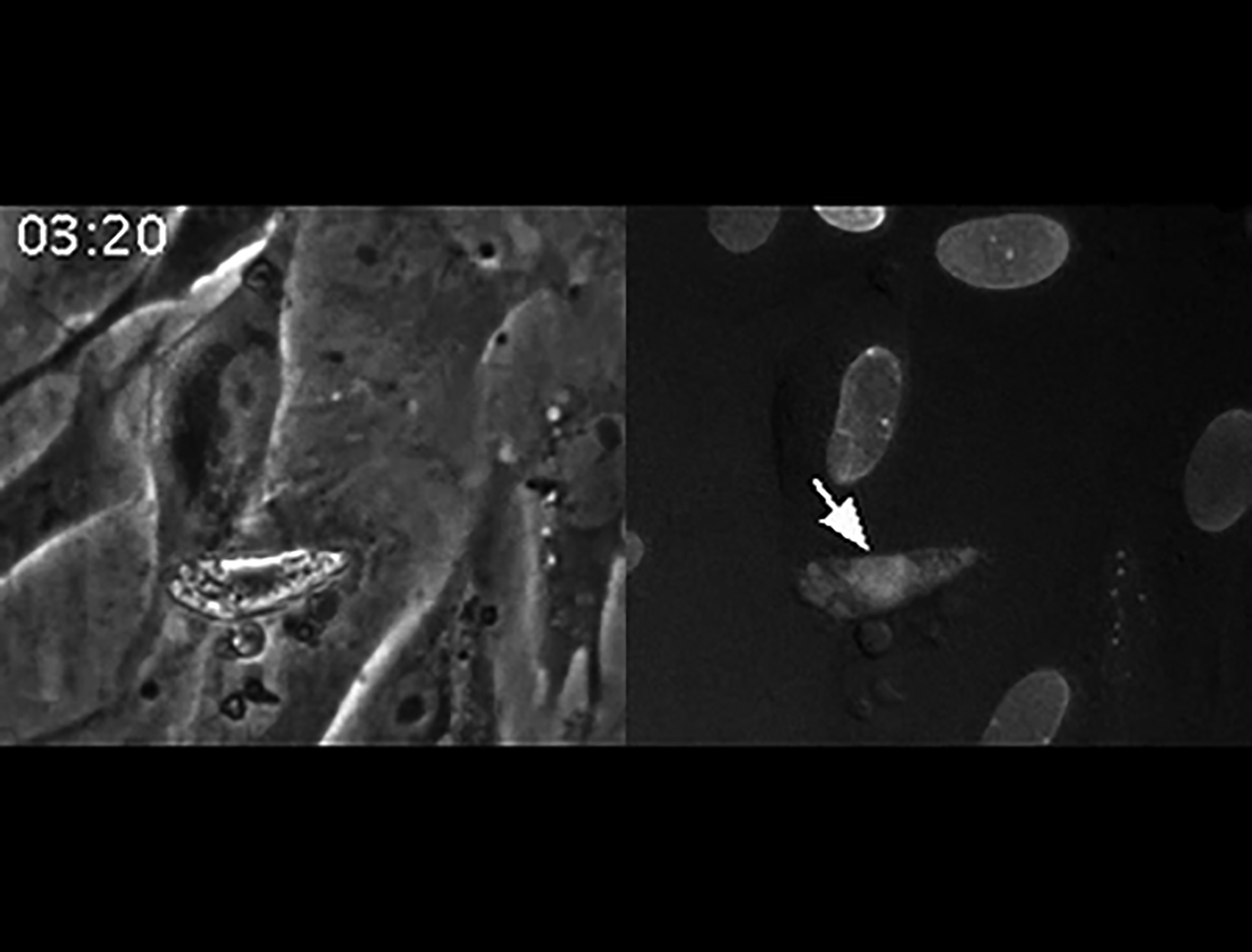

Supplement: Movie S2. Cell Death, Related to Figures 1 and S1 — Typical example of death in a GFP-Lamin A-expressing ERRAS cell. Bright-field images (left) and a corresponding GFP fluorescence (right) with cell of interest indicated by an arrow are shown in parallel. Time (in hours:minutes, or hh:mm) is indicated at the top. Note that nuclear-envelope-associated fluorescence persists until after nuclear and cytoplasm compaction and cellular immobilization (from the 00:50 time point onward) and is only lost simultaneously with the last (terminal) bleb (at the 04:10 time point). [file mmc4.jpg]

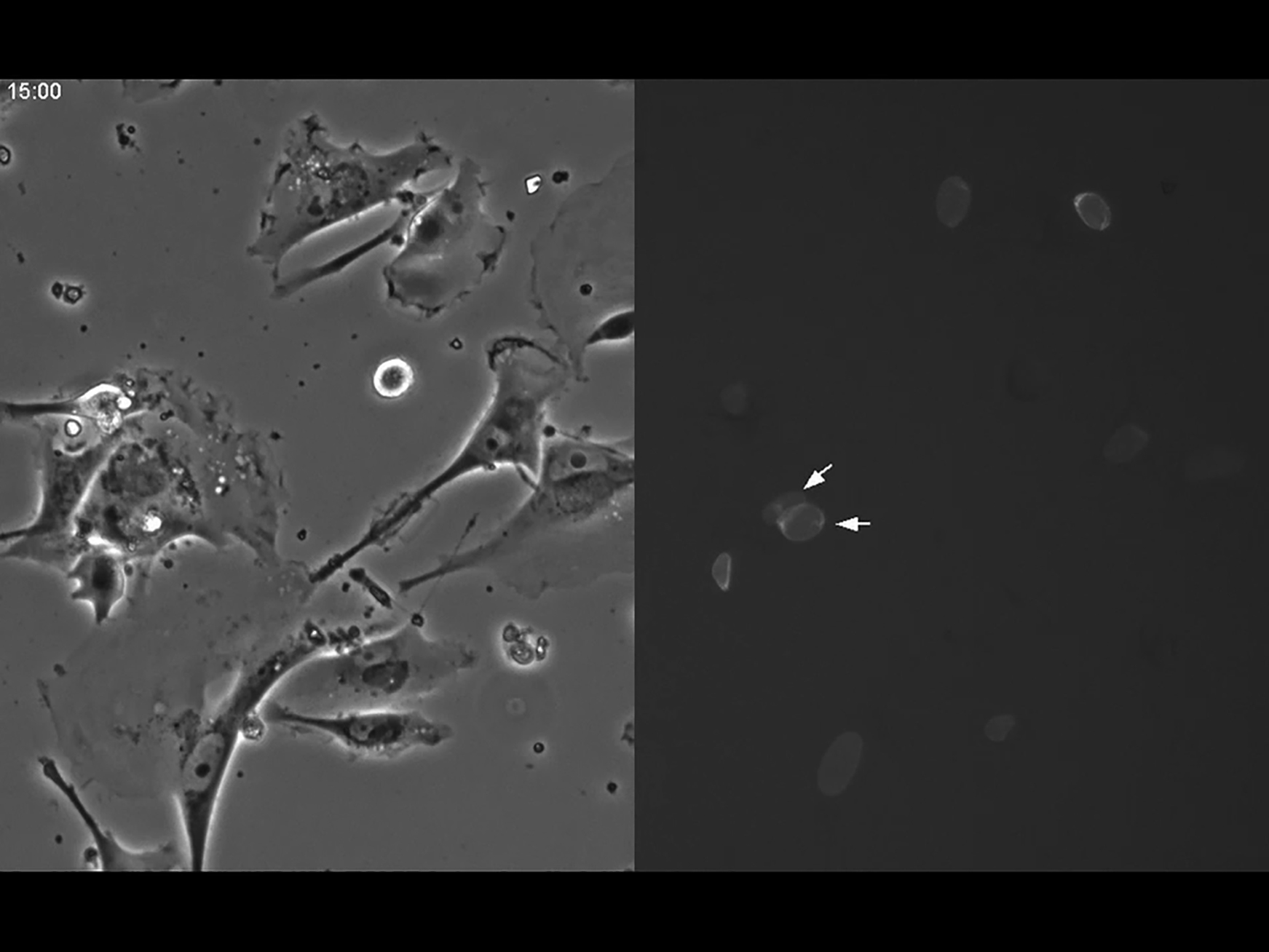

Supplement: Movie S3. Cell Fusion, Related to Figure 1 — Typical example of cell fusion in induced GFP-Lamin A-expressing ERRAS cell. Bright-field images (left) and a corresponding GFP fluorescence (right) of 9-day-induced ERRAS cells are shown in parallel. Time (in hours:minutes, or hh:mm) is indicated at the top. Note that the two separate cells (indicated by arrows on GFP fluorescence images) fuse at the 05:50 time point, forming one binucleate cell that spreads (last frame) with two nuclei in close proximity. [file mmc5.jpg]

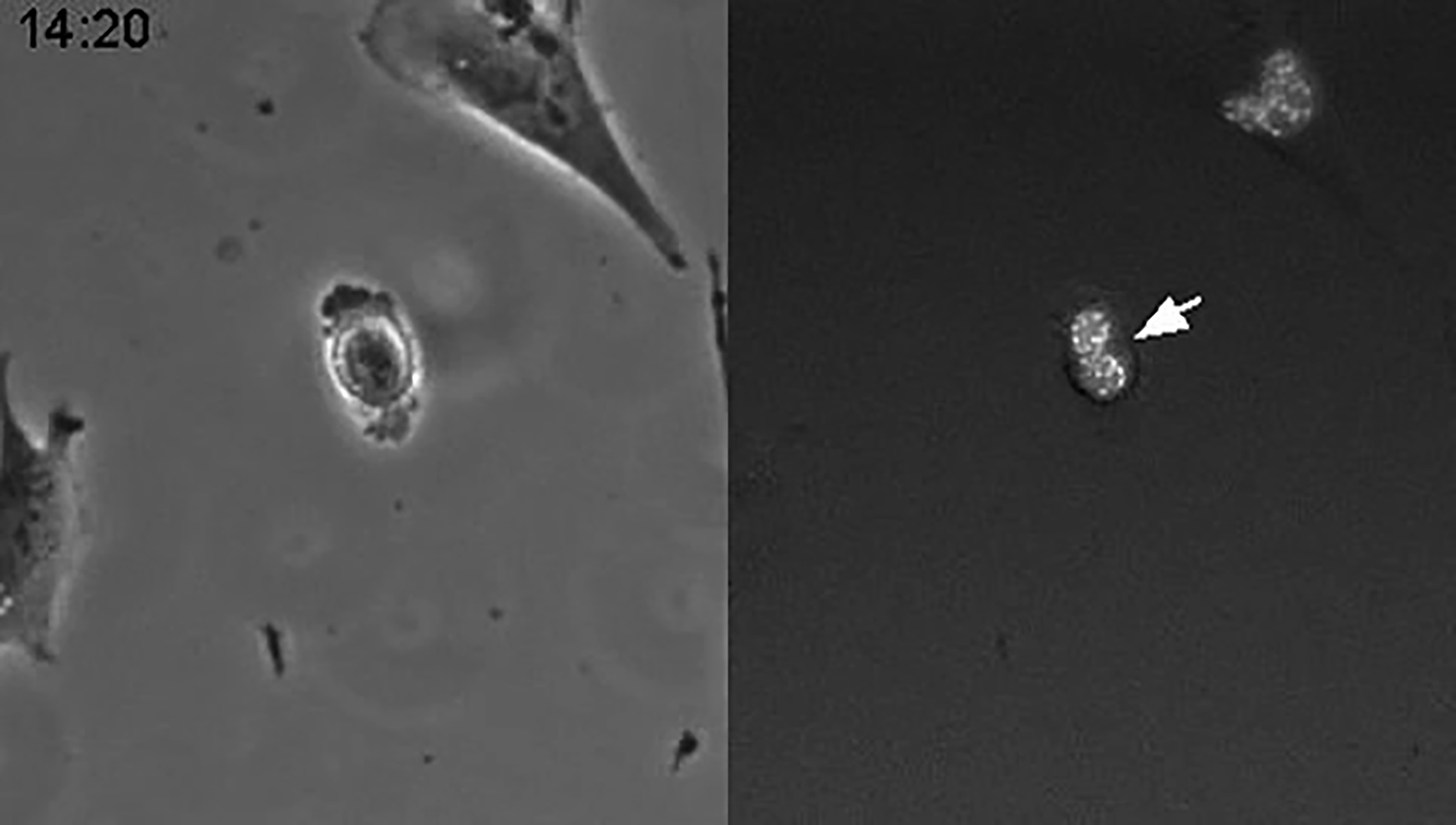

Supplement: Movie S4. Nuclear Fragmentation in Interphase, Related to Figure 1 — An example of separation of lobulated nucleus in GFP-Lamin A-expressing, 2-day-induced ERRAS cell to two nuclei during interphase. Bright-field images (left) and a corresponding GFP fluorescence (right) are shown in parallel. Time (in hours:minutes, or hh:mm) is indicated at the top. Note that the nucleus of one of the cells (indicated by arrows in GFP images, a daughter cell generated from mitosis at 02:10) acquires 8-shaped form (at the 21:20 time point) and eventually separates into two (possibly connected) nuclei within one cell (last frame, right top corner), without intermittent loss of nuclear envelope fluorescence. [file mmc6.jpg]

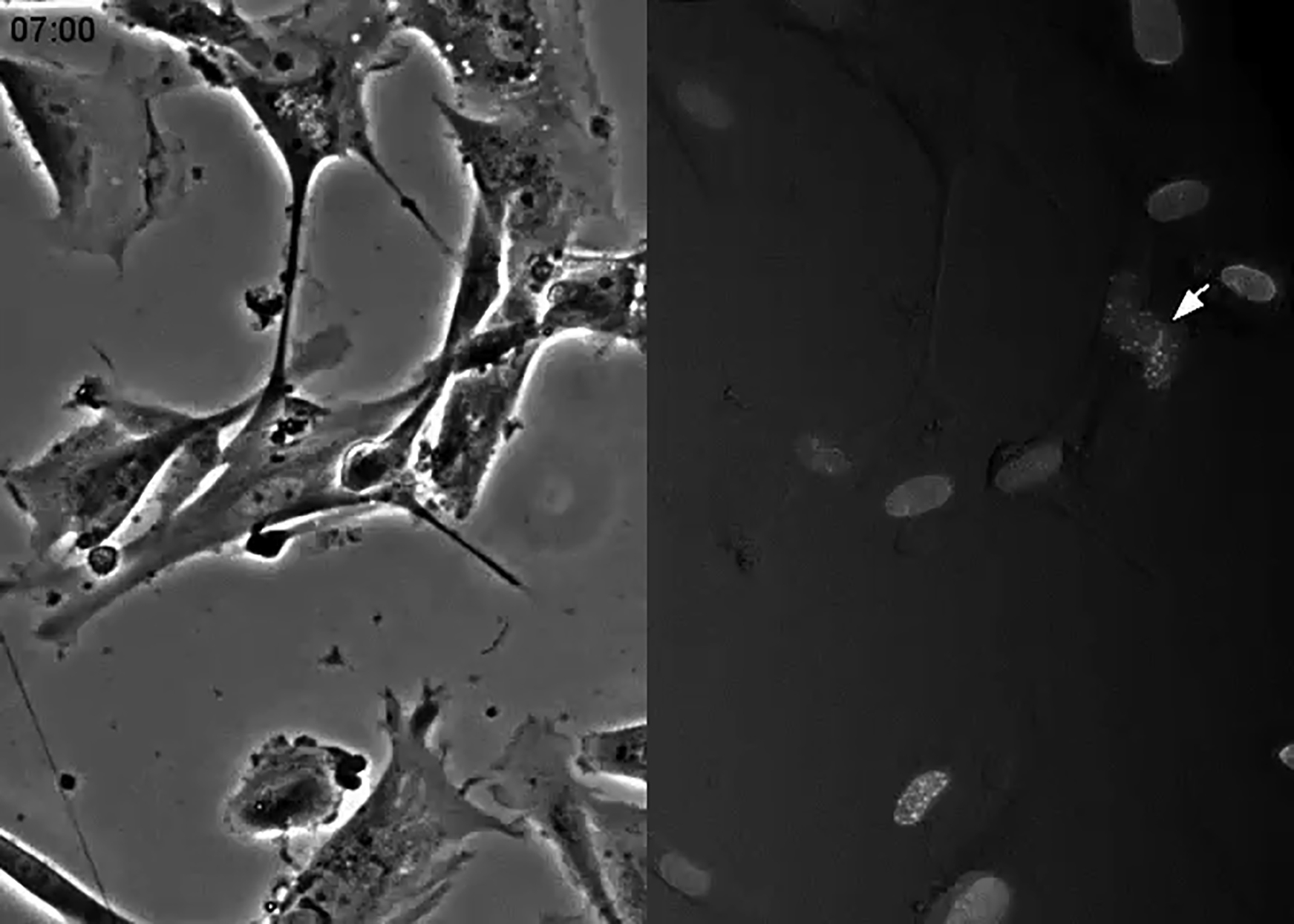

Supplement: Movie S5. Binucleation, Related to Figure 1 — Typical example of binucleation resulting from cytokinesis failure in induced GFP-Lamin A-expressing ERRAS cell. Bright-field images (left) and corresponding GFP fluorescence (right) are shown in parallel. Time (in hours:minutes, or hh:mm) is indicated at the top. Note the cell (indicated by arrow in the GFP time lapse) that enters mitosis at 1:30 and forms two nuclei without cell division at 02:30. Cell spreads at 02:50 as binuclear and remains as such until the end of the time lapse. While furrowing is not observed in this time-lapse sequence, we cannot exclude a transient furrow formed in the time between image acquisitions. [file mmc7.jpg]

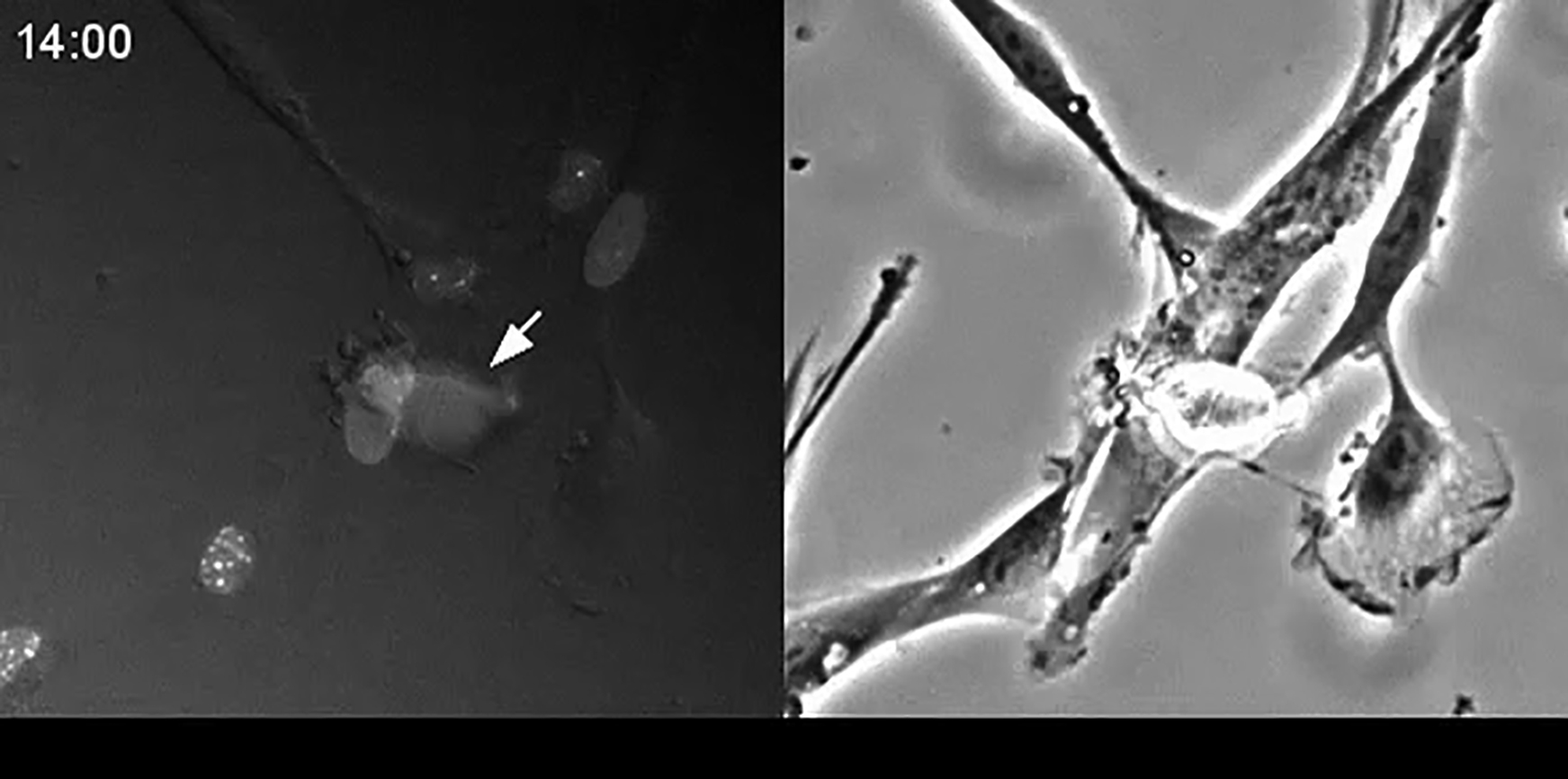

Supplement: Movie S6. Multinucleation, Related to Figure 1 — Typical example of multinucleation after prolonged mitotic arrest and slippage in induced GFP-Lamin A-expressing ERRAS cell. Bright-field images (right) and a corresponding GFP fluorescence (left) are shown in parallel. Time (in hours:minutes, or hh:mm) is indicated at the top. Note the cell (indicated by arrow in the GFP time lapse) that enters mitosis at the 01:10 time point and remains rounded for many hours until elongation (from approximately 09:30), constriction of the cell body in several places (at 13:00), and its apparent fragmentation (from 15:30). Nuclear envelopes start to reform in several parts of the cell (from 17:30) and the cell spreads as multinucleate (visible from 22:40). [file mmc8.jpg]

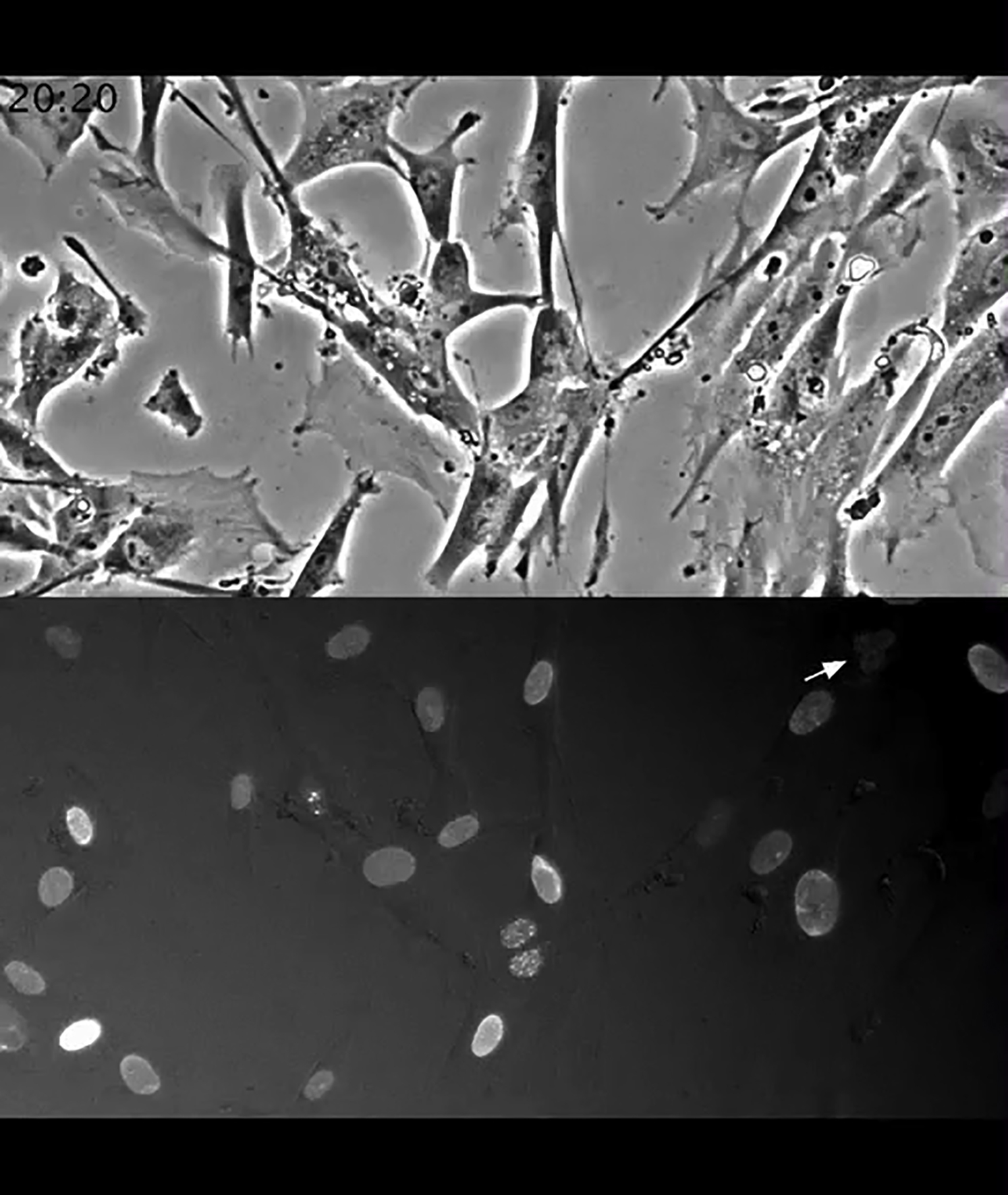

Supplement: Movie S7. Survival of Multinucleated Cell, Related to Figure 1 — An example of Ras-induced GFP-Lamin A-expressing cells that remain viable for a long time after multinucleation via mitotic slippage. Bright-field images (top) and a corresponding GFP fluorescence (bottom) are shown in parallel. Note the cell that undergoes mitotic slippage at 02:40 and remains alive, motile, and multinucleated until the end of the time lapse (indicated by arrow in the GFP-fluorescent images). Time (in hours:minutes, or hh:mm) is indicated at the top. [file mmc9.jpg]
