# Supplementary material for: Mitotic Stress Is an Integral Part of the Oncogene-Induced Senescence Program that Promotes Multinucleation and Cell Cycle Arrest
Source: Cell Rep. 2015 Aug 20;12(9):1483–96. doi: 10.1016/j.celrep.2015.07.055 (PMC4562906; doi:10.1016/j.celrep.2015.07.055)
Supplement: Document S2. Article plus Supplemental Information [file mmc10.pdf]

Cell Reports

Article

# Mitotic Stress Is an Integral Part of the Oncogene-Induced Senescence Program that Promotes Multinucleation and Cell Cycle Arrest

## Graphical Abstract

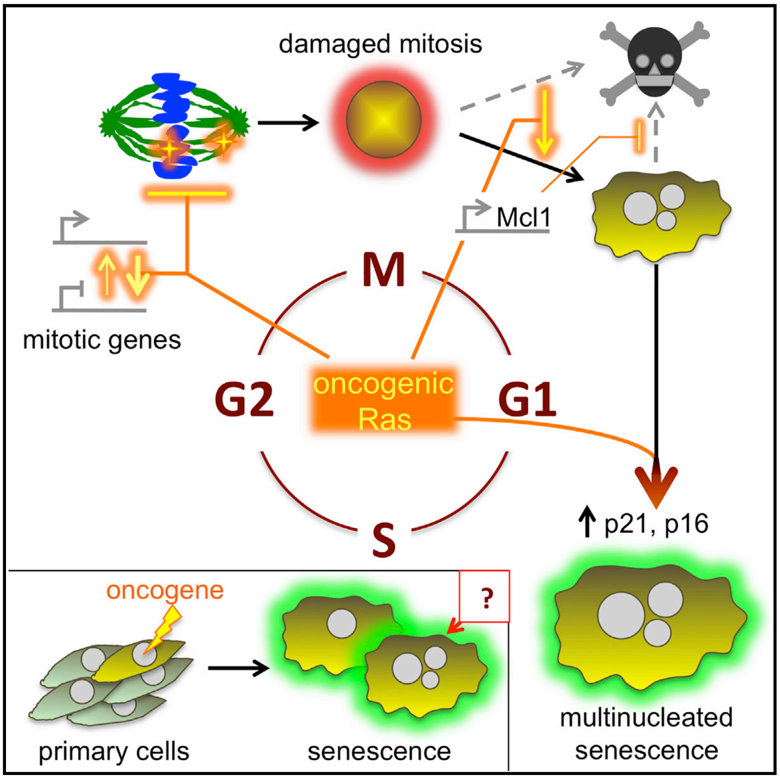


## Highlights

- Multinucleate OIS cells originate from aberrant mitotic progression
- H-RasV12-expressing cells in mitosis show aberrant expression of mitotic genes
- H-RasV12-induced mitotic stress and increase in Mcl1 allow mitotic slippage
- Mitotic slippage and oncogene signaling cooperate to establish senescence


## Authors

Dina Dikovskaya, John J. Cole, Susan M. Mason, ..., Shelley L. Berger, Karen Blyth, Peter D. Adams

## Correspondence

ddikovskaya@gmail.com (D.D.),
p.adams@beatson.gla.ac.uk (P.D.A.)



## In Brief

Dikovskaya et al. describe a mechanism of multinucleation during oncogene-induced senescence. The authors show that multinucleate senescent cells mostly originate from failed mitoses. They demonstrate that oncogene-induced mitotic defects, dysregulation of mitotic genes, and Mcl1-dependent apoptosis deficiency are the basis for multinucleation via mitotic slippage that further enhances senescence-associated cell-cycle arrest.


## Accession Numbers

GSE70668

Dikovskaya et al., 2015, Cell Reports *12*, 1483–1496
September 1, 2015 ©2015 The Authors
http://dx.doi.org/10.1016/j.celrep.2015.07.055

Cell Reports
Article

OPEN ACCESS
CellPress

# Mitotic Stress Is an Integral Part of the Oncogene-Induced Senescence Program that Promotes Multinucleation and Cell Cycle Arrest

Dina Dikovskaya,[1,*] John J. Cole,[1] Susan M. Mason,[2] Colin Nixon,[2] Saadia A. Karim,[2] Lynn McGarry,[2] William Clark,[2] Rachael N. Hewitt,[1] Morgan A. Sammons,[4] Jiajun Zhu,[4] Dimitris Athineos,[2] Joshua D.G. Leach,[5] Francesco Marchesi,[5] John van Tuyn,[1] Stephen W. Tait,[1] Claire Brock,[1] Jennifer P. Morton,[2] Hong Wu,[3] Shelley L. Berger,[4] Karen Blyth,[2] and Peter D. Adams[1,*]

[1]Institute of Cancer Sciences, CR-UK Beatson Laboratories, University of Glasgow, Glasgow G61 1BD, UK
[2]Beatson Institute for Cancer Research, Glasgow G61 1BD, UK
[3]Fox Chase Cancer Center, Philadelphia, PA 19111-2497, USA
[4]Department of Cell and Developmental Biology, Perelman School of Medicine, University of Pennsylvania, Philadelphia, PA 19104-6058, USA
[5]School of Veterinary Medicine, College of Medical, Veterinary and Life Sciences, University of Glasgow, Glasgow G61 1BD, UK
*Correspondence: ddikovskaya@gmail.com (D.D.), p.adams@beatson.gla.ac.uk (P.D.A.)
http://dx.doi.org/10.1016/j.celrep.2015.07.055
This is an open access article under the CC BY license (http://creativecommons.org/licenses/by/4.0/).

## SUMMARY

**Oncogene-induced senescence (OIS) is a tumor suppression mechanism that blocks cell proliferation in response to oncogenic signaling. OIS is frequently accompanied by multinucleation; however, the origin of this is unknown. Here, we show that multinucleate OIS cells originate mostly from failed mitosis. Prior to senescence, mutant H-RasV12 activation in primary human fibroblasts compromised mitosis, concordant with abnormal expression of mitotic genes functionally linked to the observed mitotic spindle and chromatin defects. Simultaneously, H-RasV12 activation enhanced survival of cells with damaged mitoses, culminating in extended mitotic arrest and aberrant exit from mitosis via mitotic slippage. ERK-dependent transcriptional upregulation of Mcl1 was, at least in part, responsible for enhanced survival and slippage of cells with mitotic defects. Importantly, mitotic slippage and oncogene signaling cooperatively induced senescence and key senescence effectors p21 and p16. In summary, activated Ras coordinately triggers mitotic disruption and enhanced cell survival to promote formation of multinucleate senescent cells.**

## INTRODUCTION

Cellular senescence is an important tumor suppressor mechanism and involves a stable proliferation arrest associated with an altered pro-inflammatory secretory pathway (Salama et al., 2014). In response to acquisition of an activated oncogene, primary human cells enter a proliferation-arrested senescent state called oncogene-induced senescence (OIS) (Braig et al., 2005; Chen et al., 2005; Collado et al., 2005; Michaloglou et al., 2005). Importantly, senescent cells, both in vitro and in vivo, frequently contain multiple nuclei in a single cell body (Salama et al., 2014). Indeed, appearance of multinucleated cells (MNCs) is a key feature of senescence (Vergel et al., 2010).

Pathways induced downstream of activated oncogenes include DNA replication stress and consequent DNA damage signaling. These effectors ultimately converge on the p16/pRB and p53/p21 tumor suppressor pathways (Salama et al., 2014). Senescence-associated proliferation arrest is generally thought to occur largely through a blockade to progression through G1 phase or early S phase (Campisi and d'Adda di Fagagna, 2007). Senescent cells can also be arrested in G2 (Mao et al., 2012), and more recent publications have documented the contribution of the premature activation of mitosis-specific E3-ligase, APC/C, to the onset of senescence (Johmura et al., 2014; Krenning et al., 2014). However, none of these mechanisms adequately explain the origin of multinucleate OIS cells.

Senescent cells within benign and/or early-stage neoplasia are at some risk of progression to malignancy if the senescence barrier is breached (Braig et al., 2005; Chen et al., 2005; Collado et al., 2005; Michaloglou et al., 2005). In this regard, human benign melanocytic nevi, neoplastic lesions of the skin composed largely of OIS melanocytes harboring activated *NRAS* or *BRAF* oncogenes (Gray-Schopfer et al., 2006; Michaloglou et al., 2005), frequently contain multinucleate melanocytes (Berlingeri-Ramos et al., 2010; Leopold and Richards, 1967; Patino et al., 2012; Savchenko, 1988). Multinucleate senescent melanocytes may harbor genome instability, a risk factor for malignancy (Fox and Duronio, 2013), and these cells have been proposed to give rise to highly proliferative, tumor-initiating stem-like cells (Leikam et al., 2015). Given that approximately 25% of melanomas are thought to arise in association with a pre-existing nevus (Smolle et al., 1999; Stolz et al., 1989) it is important to understand the origin of multinucleate, potentially pre-malignant, OIS cells.

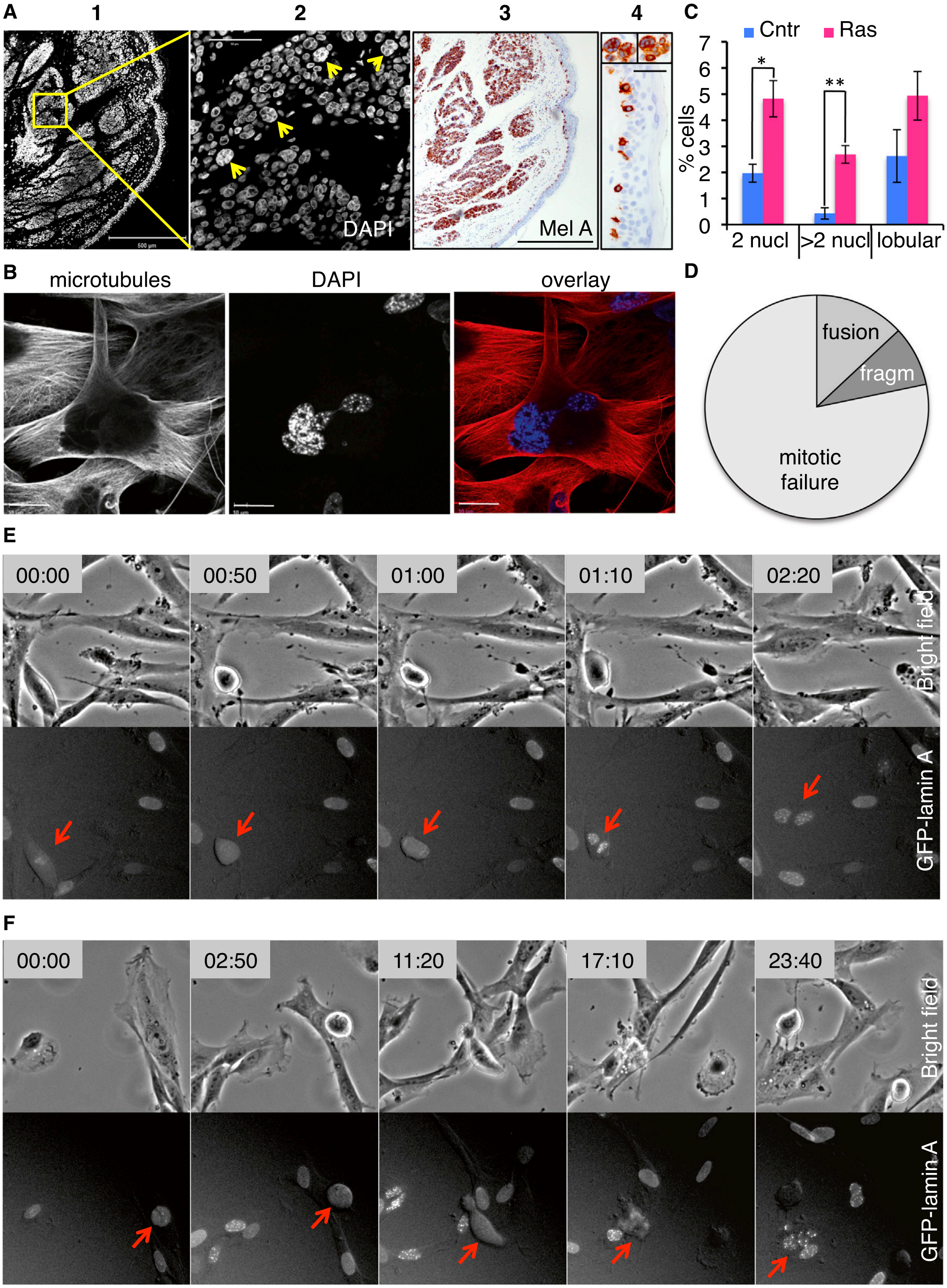


**Figure 1. Multinucleated OIS Cells Originate from Aberrant Mitosis**

(A) MNCs in human nevi. Dermal nevus-containing section of human skin stained with DAPI (panels 1 and 2) or for melan A (Mel A; panels 3 and 4). Panel 2 is a magnified insert with multinucleated nevus cells (arrows). Panel 4 shows a magnification of melan-A-positive multinucleated nevus cells (top) and a section of overlaying epidermis with mononucleated melanocytes (bottom). Scale bars, 500 µm for panels 1 and 3, 50 µm for panel 2, and 100 µm for panel 4. 17% of nevus cells (out of 334) and 0% epidermis melanocytes (out of 365) are multinucleated in this specimen.

*(legend continued on next page)*

Here, we show that activated RAS triggers two processes in pre-senescent primary cells, mitotic stress and upregulation of the anti-apoptotic protein Mcl1. These events together lead to extended mitotic arrest, ultimately followed by slippage out of mitosis to generate multinucleate proliferation-arrested senescent cells. We also present evidence that this process potentiates OIS, likely contributing to frequent multinucleation OIS cells observed in vivo.

## RESULTS

### OIS Is Accompanied by Multinucleation

To confirm previous reports of multinucleate senescent melanocytes in benign human nevi, we stained nevi with DAPI to detect DNA. This clearly revealed melan-A-positive nevus cells with multiple nuclei, while an overlaying epidermis contained only mononucleate melanocytes (Figure 1A). To investigate the origin of multinucleation in OIS, we generated primary human fibroblasts (IMR90) expressing tamoxifen-activatable oncogenic H-RasV12 fused to the estrogen receptor (ER) ligand-binding domain (Reuter and Khavari, 2006) (Figure S1A), hereinafter referred to as ERRAS cells. In this model, H-Ras signaling is readily induced with tamoxifen (hereinafter referred to as activated Ras or induced ERRAS cells), while uninduced cells serve as a control. As reported previously (Barradas et al., 2009; Lin et al., 1998; Reuter and Khavari, 2006; Young et al., 2009), activation of oncogenic H-RasV12 induced downstream MEK signaling (Figure S1A) and, after a transient proliferation burst, led to a gradual decrease in DNA synthesis (Figure S1B). Within 2 weeks, cell growth ceased, and cells displayed characteristic markers of senescence, such as senescence-activated β-galactosidase (SA-β-gal) and senescence-associated heterochromatic foci (SAHF) (Figures S1C and S1D) as previously described (Dimri et al., 1995; Narita et al., 2003). This was accompanied by statistically significant 2.5- and 6.2-fold increases in proportion of MNCs with two and more than two nuclei, respectively (Figures 1B, 1C, and S1E). Thus, this in vitro system recapitulates the multinucleation phenotype observed in OIS in vivo.

### Multinucleate OIS Cells Arise from Failed Mitoses

To delineate the process of multinucleation in OIS, we constitutively expressed a fluorescent-tagged nuclear envelope protein, GFP-Lamin A, in ERRAS cells. As expected, GFP fluorescence localized to the nucleus and outlined the nuclear envelope (Figure S1F), similar to endogenous Lamin A, with some nuclear foci as previously described (Hübner et al., 2006). Importantly, there was no difference in nuclear morphology between cells expressing GFP and GFP-Lamin A (Figure S1G). Fluorescent Lamin A facilitated continuous tracking of individual nuclei over several days, and mitotic cells (i.e., cells in M-phase of the cell cycle) were clearly identifiable by dispersal of the GFP-Lamin A fluorescence on breakdown of the nuclear envelope, rounded cell morphology, and compaction of chromatin (Movie S1). Mitotic cells were easily distinguished from cells undergoing apoptosis, in which nuclear GFP-Lamin A persisted until cessation of all cellular blebbing (Figure S1H; Movie S2). Long-term (3–4 days) time-lapse imaging of ERRAS cells expressing GFP-Lamin A revealed that, upon Ras activation, the majority of MNCs originate from failed mitosis, although a small number of cell fusions and fragmentation of lobulated interphase nuclei were also detected (Figures 1D and S1I; Movies S3 and S4). We observed two types of multinucleation events linked to mitosis. One was a binucleation upon cytokinesis failure after a mitosis of a normal duration (Figure 1E; Movie S5). It was also observed at comparable frequency in control cells (Figure S1I) and so was not specific to OIS. A second type, seen only in induced ERRAS cells (Figure S1I), followed a prolonged mitotic arrest and produced highly multinucleated cells (Figure 1F; Movie S6). It was accompanied by vigorous cell movement and was morphologically recognized as mitotic slippage (Brito and Rieder, 2006), an exit from mitosis into G1 without cell division (Rieder and Maiato, 2004). While mitotic potential of the entire population declined with the duration of Ras activation (Figure S1J), the percentage of mitoses that produced MNCs via slippage greatly increased (Figure S1K). MNCs often survived for at least several days (Figures 6B and S1E; Movie S7), likely contributing to multinucleation of senescent cells. We conclude that, during progression toward OIS, viable MNCs arise predominantly from failed mitoses.

### H-RasV12 Activation in Primary Cells Causes Mitotic Defects and Disruption of the Mitotic Gene Expression Program

Consistent with observed mitotic failure in induced ERRAS cells, we found a significant increase in spindle defects (Figures 2A and 2B) and abnormal chromatin morphology (Figures 2B–2E) in prometaphase and metaphase cells 3–5 days after Ras activation. Specifically, mitotic spindles were either misshapen or had low microtubule content (Figure 2A; data not shown), and prometaphase and metaphase chromatin appeared de-compacted (Figures 2C–2E; compare the diameters of individual chromosomes indicated in Figures 2C and 2D). Furthermore, the proportion of anaphases with either lagging chromosomes or anaphase bridges (Figure 2F) was significantly higher in cells after Ras activation (Figure 2G). Thus, H-RasV12 activation triggers mitotic abnormalities in primary human fibroblasts.

(B) Multinucleated senescent 12-day-induced ERRAS cell, stained for microtubules (left, red on overlay) and DAPI (middle, blue on overlay). Scale bars, 30 μm.
(C) 15-day ERRAS induction (Ras) increases the percentage of MNC with two nuclei, more than two nuclei, and lobular nuclei. Data indicate means ± SEM from three independently derived ERRAS cell populations. *p = 0.021; **p = 0.005 (paired Student's t test). Cntr, control.
(D) Origin of multinucleation in GFP-Lamin A-expressing ERRAS cells observed throughout 12 days of Ras induction and classified as originating from mitotic failure (light gray), cell fusion (medium gray), and interphase fragmentation (fragm, dark gray). n = 23 multinucleation events (see also Figure S1I).
(E and F) In (E), cytokinesis failure leading to binucleation is shown. (F) Prolonged mitotic arrest followed by slippage, generating a highly multinucleated cell. For (E) and (F), bright-field image (top) and corresponding GFP fluorescence (bottom) are shown at selected times (in hours:minutes, or hh:mm). Arrows indicate cells undergoing multinucleation.
See also Figure S1 and Movies S1, S2, S3, S4, S5, S6, and S7.

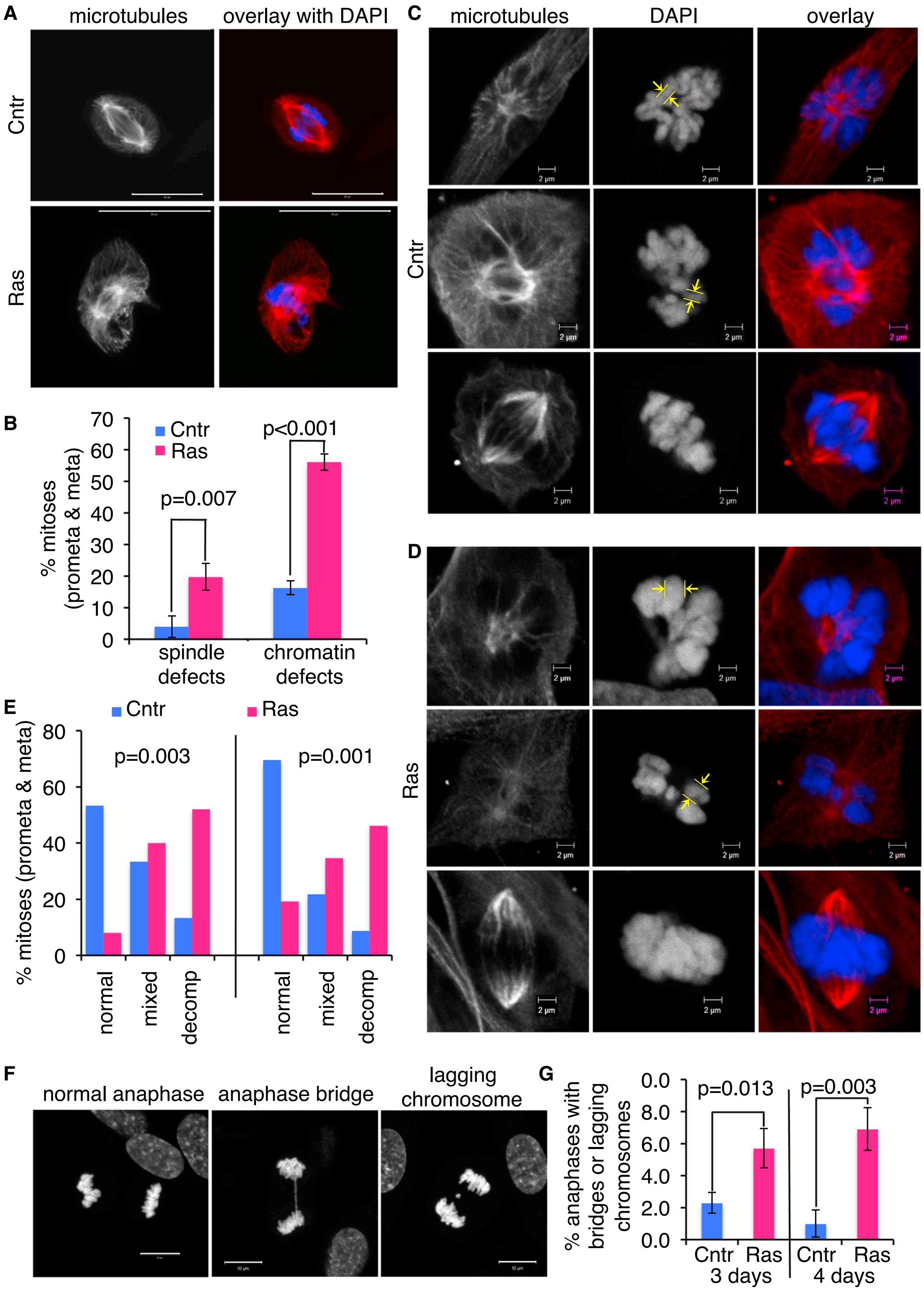


**Figure 2. Mitotic Abnormalities in Pre-senescent Cells with Activated H-RasV12**

(A) Mitotic spindles in control (top, Cntr) and induced (bottom, Ras) ERRAS cells, stained for microtubules (left panels, red in overlay) or DAPI (blue in overlay). Scale bars, 20 µm.

*(legend continued on next page)*

To identify potential cause(s) of mitotic defects during the establishment of OIS, we performed RNA sequencing (RNA-seq) gene expression profiling of cells captured in mitosis 4 days after Ras activation (Figure 3A). Our isolation procedure yielded >90% mitotic cells from both induced and control ERRAS cells. RNA-seq revealed that Ras induction significantly altered the abundance of approximately 2,000 gene transcripts in mitotic cells (Figure 3B). Principal-component analysis (PCA) showed that replicates were highly consistent (Figure 3C). Consistent with Ras-induced defects in mitosis, we found that, out of 371 genes included in mitosis-related Gene Ontology (GO) terms, 74 were significantly (5% false discovery rate [FDR]) altered in mitoses with activated Ras (Figure 3D; Table S2). This constituted a statistically significant (empirical p value < 0.0001) 2.17-fold enrichment of alterations in this gene set over randomly expected changes. Furthermore, out of 328 transcripts that were highly up- or downregulated in normal mitosis compared to the unsynchronized control ERRAS cell population [that are potentially relevant to mitotic processes (Cho et al., 2001)], 64 (approximately 20%) were significantly (5% FDR) altered by H-RasV12 in mitotic cells (2.11-fold increase over random, empirical p value < 0.0001). More importantly, mitotic spindle-related gene ontologies (namely, "mitotic spindle organization," "spindle localization," and "establishment of spindle localization") were the top three most altered GO terms (Figures 3E and 3F; Table S2), consistent with the diverse spindle defects in induced ERRAS cells (Figures 2A and 2B). Changes in the spindle-related gene set were, significantly (empirical p < 0.0001), 3.18-fold enriched over randomly expected. In line with the observed chromatin defect in these cells (Figures 2C–2E), the expression of chromatin regulators was also significantly (5% FDR) changed (Figure 3G; Table S2) (1.26-fold enrichment, empirical p = 0.0318). Underscoring the specificity of these changes, the spindle checkpoint GO term was not significantly altered in Ras-induced mitotic cells (empirical p = 0.75), consistent with efficient mitotic arrest in these cells (see Figures 4B and 4C). Thus, H-RasV12 activation dysregulates expression of a specific subset of mitotic genes linked to the observed mitotic abnormalities in pre-senescent cells.

## Activated H-RasV12 Suppresses Death and Promotes Slippage out of Aberrant Mitosis to Generate MNCs

To examine the fate of defective mitoses in primary cells with activated Ras, we induced widespread mitotic defects with an Eg5 inhibitor, Dimethylenastron (DME), which prevents centrosome separation and formation of a bipolar spindle. In many cell types, this engages the spindle checkpoint to arrest cells in mitosis and eventually leads to cell death (Rath and Kozielski, 2012). DME induced efficient mitotic arrest with characteristic monopolar spindles in both control and induced ERRAS cells (Figure S2A), consistent with an intact spindle checkpoint (discussed earlier). However, cells with activated Ras were much less sensitive to the cytotoxic effect of DME (Figure 4A). Ras attenuated DME-induced caspase-3 activation among all cells (Figure S2B) and specifically in phospho-H3-positive mitotic cells (Figure S2C). Consistently, DME treatment conferred a selective advantage on pre-senescent induced ERRAS cells over parental IMR90 cells in mixed culture (Figure S2D). Live cell imaging of individual mitoses revealed that activated Ras extended mitotic arrest and delayed death in mitosis under DME (Figures 4B and 4C). In addition, Ras activation dramatically increased the proportion of cells that exited mitotic arrest via mitotic slippage (Figure 4B). Consistent with nuclear fragmentation during slippage (Zhu et al., 2014), induced ERRAS cells that survived DME treatment displayed widespread multinucleation (Figure S2E). We conclude that activated H-RasV12 confers resistance to apoptosis triggered by aberrant mitosis. Instead, such cells tend to slip out of mitosis to generate MNCs.

## H-RasV12 Protects from Mitotic Death via ERK-Dependent Increase in Mcl1

To find the cause of Ras-induced resistance to mitotic cell death, we assessed the accompanied changes in regulators of mitotic cell death and apoptosis in general. In ERRAS cells, one such protein, Mcl1, showed a marked and sustained upregulation upon Ras activation (Figures 4D, 4E, and S3A). A more modest rise in XIAP level was also detected (Figure 4D), as previously published (Liu et al., 2005). Expression of other apoptotic regulators—namely Bid, Bax, Bak, Bcl-2, and Bcl-xL—was not altered by activated Ras. Mcl1 also increased upon tamoxifen-induced activation of ER-RasV12 in BJ fibroblasts (Figures S3B and S3C) or upon retroviral transduction of IMR90 cells with constitutively active H-RasV12 (Figure S3D). Importantly, cells arrested in mitosis also displayed the Ras-mediated rise in the level of Mcl1 (Figure 4F).

Elevated Mcl1 required continuous H-RasV12 signaling, since tamoxifen withdrawal from induced ERRAS cells reduced Mcl1 protein level, concomitant with the decline in MEK1/2 phosphorylation (Figure S3E). Importantly, Mcl1 was also reduced by MEK inhibitor PD184352, which abolishes phosphorylation of extracellular signal-regulated kinases 1 and 2 (ERK1/2) downstream of activated Ras (Figures 4G and S3F). In comparison, inhibition

(B) Quantification of chromatin and spindle defects (exemplified in A and D) in prometaphases (prometa) and metaphases (meta) of control (Cntr) or 5-day-induced (Ras) ERRAS cells. Data indicate means ± SD and p values from three replicates, 53–65 mitoses each.

(C and D) Normal mitotic chromatin (C) or Ras-induced chromatin defect (D) in mitotic cells from prophase to metaphase. Single optical sections of 3D confocal images of control (Cntr) (C) or 3-day-induced ERRAS (D) cells stained for microtubules (left panels, red in overlay) or DAPI (middle panels, blue in overlay). Scale bars, 2 μm. Yellow frames show the visible width of chromosomes.

(E) Quantification of chromatin decompaction in high-resolution 3D confocal images in two independent experiments. DAPI-stained prometaphases (prometa) and metaphases (meta) in control (Cntr) or 3-day-induced (Ras) ERRAS cells were scored for decompacted (decomp), partially decompacted (mixed), or normal chromatin (normal). Two independent experiments are shown. 15–26 mitoses per condition per experiment. p values for distribution changes calculated with two-way ANOVA.

(F) Normal anaphase (left), aberrant anaphase with bridge (middle), or anaphase with a lagging chromosome (right). Scale bars, 10 μm.

(G) Quantification of anaphase defects, shown in (F), in 3- and 4-day-induced (Ras) or control (Cntr) ERRAS cells. Data indicate means ± SD and p values from three replicas, 155–532 anaphases each.

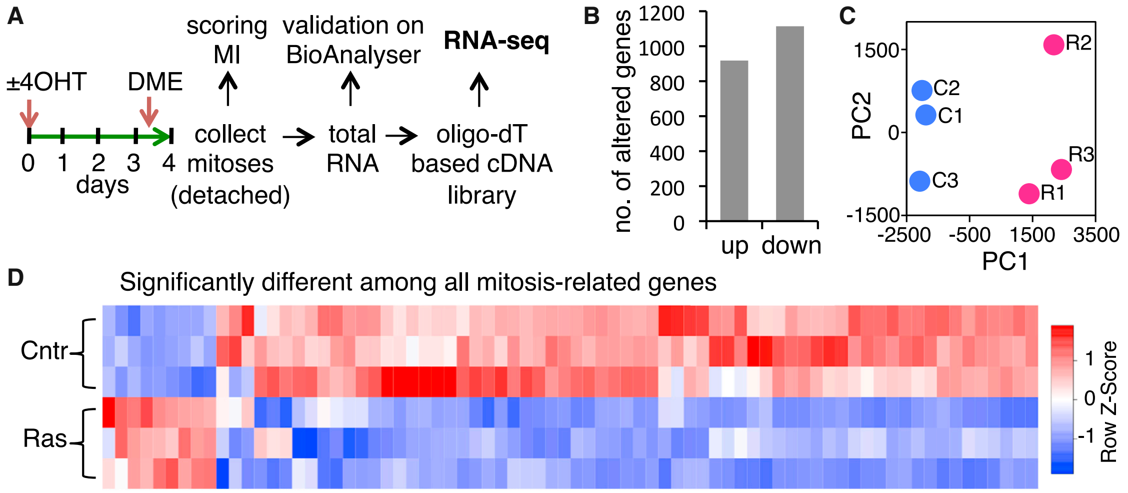


E

| GO Name | No. of Genes | Fold Enrichment | FDR (%) |
|---|---|---|---|
| mitotic spindle organization | 9 | 5.78 | 0.08 |
| spindle localization | 6 | 5.78 | 3.51 |
| establishment of spindle localization | 6 | 5.78 | 3.51 |
| nucleotide-excision repair, DNA gap filling | 8 | 4.53 | 1.79 |
| collagen fibril organization | 13 | 4.32 | 0.03 |
| positive regulation of positive chemotaxis | 8 | 4.05 | 3.81 |
| regulation of positive chemotaxis | 8 | 4.05 | 3.81 |
| positive regulation of mitosis | 10 | 3.85 | 1.10 |
| positive regulation of nuclear division | 10 | 3.85 | 1.10 |
| base-excision repair | 10 | 3.57 | 2.07 |

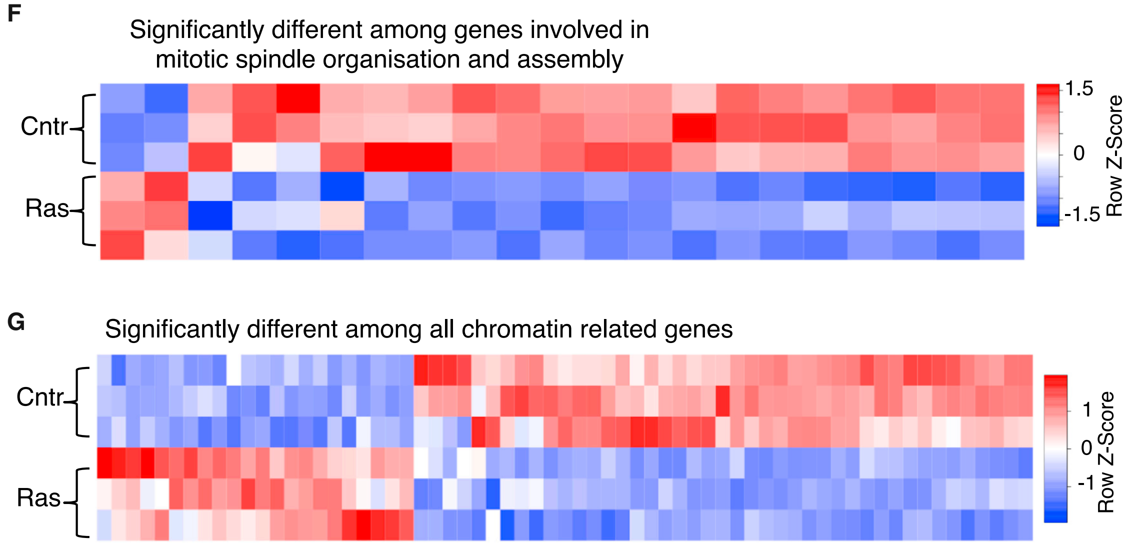


**Figure 3. Activated H-RasV12 Dysregulates a Subset of Mitotic Genes in Pre-senescent Cells**

(A) Experimental layout. 4-day-induced (+4OHT) or control (−4OHT) ERRAS cells were arrested in mitosis with DME (12–16 hr). Mitotic (detached) cells were selectively collected by a washout, and >90% mitotic index (MI) was confirmed by microscopic scoring. Total RNA was isolated and validated on the Bioanalyzer. A cDNA library was constructed from poly(A) RNA and subjected to RNA-seq. The experiment was independently repeated three times. See Table S1 for alignment statistics.

*(legend continued on next page)*

of mammalian target of rapamycin (mTOR) (with Everolimus), Nuclear Factor κB (NF-κB) (with parthenolide), or reactive oxygen species (ROS) (with N-acetylcysteine) had little effect on Mcl1 protein (Figure S3F). Furthermore, the high level of Mcl1 frequently overlapped with strong phospho-ERK1/2 staining in an in vivo mouse model of OIS in the pancreas, specifically in *KRAS*$^{G12D}$-triggered pancreatic intraepithelial neoplasias (mPanINs) that contain senescent-like cells (Caldwell et al., 2012; Collado and Serrano, 2010; Hingorani et al., 2003; Morton et al., 2010) (Figure 4H).

Since activated ERK is known to stabilize Mcl1 protein (Ding et al., 2008; Domina et al., 2004), we asked whether activated Ras increases Mcl1 protein stability. Surprisingly, we found no difference in the half-life of Mcl1 protein between induced and control ERRAS cells treated with the protein synthesis inhibitor cycloheximide (Figure 4I). On the other hand, the mRNA level of *MCL1* increased 3- to 4-fold upon Ras activation (Figures 4J and S3G) and was efficiently reduced by MEK inhibitor PD184352 (Figure 4K). Together, this suggests that oncogenic H-RasV12 upregulates Mcl1 in primary cells via ERK-dependent increase in mRNA abundance.

To test the requirement for a high level of Mcl1 in Ras-induced resistance to mitotic death, we depleted Mcl1 from control and induced ERRAS cells using small interfering RNA (siRNA) (Figure 5A). Tracking individual mitoses in time-lapse images revealed that depletion of Mcl1 reduced slippage out of DME-induced mitotic arrest and increased mitotic cell death (Figures 5B and 5C). Furthermore, ectopic expression of Mcl1 (Figure 5D) in control cells increased slippage and reduced death in DME-treated cells (Figures 5E and 5F), recapitulating the effect of Ras activation. Thus, in ERRAS cells, Ras-mediated upregulation of Mcl1 is necessary and sufficient for enhanced survival and increased slippage of damaged mitoses, contributing to generation of MNCs by activated H-RasV12.

## H-RasV12-Expressing Slipped Cells Are Senescent

To determine the fate of the Ras-induced slipped MNCs, we incubated purified mitotically arrested cells with DME for an additional 40 hr and collected and reseeded cells that slipped (reattached) in this period, as shown in Figure 6A. Following this protocol, approximately two thirds of the induced post-slippage ERRAS cells survived for at least 9 days (Figures 6B–6E). They were multinucleated (Figures 6C and S2E); proliferation arrested as measured by 5-ethynyl-2′-deoxyuridine (EdU) incorporation (Figure 6C); SA-β-gal-, p21-, and p16 positive and cyclin B1 negative (Figures 6D–6E and S3H); and displayed large flat cell morphology (data not shown), all of which indicating that they are senescent. siRNA-mediated knockdown of Mcl1, abundant in these cells (Figures S3H and S3I), moderately promoted cell death (Figure S3J). Thus, a large proportion of Ras-expressing slipped cells becomes multinucleate senescent cells.

## Mitotic Slippage and Oncogene Activation Cooperate in Establishing Senescence

Next, we asked if mitotic slippage can promote establishment of OIS. To visualize any contribution of mitotic slippage to OIS-associated cell-cycle arrest, we decreased expression of oncogenic H-RasV12 in induced ERRAS cells by titrating down tamoxifen (Figure 7A). Resulting low levels of H-RasV12 were unable to induce cell-cycle arrest (Figure 7B, 12.5 nM and 6.25 nM tamoxifen, no slippage). Likewise, slippage alone (protocol as in Figure 6A, −4OHT), failed to induce stable proliferation arrest characteristic of senescence, and instead caused only transient cell-cycle arrest in cells that survived DME treatment (Figures 7B, S4A, and S4B). Enlarged nuclear volume and lobulated nuclei or micronucleation in the proliferating EdU-positive cells confirmed that these cells had likely slipped out of DME-induced mitotic arrest (Figure S4C). In addition, these slipped cells displayed only weak SA-β-gal staining (Figure S4D, left panel) and little p16 (Figure S4E, left panels), underscoring that they are not senescent. However, combined low-level H-RasV12 expression and DME-enforced slippage effectively induced long-term cell-cycle arrest (Figure 7B, 12.5 nM and 6.25 nM 4OHT + slippage; Figure S4A). Thus, mitotic slippage synergizes with low-level oncogenic Ras in establishing senescence-associated long-term cell-cycle arrest.

To gain insight into potential molecular drivers of such synergy, we examined the effect of DME-induced slippage and low-level oncogenic H-RasV12 on key effectors of cellular senescence. We found that 2 days after slippage (7 days of Ras induction), p53 and its target p21, but not p16, were cooperatively upregulated by low-level H-RasV12 and mitotic slippage (Figure 7C; proportions of S-phase cells at this stage are shown in Figure S4F). However, 9 days after slippage (Figure 7B, 14 days of Ras induction) expression of p16 was, instead, cooperatively upregulated by slippage and activated Ras (Figure 7D).

P16 is induced in mouse neoplasia, where it correlates with OIS (Burd et al., 2013; Kuilman et al., 2010). Using p16-reporter mice, heterozygous transgenic mice expressing a luciferase reporter gene under control of the p16 promoter (p16$^{luc/wt}$) (Burd et al., 2013), we asked whether oncogene-induced p16 is altered by failed mitosis in vivo. Luminescence of p16 reporter was

(B) Total number of significantly upregulated genes (up) and downregulated genes (down) genes (5% FDR) in Ras-induced mitotic cells compared to control mitotic cells.
(C) PCA of Ras-induced (R1, R2, and R3) and control (C1, C2, and C3) mitoses based on expression of each known coding gene by FPKM. PC1 and PC2, principal components 1 and 2, respectively.
(D) Heatmap of significantly differentially expressed genes (5% FDR) within the mitotic-related gene set (collated from several GSAE/MSigDB entries, Broad Institute) between Ras-induced mitotic cells and control mitotic cells. Genes are represented in columns, and samples are represented in rows. In the FPKM-based column Z-score, intensity represents higher (red) to lower (blue) expression.
(E) DAVID GO analysis of differentially expressed genes between Ras-induced mitotic cells and control mitotic cells. The top ten most enriched ontologies are shown (FDR <5%).
(F) Heatmap of gene expression for genes within mitotic-spindle-related GO terms, derived and represented as in (D).
(G) Heatmap of gene expression for genes within the chromatin gene set derived and represented as in (D).
See also Tables S1 and S2.

OPEN ACCESS
CellPress

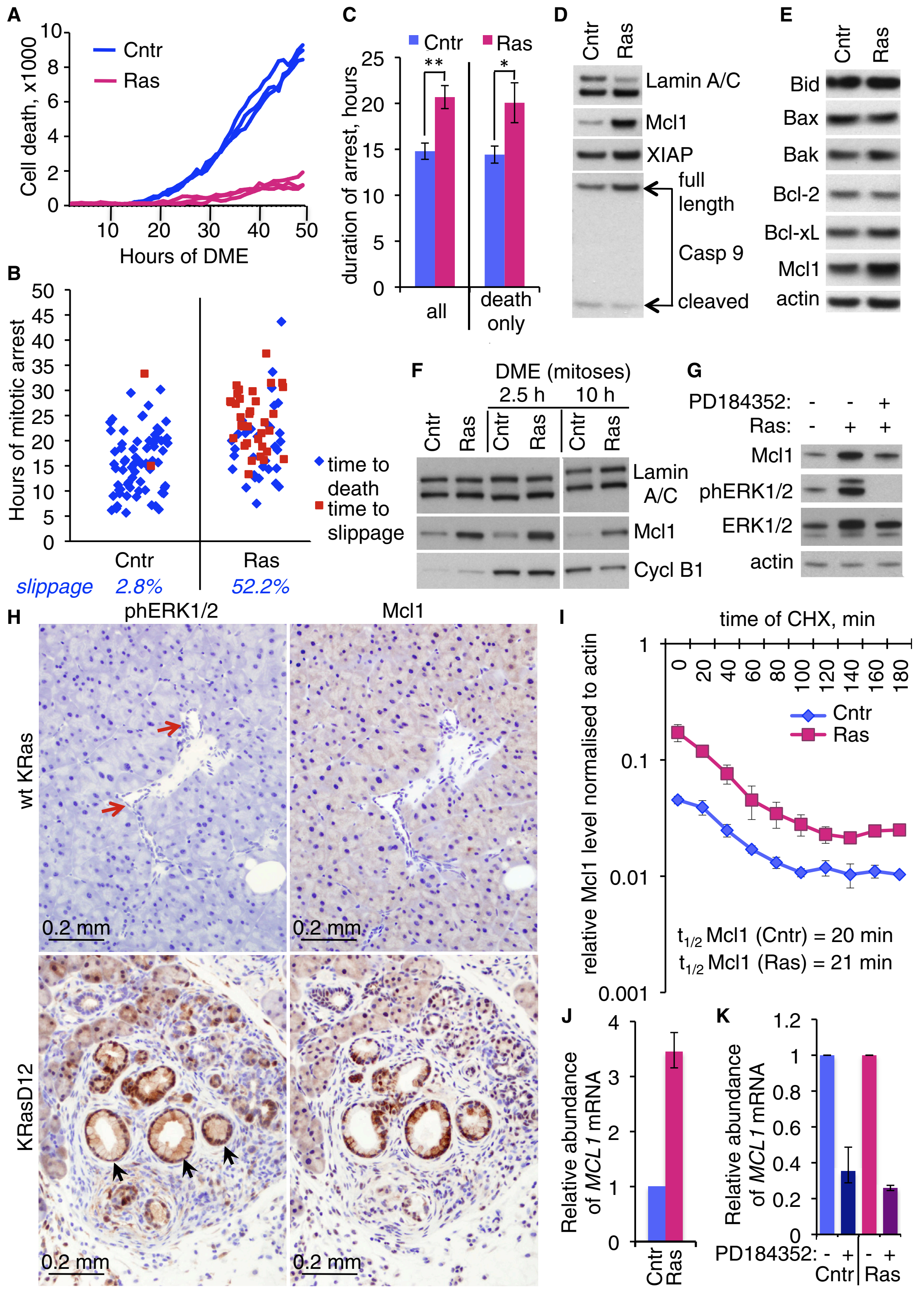


*(legend on next page)*

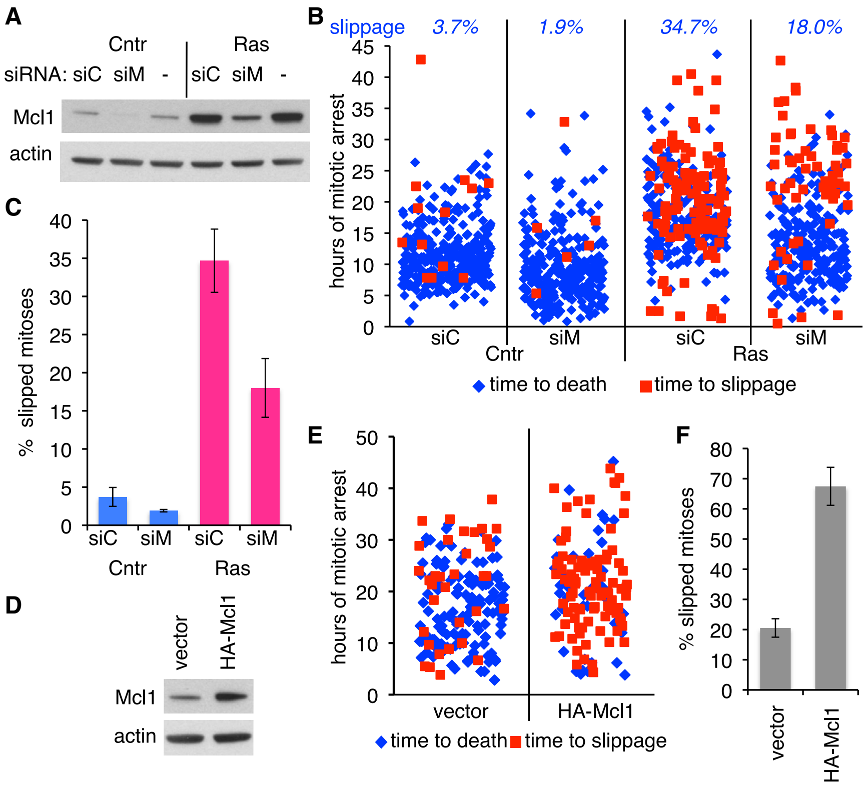


**Figure 5. Increased Mcl1 Is Responsible for H-RasV12-Enhanced Mitotic Slippage**

(A) Mcl1 depletion by siRNA in control (Cntr) or induced (Ras) ERRAS cells. siC, non-targeting siRNA; siM, Mcl1-targeting siRNA; -, no transfection. Actin is a loading control.

(B) Time-lapse analysis of duration and outcome of individual mitoses in control (Cntr) or 4-day-induced (Ras) ERRAS cells transfected with either Mcl1-targeting (siM) or non-targeting (siC) siRNA, treated with DME for 3 days. 207–337 mitoses per condition. Percentage of slippage is shown at top.

(C) Percentage of mitotic slippage quantified from (B). Data indicate mean ± SEM from three biological replicates, 63–115 mitoses each.

(D) Mcl1 level in ERRAS cells infected with retrovirus expressing HA-Mcl1 or vector only. Actin is a loading control.

(E) Time-lapse analysis of duration and outcome of individual mitoses in DME-treated HA-Mcl1 or vector-expressing uninduced ERRAS cells, 141–165 mitoses per condition.

(F) Percentage of mitotic slippage quantified from (E). Data indicate mean ± SEM from three replicates, 43–61 mitoses each.

activated at the site of a 10-day-old wound (Figure S5A), consistent with upregulation of p16 during wound healing (Burd et al., 2013; Demaria et al., 2014; Jun and Lau, 2010). To induce senescence associated with an activated Ras oncogene, we subjected mice to a DMBA/TPA skin carcinogenesis protocol. This treatment is associated with *H-RasQ61L* mutation, formation of skin papillomas containing senescent cells, and, ultimately, progression to skin carcinomas (Fujiki et al., 1989; Quintanilla et al., 1986; Sun et al., 2007). The DMBA-TPA protocol by itself led to induction of the p16 promoter at the site of treatment (Figures 7E and S5B). Mitotic defects were induced by intraperitoneal (i.p.) injection of SB-743921, an Eg5 inhibitor with the highest efficiency and the lowest toxicity in vivo (Rath and Kozielski, 2012) that has undergone a phase 1 clinical trial in humans (Holen et al., 2011). On its own, SB-743921 induced mitotic aberrations but had no significant effect on p16-promoter-driven luciferase activity (Figures 7E and S5C).

**Figure 4. Activated H-RasV12 Confers Resistance to Mitotic Death and Upregulates Mcl1**

(A) H-RasV12 protects from cell death caused by DME. Kinetics of cell death in DME-treated 4-day-induced (Ras, red) or control (Cntr, blue) ERRAS cells, measured by Sytox Green inclusion. The y axis shows numbers of dead cells recognized as fluorescent objects within four Incucyte images minus background. Triplicate measurements are shown.

(B) Enhanced mitotic slippage in cells with activated Ras. Control (Cntr) or 4-day-induced (Ras) ERRAS cells were imaged for 3 days under DME, and duration of mitotic arrest prior to cell death (blue dots) or prior to slippage (red dots) was quantified in randomly selected 71 (Cntr) or 67 (Ras) mitoses. Percentage of slippage is shown below. The experiment was repeated at least five times with similar outcome.

(C) Mean duration (±SEM) of DME-induced mitotic arrest in all cells (all, left) or leading to cell death (death only, right) in control (Cntr) or 3- to 5-day-induced (Ras) cells, quantified from four independent experiments. *p value = 0.025; **p value = 0.003 (paired Student's t test).

(D and E) Mcl1 protein level is increased in 4 days induced ERRAS (Ras), compared to control cells (Cntr). Cell lysates were blotted with panel of antibodies against apoptotic regulators and Lamin A/C or actin as loading controls.

(F) 4 days of induction increases Mcl1 in mitotically arrested ERRAS cells. Control (Cntr) or induced (Ras) ERRAS cells were treated with DME for 2.5 or 10 hr as indicated, collected by shake-off or left untreated (first two lanes), and immunoblotted for Mcl1 and Cyclin B1. Lamin A/C is a loading control.

(G) Reduction in Mcl1 protein level in 7-day-induced ERRAS upon ERK inhibition by 4 hr of PD184352. Simultaneous reduction in ERK1/2 phosphorylation at Thr202/Tyr204 is shown. Actin is a loading control.

(H) Colocalization of high-level Mcl1 and phospho-ERK in mouse pre-malignant mPanIN. Sections of pancreas from 4-month-old *PDX1-Cre* mice, wild-type (WT) for *KRas* (top) or heterozygous for $KRas^{G12D}$ (bottom), with pancreatic ducts (top, red arrows) or mPanINs (bottom, black arrows) stained for phospho-Thr202/Tyr204 ERK1/2 (left) or Mcl1 (right).

(I) Mcl1 protein stability is not altered by Ras induction. Relative amount of Mcl1 protein quantified from Mcl1/actin immunoblots from induced (Ras) or control (Cntr) ERRAS cells at indicated time in cyclohexamide (CHX). Data indicate mean ± SD from triplicate samples. The calculated half-lives of Mcl1 protein for both conditions are shown. (J) qPCR analysis of *MCL1* mRNA level in control (Cntr) and 4-day-induced (Ras) ERRAS cells. (K) Effect of 4 hr of PD184352 on *MCL1* mRNA level in control or 4-day-induced ERRAS cells measured by qPCR.

(J and K) Mean (±SD) relative abundance (with control set up as 1) of mRNA from three technical replicas, normalized to *GAPDH*.

See also Figures S2 and S3.

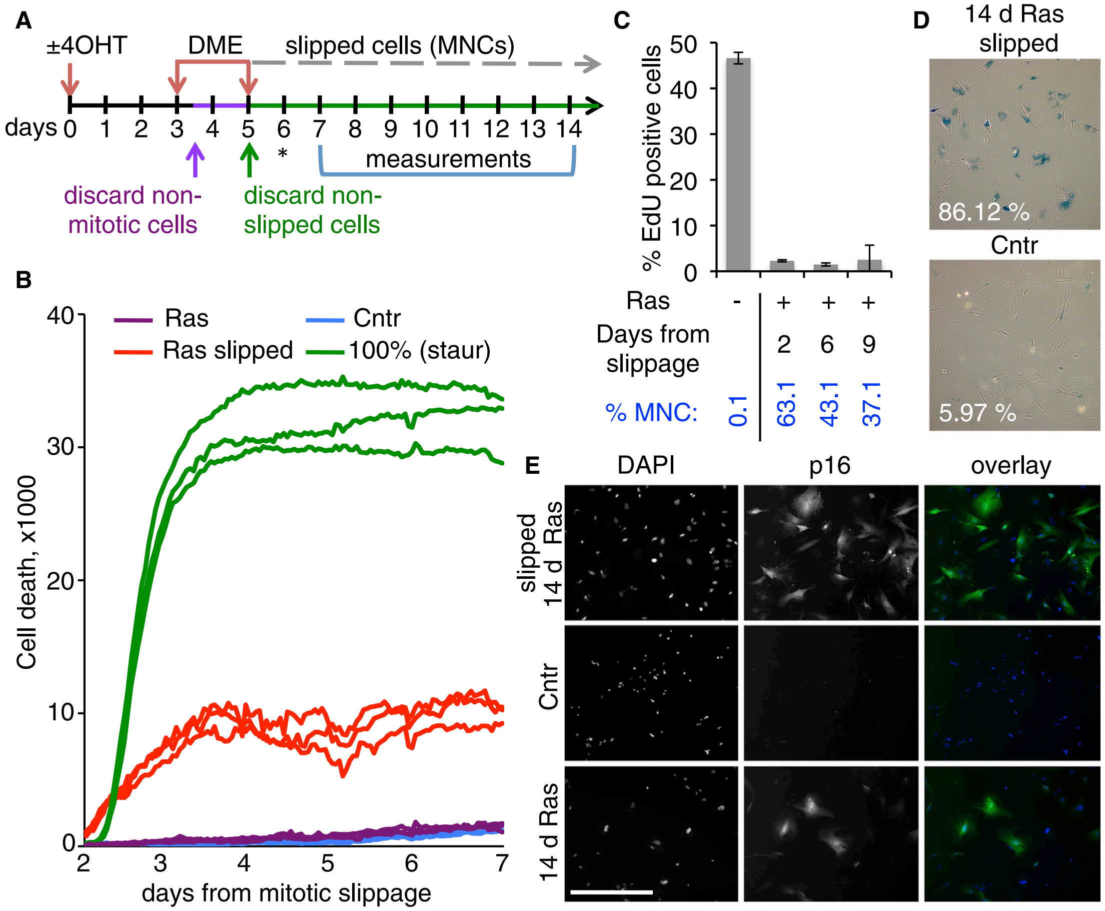


**Figure 6. Survival and Senescence of Cells with Activated H-RasV12 following Mitotic Slippage**

(A) Experimental layout. Control (−4OHT) or 3-day-induced ERRAS cells (+4OHT) were arrested with DME for 9 hr (control cells were seeded in excess to account for lower level of mitotic slippage). Mitoses were collected and re-plated under DME for a further 40 hr. After washing away the remaining mitoses and cell debris, the slipped (attached) control and induced cells were re-seeded at the same cellular density (time corresponding to 5 days after induction). Time of siRNA transfection for Figures S3I and S3J is indicated by an asterisk. Measurements were performed during period indicated by bracket.

(B) Cell death after slippage in induced ERRAS cells (Ras slipped), measured by incorporation of Sytox Green viability dye. Staurosporin treatment of control cells plated at the same initial density, 100% (staur), was used to visualize the maximal level of cell death in this assay (100% cell death was confirmed by visual examination). Untreated induced cells (Ras) as well as untreated control cells (Cntr) seeded at the same density are also shown. The data were acquired simultaneously with data in Figure S3J, which uses the Ras slipped and 100% (staur) data again as controls. Triplicate measurements are shown.

(C) Slippage induces durable cell-cycle arrest in induced ERRAS cells. Percentage of replicating cells measured by EdU incorporation at indicated times after slippage (mean ± SD from seven replicates). Percentages of multinucleate cells in the samples are given below (% MNC).

(D) SA-β-gal staining of 14-day-induced ERRAS cells 9 days after slippage (top), compared to untreated control ERRAS (bottom). Percentages of β-gal-positive cells are given. d, days.

(E) p16 immunofluorescence in 14-day-induced ERRAS cells 9 days after slippage (top). p16 staining in control (middle row) and 14-day-induced (senescent, bottom) ERRAS cells are shown as negative and positive controls, respectively. Scale bar, 200 μm. Overlay colors: DAPI, blue; p16, green.

See also Figure S3.

Remarkably, however, SB-743921 treatment together with DMBA-TPA induced a further significant increase in p16 activation (Figures 7E and 7F). This is consistent with our in vitro observation that mitotic disruption and an activated oncogene cooperate to upregulate p16.

## DISCUSSION

Here, we report that frequently observed multinucleate OIS cells stem in large part from specific oncogene-induced mitotic abnormalities, in conjunction with oncogene-induced upregulation of Mcl1 that promotes survival and slippage out of aberrant mitoses. Furthermore, we show that progression through mitotic slippage can enhance senescence induced by weak oncogenic signaling.

Previously, multinucleated senescent cells were suggested to be generated via endomitosis/cytokinesis failure (Leikam et al., 2008; Takahashi et al., 2006), cell-cell fusion (Leikam et al., 2008), and “amitosis” (fragmentation of polyploid nuclei in interphase) (Walen, 2006). Combining long-term time-lapse imaging

OPEN ACCESS
CellPress

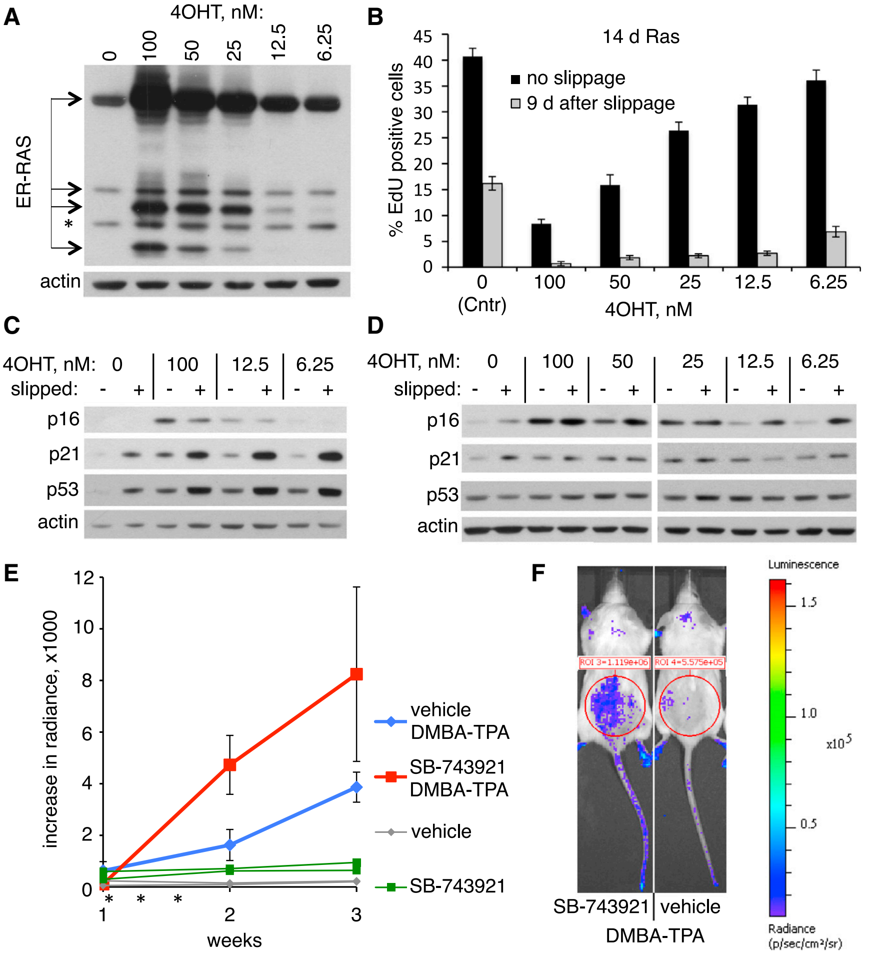


**Figure 7. Mitotic Slippage and Low-Level Oncogene Signaling Cooperate to Upregulate p21 and p16 and Induce Senescence-Associated Cell Cycle Arrest**

(A) Titration of ER-H-RasV12 level in ERRAS cells by differential 4OHT concentration. Lysates from ERRAS cells induced for 7 days with indicated 4OHT concentrations (0–100 nM) were blotted with anti-Ras antibody that recognizes both endogenous Ras (asterisk) and ER-RasV12 fusion. Actin is a loading control.

(B) Combination of mitotic slippage and low-level (subthreshold for OIS onset) H-RasV12 expression induces effective cell-cycle arrest in ERRAS cells. The experiment was conducted as in Figure 6A but using several concentrations of 4OHT. Percentage of replicating cells measured by EdU incorporation in control ERRAS cells (0, Cntr) or ERRAS cells treated with indicated concentrations of 4OHT are shown at 9 days after slippage (14 days of H-RasV12 induction). Data indicate means ± SD from seven replicate experiments. d, days.

(C and D) Immunoblots of the lysates from samples shown in (B) collected at 2 (C) or 9 (D) days after slippage, corresponding to 7 (C) or 14 (D) days of Ras induction, to detect changes in protein amounts of p53, p21, and p16. Actin is a loading control.

(E) Activation of p16-promoter-driven luciferase expression in $p16^{Luc/wt}$ transgenic mice, treated with a combination of ectopic DMBA-TPA protocol and Eg5 inhibitor SB-743921. Mice received DMBA (week 0, not shown) followed by TPA during weeks 1–3. Three i.p. injections of SB-743921 or vehicle were given during the first week of TPA (shown as asterisks). Luciferase activity was measured weekly using the IVIS in vivo imaging system. Average increases in luciferase activity (as compared to week 0) ± SEM in vehicle-treated cohort (n = 8, blue) or in SB-743921-treated cohort (n = 9, red) are shown. Difference between cohorts is significant at week 2, with $p < 0.05$. Changes in luciferase expression in two individual vehicle-treated (gray) and two individual SB-743921-treated (green) mice without DMBA-TPA application are shown as controls.

(F) Example of p16-promoter-driven luciferase expression activated by the DMBA-TPA protocol (third week) with (left) or without (right) SB-743921 injections in $p16^{Luc/wt}$ mice, as measured by luminescence in the presence of D-licuferin substrate. The color key to radiance of luminescence is on the right. The measurement regions (cycles) corresponding to the areas subjected to DMBA/TPA applications are shown.

See also Figures S4 and S5.

with fluorescent labeling of nuclei in cells undergoing OIS, we achieved a superior resolution that allowed an unbiased tracking of any detected multinucleated cells back to their origin. This unequivocally showed that multinucleation is predominantly due to mitotic failure. We further describe a spontaneous mitotic arrest and slippage in the course of OIS that generates senescent cells with multiple nuclei, a process not found in control primary cells.

Remarkably, we found that H-RasV12 expression in primary cells induces a range of mitotic defects that are well reflected in accompanying changes in gene expression, specifically in mitotic spindle and chromatin regulatory genes. We also established that activated Ras alters the fate of aberrant mitoses, from predominant cell death to frequent mitotic slippage. We determined that this depends on ERK-mediated increase in the level of anti-apoptotic protein Mcl1, in line with an established role of Mcl1 in mitosis (Harley et al., 2010; Topham and Taylor, 2013). Based on the aforementioned findings, we suggest that multinucleation in OIS results from oncogene-induced dysregulation of mitotic genes combined with Mcl1-dependent resistance to mitotic death, causing cells with excessive mitotic defects to exit mitosis via slippage, ultimately forming senescent MNCs. Although such multinucleation is a relatively rare event, genome aberrations associated with multinucleation can be a source of genome instability, which is, in turn, a contributor to malignancy (Fox and Duronio, 2013). Indeed, a recent study showed that multinucleate senescent melanocytes could give rise to tumor-initiating cells (Leikam et al., 2015). This is important, given that approximately 25% of melanomas are thought to arise in association with a pre-existing nevus (Smolle et al., 1999; Stolz et al., 1989)

Our data implicate aberrant mitosis in the establishment of OIS. MNCs are likely to be an extreme manifestation of the more widespread defects associated with mitotic aberrations ("mitotic stress") during transition to senescence (compare Figures 1C and 2B). Aberrant mitotic progression was shown to

generate de novo DNA damage (Colin et al., 2015; Ganem and Pellman, 2012; Hayashi and Karlseder, 2013). Considering that DNA damage response is a major driver of OIS (Di Micco et al., 2006), it is conceivable that damage associated with Ras-induced mitotic stress could also contribute to the establishment of senescence, in the same way that Ras-induced DNA replication stress triggers senescence (Di Micco et al., 2006). Indeed, we show that mitotic stress has the ability to potentiate pro-senescence oncogenic signaling. Our data suggest that p21 and, later, p16 are cooperatively upregulated by aberrant mitotic progression and sub-threshold H-RasV12, and, thus, it is possible that mitotic stress and oncogenic signaling cooperate to reach the critical level of senescence mediators.

In summary, our work has delineated a cellular process underlying multinucleation in OIS that involves impaired mitosis combined with increased Mcl1-dependent survival and subsequent slippage from aberrant mitosis, followed by a cell-cycle arrest. We propose that oncogene-induced mitotic stress cooperates acutely with other senescence effector pathways to induce OIS. However, in the long term, such multinucleate senescent cells may carry increased risk of malignant progression.

## EXPERIMENTAL PROCEDURES

### Cells

Primary human fibroblasts IMR90 and BJ cells were retrovirally transduced with ER-H-RasV12-encoding pLNC-Ras:ER (Barradas et al., 2009) to generate ERRAS cells. ER-H-RasV12 was activated by continuous treatment with 100 ng/ml (unless indicated otherwise) 4-hydroxytamoxifen (4OHT). Hemagglutinin epitope tag (HA) containing pLZRS-HA-Mcl1, pLZRS control vector, GFP-fused Lamin A, or GFP alone was introduced to ERRAS cells or parental IMR90 cells via retroviral constructs. See the Supplemental Experimental Procedures for details. Cells were arrested in mitosis with 1 μM Eg5 inhibitor III (DME, Calbiochem/Merck, catalog number 324622) for indicated time and harvested by shake-off. For mitotic slippage, mitoses were further incubated with DME for up to 3 days, and any unattached cells and debris were washed away before attached (slipped) cells were harvested.

### siRNA

Mcl1 depletion was performed with siGENOME SMARTpool Mcl1-targeting siRNA, while non-targeting siRNA was used as a control (see details in the Supplemental Experimental Procedures).

### Cell Death/Apoptosis Assays

Apoptosis was detected by fluorescence of the NucView 488 caspase-3 substrate (Biotium) and quantified by flow cytometry (see details in the Supplemental Experimental Procedures). When required, it was combined with the identification of mitotic cells (by phospho-histone H3 staining). Alternatively, cells plated at identical densities were incubated in the presence of Sytox Green viability dye (Invitrogen #S7020), and kinetics of Sytox Green incorporation (reflecting cell death) was imaged, measured, and analyzed using the IncuCyte FLR imaging system (Essen Bioscience). Each data point represents the number of dead cells (automatically recognized as fluorescent objects) within four image acquisition windows after subtracting the background when necessary.

### Measurement of DNA Synthesis

BrdU incorporation after a 5-hr BrdU pulse was measured as described in the Supplemental Experimental Procedures. Alternatively, cells on 96-well plates were pulsed with EdU for 3 hr, followed by EdU detection using the Click-IT EdU imaging kit (Life Technologies). Plates were scanned with the Operetta High Content Imaging System (PerkinElmer) and analyzed as described in the Supplemental Experimental Procedures.

### Immunofluorescence

Cells cultured on glass coverslips were fixed by the appropriate method, immunostained as described in the Supplemental Experimental Procedures, and counterstained with DAPI. Image acquisition, processing, and analysis are detailed in the Supplemental Experimental Procedures.

### SA-β-gal Staining

Cells on coverslips were fixed with PBS containing 2% formaldehyde and 0.2% glutaraldehyde for 5 min, washed in PBS, and stained overnight at 37°C in staining solution containing 150 mM NaCl, 2 mM $MgCl_2$, 5 mM $K_3Fe(CN)_6$, 5 mM $K_4Fe(CN)_6$, 40 mM $Na_2HPO_4$, and 1 mg/ml X-Gal (Sigma Aldrich #16664).

### Immunoblotting

Cellular lysates were prepared and analyzed on SDS-PAGE as described in the Supplemental Experimental Procedures. Protein stability was determined from the time-course measurements of Mcl1 and actin levels in immunoblots of cells treated with 10 μg/ml cycloheximide (Sigma) in triplicates, as described in the Supplemental Experimental Procedures.

### PCR and qPCR

Total RNA was extracted with the RNeasy Plus Mini Kit (QIAGEN), followed by DNase I treatment. cDNA was produced using oligo-dT primers. Semi-qPCR was performed with Mcl1 and APRT primers and visualized as detailed in the Supplemental Experimental Procedures. Real-time PCR was performed using separately designed *MCL1L*-specific primers (see Supplemental Experimental Procedures) with the SYBR-Green PCR Master Mix (Life Technologies) on the BioRad Chromo4 thermo cycler. Triplicate C(t) data for *MCL1* and a housekeeping gene (*GAPDH*) were analyzed using the REST program to calculate changes in gene expression.

### RNA-seq and Data Analysis

Total RNA isolated from mitotically arrested or unsynchronized cells was used to generate the cDNA library. Samples were sequenced on an Illumina NextSeq500. Paired-end reads were aligned to the human genome (hg19) using the splicing-aware aligner Tophat2. Reference splice junctions were provided by a reference transcriptome (Ensembl build 73). FPKM (fragments per kilobase million) values were calculated using Cufflinks. Differential gene expression was determined using the cuffdiff maximum likelihood estimate function. Genes of significantly changing expression were defined as FDR-corrected p value $\leq$ 0.05. See details in the Supplemental Experimental Procedures. RNA-seq data are available at GSE70668.

Histopathology, immunohistochemistry/tissue immunofluorescence, and details of animal studies are described in the Supplemental Experimental Procedures.

All animal work was carried out according to UK Home Office regulations, in line with the European Directive 2010 and approved by ethical review (University of Glasgow).

## ACCESSION NUMBERS

The RNA-seq data reported in this paper have been deposited to the NCBI GEO and are available under accession number GEO: GSE70668.

## SUPPLEMENTAL INFORMATION

Supplemental Information includes Supplemental Experimental Procedures, five figures, two tables, and seven movies and can be found with this article online at http://dx.doi.org/10.1016/j.celrep.2015.07.055.

## ACKNOWLEDGMENTS

We thank Margaret O'Prey and Tom Gilbey from Beatson Advanced Imaging Resource, David Strachan for advice on data analysis, Profs. Norman E. Sharpless and David H. Beach for p16 reporter mice, and all Beatson BSU staff. Animal experiments in the BSU were supported in part by Cancer Research UK

Glasgow Centre (C596/A18076) and the BSU facilities at the Cancer Research UK Beatson Institute (C596/A17196). The study was funded by CRUK program grant C10652/A16566 and MRC project grant BB/K017223/1 to P.D.A. Thanks to all members of the P.D.A. lab for critical discussions.

Received: September 2, 2014
Revised: June 22, 2015
Accepted: July 27, 2015
Published: August 20, 2015

## REFERENCES

Barradas, M., Anderton, E., Acosta, J.C., Li, S., Banito, A., Rodriguez-Niedenführ, M., Maertens, G., Banck, M., Zhou, M.M., Walsh, M.J., et al. (2009). Histone demethylase JMJD3 contributes to epigenetic control of INK4a/ARF by oncogenic RAS. Genes Dev. *23*, 1177–1182.

Berlingeri-Ramos, A.C., Morales-Burgos, A., Sánchez, J.L., and Nogales, E.M. (2010). Spitz nevus in a Hispanic population: a clinicopathological study of 130 cases. Am. J. Dermatopathol. *32*, 267–275.

Braig, M., Lee, S., Loddenkemper, C., Rudolph, C., Peters, A.H., Schlegelberger, B., Stein, H., Dörken, B., Jenuwein, T., and Schmitt, C.A. (2005). Oncogene-induced senescence as an initial barrier in lymphoma development. Nature *436*, 660–665.

Brito, D.A., and Rieder, C.L. (2006). Mitotic checkpoint slippage in humans occurs via cyclin B destruction in the presence of an active checkpoint. Curr. Biol. *16*, 1194–1200.

Burd, C.E., Sorrentino, J.A., Clark, K.S., Darr, D.B., Krishnamurthy, J., Deal, A.M., Bardeesy, N., Castrillon, D.H., Beach, D.H., and Sharpless, N.E. (2013). Monitoring tumorigenesis and senescence in vivo with a p16(INK4a)-luciferase model. Cell *152*, 340–351.

Caldwell, M.E., DeNicola, G.M., Martins, C.P., Jacobetz, M.A., Maitra, A., Hruban, R.H., and Tuveson, D.A. (2012). Cellular features of senescence during the evolution of human and murine ductal pancreatic cancer. Oncogene *31*, 1599–1608.

Campisi, J., and d'Adda di Fagagna, F. (2007). Cellular senescence: when bad things happen to good cells. Nat. Rev. Mol. Cell Biol. *8*, 729–740.

Chen, Z., Trotman, L.C., Shaffer, D., Lin, H.K., Dotan, Z.A., Niki, M., Koutcher, J.A., Scher, H.I., Ludwig, T., Gerald, W., et al. (2005). Crucial role of p53-dependent cellular senescence in suppression of Pten-deficient tumorigenesis. Nature *436*, 725–730.

Cho, R.J., Huang, M., Campbell, M.J., Dong, H., Steinmetz, L., Sapinoso, L., Hampton, G., Elledge, S.J., Davis, R.W., and Lockhart, D.J. (2001). Transcriptional regulation and function during the human cell cycle. Nat. Genet. *27*, 48–54.

Colin, D.J., Hain, K.O., Allan, L.A., and Clarke, P.R. (2015). Cellular responses to a prolonged delay in mitosis are determined by a DNA damage response controlled by Bcl-2 family proteins. Open Biol. *5*, 140156.

Collado, M., and Serrano, M. (2010). Senescence in tumours: evidence from mice and humans. Nat. Rev. Cancer *10*, 51–57.

Collado, M., Gil, J., Efeyan, A., Guerra, C., Schuhmacher, A.J., Barradas, M., Benguría, A., Zaballos, A., Flores, J.M., Barbacid, M., et al. (2005). Tumour biology: senescence in premalignant tumours. Nature *436*, 642.

Demaria, M., Ohtani, N., Youssef, S.A., Rodier, F., Toussaint, W., Mitchell, J.R., Laberge, R.M., Vijg, J., Van Steeg, H., Dollé, M.E., et al. (2014). An essential role for senescent cells in optimal wound healing through secretion of PDGF-AA. Dev. Cell *31*, 722–733.

Di Micco, R., Fumagalli, M., Cicalese, A., Piccinin, S., Gasparini, P., Luise, C., Schurra, C., Garre', M., Nuciforo, P.G., Bensimon, A., et al. (2006). Oncogene-induced senescence is a DNA damage response triggered by DNA hyper-replication. Nature *444*, 638–642.

Dimri, G.P., Lee, X., Basile, G., Acosta, M., Scott, G., Roskelley, C., Medrano, E.E., Linskens, M., Rubelj, I., Pereira-Smith, O., et al. (1995). A biomarker that identifies senescent human cells in culture and in aging skin in vivo. Proc. Natl. Acad. Sci. USA *92*, 9363–9367.

Ding, Q., Huo, L., Yang, J.Y., Xia, W., Wei, Y., Liao, Y., Chang, C.J., Yang, Y., Lai, C.C., Lee, D.F., et al. (2008). Down-regulation of myeloid cell leukemia-1 through inhibiting Erk/Pin 1 pathway by sorafenib facilitates chemosensitization in breast cancer. Cancer Res. *68*, 6109–6117.

Domina, A.M., Vrana, J.A., Gregory, M.A., Hann, S.R., and Craig, R.W. (2004). MCL1 is phosphorylated in the PEST region and stabilized upon ERK activation in viable cells, and at additional sites with cytotoxic okadaic acid or taxol. Oncogene *23*, 5301–5315.

Fox, D.T., and Duronio, R.J. (2013). Endoreplication and polyploidy: insights into development and disease. Development *140*, 3–12.

Fujiki, H., Suganuma, M., Yoshizawa, S., Kanazawa, H., Sugimura, T., Manam, S., Kahn, S.M., Jiang, W., Hoshina, S., and Weinstein, I.B. (1989). Codon 61 mutations in the c-Harvey-ras gene in mouse skin tumors induced by 7,12-dimethylbenz[a]anthracene plus okadaic acid class tumor promoters. Mol. Carcinog. *2*, 184–187.

Ganem, N.J., and Pellman, D. (2012). Linking abnormal mitosis to the acquisition of DNA damage. J. Cell Biol. *199*, 871–881.

Gray-Schopfer, V.C., Cheong, S.C., Chong, H., Chow, J., Moss, T., Abdel-Malek, Z.A., Marais, R., Wynford-Thomas, D., and Bennett, D.C. (2006). Cellular senescence in naevi and immortalisation in melanoma: a role for p16? Br. J. Cancer *95*, 496–505.

Harley, M.E., Allan, L.A., Sanderson, H.S., and Clarke, P.R. (2010). Phosphorylation of Mcl-1 by CDK1-cyclin B1 initiates its Cdc20-dependent destruction during mitotic arrest. EMBO J. *29*, 2407–2420.

Hayashi, M.T., and Karlseder, J. (2013). DNA damage associated with mitosis and cytokinesis failure. Oncogene *32*, 4593–4601.

Hingorani, S.R., Petricoin, E.F., Maitra, A., Rajapakse, V., King, C., Jacobetz, M.A., Ross, S., Conrads, T.P., Veenstra, T.D., Hitt, B.A., et al. (2003). Preinvasive and invasive ductal pancreatic cancer and its early detection in the mouse. Cancer Cell *4*, 437–450.

Holen, K.D., Belani, C.P., Wilding, G., Ramalingam, S., Volkman, J.L., Ramanathan, R.K., Vasist, L.S., Bowen, C.J., Hodge, J.P., Dar, M.M., and Ho, P.T. (2011). A first in human study of SB-743921, a kinesin spindle protein inhibitor, to determine pharmacokinetics, biologic effects and establish a recommended phase II dose. Cancer Chemother. Pharmacol. *67*, 447–454.

Hübner, S., Eam, J.E., Wagstaff, K.M., and Jans, D.A. (2006). Quantitative analysis of localization and nuclear aggregate formation induced by GFP-lamin A mutant proteins in living HeLa cells. J. Cell. Biochem. *98*, 810–826.

Johmura, Y., Shimada, M., Misaki, T., Naiki-Ito, A., Miyoshi, H., Motoyama, N., Ohtani, N., Hara, E., Nakamura, M., Morita, A., et al. (2014). Necessary and sufficient role for a mitosis skip in senescence induction. Mol. Cell *55*, 73–84.

Jun, J.I., and Lau, L.F. (2010). The matricellular protein CCN1 induces fibroblast senescence and restricts fibrosis in cutaneous wound healing. Nat. Cell Biol. *12*, 676–685.

Krenning, L., Feringa, F.M., Shaltiel, I.A., van den Berg, J., and Medema, R.H. (2014). Transient activation of p53 in G2 phase is sufficient to induce senescence. Mol. Cell *55*, 59–72.

Kuilman, T., Michaloglou, C., Mooi, W.J., and Peeper, D.S. (2010). The essence of senescence. Genes Dev. *24*, 2463–2479.

Leikam, C., Hufnagel, A., Schartl, M., and Meierjohann, S. (2008). Oncogene activation in melanocytes links reactive oxygen to multinucleated phenotype and senescence. Oncogene *27*, 7070–7082.

Leikam, C., Hufnagel, A.L., Otto, C., Murphy, D.J., Mühling, B., Kneitz, S., Nanda, I., Schmid, M., Wagner, T.U., Haferkamp, S., et al. (2015). In vitro evidence for senescent multinucleated melanocytes as a source for tumor-initiating cells. Cell Death Dis. *6*, e1711.

Leopold, J.G., and Richards, D.B. (1967). Cellular blue naevi. J. Pathol. Bacteriol. *94*, 247–255.

Lin, A.W., Barradas, M., Stone, J.C., van Aelst, L., Serrano, M., and Lowe, S.W. (1998). Premature senescence involving p53 and p16 is activated in response to constitutive MEK/MAPK mitogenic signaling. Genes Dev. *12*, 3008–3019.

Liu, Z., Li, H., Derouet, M., Filmus, J., LaCasse, E.C., Korneluk, R.G., Kerbel, R.S., and Rosen, K.V. (2005). ras Oncogene triggers up-regulation of cIAP2

and XIAP in intestinal epithelial cells: epidermal growth factor receptor-dependent and -independent mechanisms of ras-induced transformation. J. Biol. Chem. *280*, 37383–37392.

Mao, Z., Ke, Z., Gorbunova, V., and Seluanov, A. (2012). Replicatively senescent cells are arrested in G1 and G2 phases. Aging (Albany, N.Y.) *4*, 431–435.

Michaloglou, C., Vredeveld, L.C., Soengas, M.S., Denoyelle, C., Kuilman, T., van der Horst, C.M., Majoor, D.M., Shay, J.W., Mooi, W.J., and Peeper, D.S. (2005). BRAFE600-associated senescence-like cell cycle arrest of human naevi. Nature *436*, 720–724.

Morton, J.P., Timpson, P., Karim, S.A., Ridgway, R.A., Athineos, D., Doyle, B., Jamieson, N.B., Oien, K.A., Lowy, A.M., Brunton, V.G., et al. (2010). Mutant p53 drives metastasis and overcomes growth arrest/senescence in pancreatic cancer. Proc. Natl. Acad. Sci. USA *107*, 246–251.

Narita, M., Nũnez, S., Heard, E., Narita, M., Lin, A.W., Hearn, S.A., Spector, D.L., Hannon, G.J., and Lowe, S.W. (2003). Rb-mediated heterochromatin formation and silencing of E2F target genes during cellular senescence. Cell *113*, 703–716.

Patino, W.D., Hutchens, K.A., Kapil, J., Chiou, Y., and Gottlieb, G.J. (2012). Eosinophilic cytoplasmic inclusion bodies in vesicular multinucleated melanocytes: a clue to the diagnosis of benign melanocytic lesions. Am. J. Dermatopathol. *34*, 424–427.

Quintanilla, M., Brown, K., Ramsden, M., and Balmain, A. (1986). Carcinogen-specific mutation and amplification of Ha-ras during mouse skin carcinogenesis. Nature *322*, 78–80.

Rath, O., and Kozielski, F. (2012). Kinesins and cancer. Nat. Rev. Cancer *12*, 527–539.

Reuter, J.A., and Khavari, P.A. (2006). Use of conditionally active ras fusion proteins to study epidermal growth, differentiation, and neoplasia. Methods Enzymol. *407*, 691–702.

Rieder, C.L., and Maiato, H. (2004). Stuck in division or passing through: what happens when cells cannot satisfy the spindle assembly checkpoint. Dev. Cell *7*, 637–651.

Salama, R., Sadaie, M., Hoare, M., and Narita, M. (2014). Cellular senescence and its effector programs. Genes Dev. *28*, 99–114.

Savchenko, IuI.u. (1988). [Endomitosis in pigmented neoplasms of human skin]. Tsitol. Genet. *22*, 20–24.

Smolle, J., Kaddu, S., and Kerl, H. (1999). Non-random spatial association of melanoma and naevi–a morphometric analysis. Melanoma Res. *9*, 407–412.

Stolz, W., Schmoeckel, C., Landthaler, M., and Braun-Falco, O. (1989). Association of early malignant melanoma with nevocytic nevi. Cancer *63*, 550–555.

Sun, P., Yoshizuka, N., New, L., Moser, B.A., Li, Y., Liao, R., Xie, C., Chen, J., Deng, Q., Yamout, M., et al. (2007). PRAK is essential for ras-induced senescence and tumor suppression. Cell *128*, 295–308.

Takahashi, A., Ohtani, N., Yamakoshi, K., Iida, S., Tahara, H., Nakayama, K., Nakayama, K.I., Ide, T., Saya, H., and Hara, E. (2006). Mitogenic signalling and the p16INK4a-Rb pathway cooperate to enforce irreversible cellular senescence. Nat. Cell Biol. *8*, 1291–1297.

Topham, C.H., and Taylor, S.S. (2013). Mitosis and apoptosis: how is the balance set? Curr. Opin. Cell Biol. *25*, 780–785.

Vergel, M., Marin, J.J., Estevez, P., and Carnero, A. (2010). Cellular senescence as a target in cancer control. J. Aging Res. *2011*, 725365.

Walen, K.H. (2006). Human diploid fibroblast cells in senescence; cycling through polyploidy to mitotic cells. In Vitro Cell. Dev. Biol. Anim. *42*, 216–224.

Young, A.R., Narita, M., Ferreira, M., Kirschner, K., Sadaie, M., Darot, J.F., Tavaré, S., Arakawa, S., Shimizu, S., Watt, F.M., and Narita, M. (2009). Autophagy mediates the mitotic senescence transition. Genes Dev. *23*, 798–803.

Zhu, Y., Zhou, Y., and Shi, J. (2014). Post-slippage multinucleation renders cytotoxic variation in anti-mitotic drugs that target the microtubules or mitotic spindle. Cell Cycle *13*, 1756–1764.

**Cell Reports**

**Supplemental Information**

# Mitotic Stress Is an Integral Part of the Oncogene-Induced Senescence Program that Promotes Multinucleation and Cell Cycle Arrest

**Dina Dikovskaya, John J. Cole, Susan M. Mason, Colin Nixon, Saadia A. Karim, Lynn McGarry, William Clark, Rachael N. Hewitt, Morgan A. Sammons, Jiajun Zhu, Dimitris Athineos, Joshua D.G. Leach, Francesco Marchesi, John van Tuyn, Stephen W. Tait, Claire Brock, Jennifer P. Morton, Hong Wu, Shelley L. Berger, Karen Blyth, and Peter D. Adams**

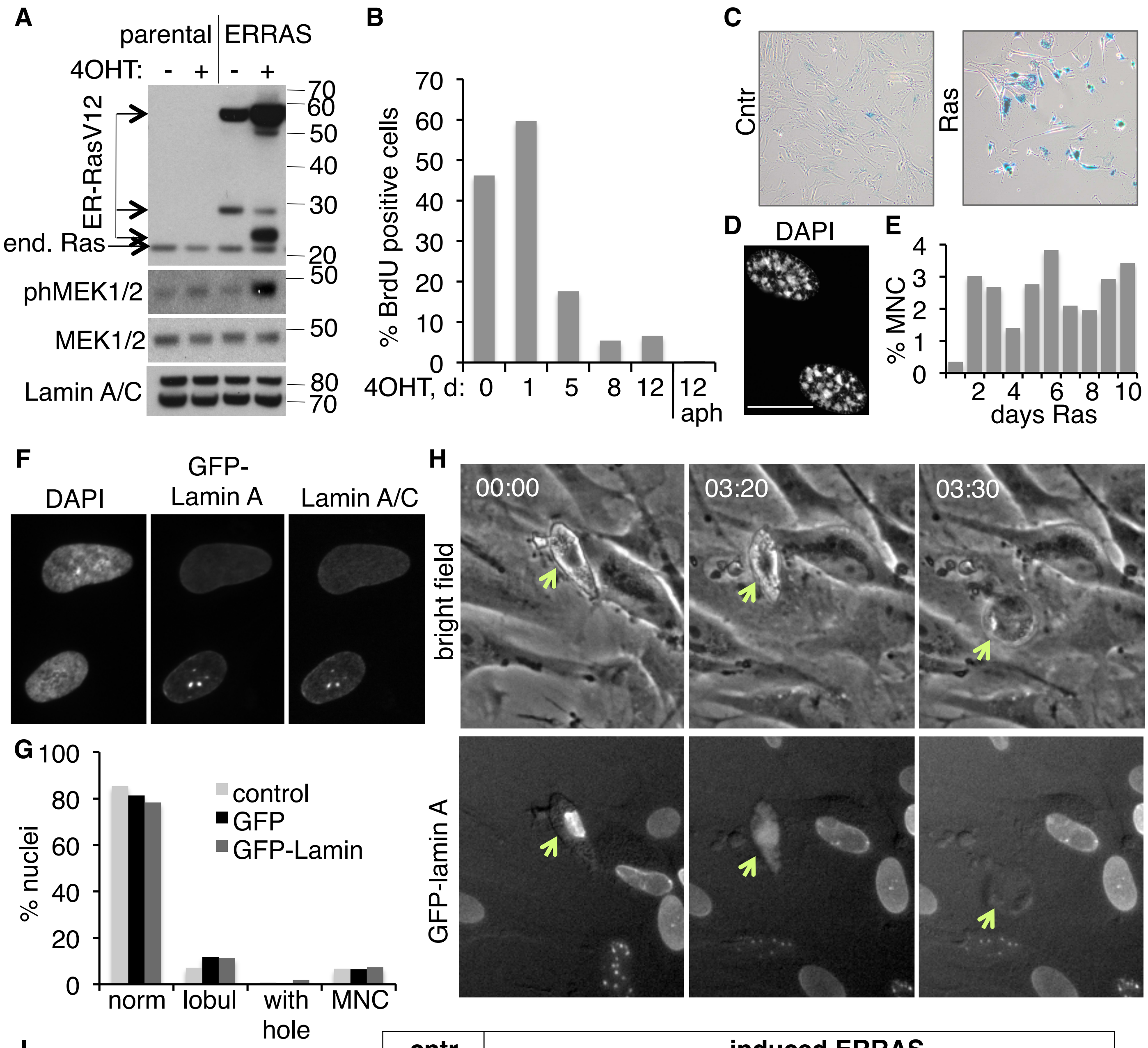


I

| | cntr ERRAS | induced ERRAS | | | | |
|---|---|---|---|---|---|---|
| | | 0-2 days | 2-4 days | 4-7 days | 9-13 days | total (ind) |
| cell fusion | 0 | 0 | 2 | 0 | 1 | **3** |
| slippage from mitotic arrest | 0 | 2 | 4 | 5 | 0 | **11** |
| cytokinesis failure | 6 | 2 | 1 | 3 | 1 | **7** |
| fragmentation of nucleus | 1 | 0 | 2 | 0 | 0 | **2** |
| **total multinucleations** | **7** | **4** | **9** | **8** | **2** | **23** |
| time-lapses tracked | 16 | 12 | 20 | 15 | 6 | 53 |
| number of cell divisions per time-lapse* | 49.3 ±12.2 | 85.3 ±25.6 | 41 ±23.2 | 20.7 ±11.4 | 0.3 ±0.8 | |

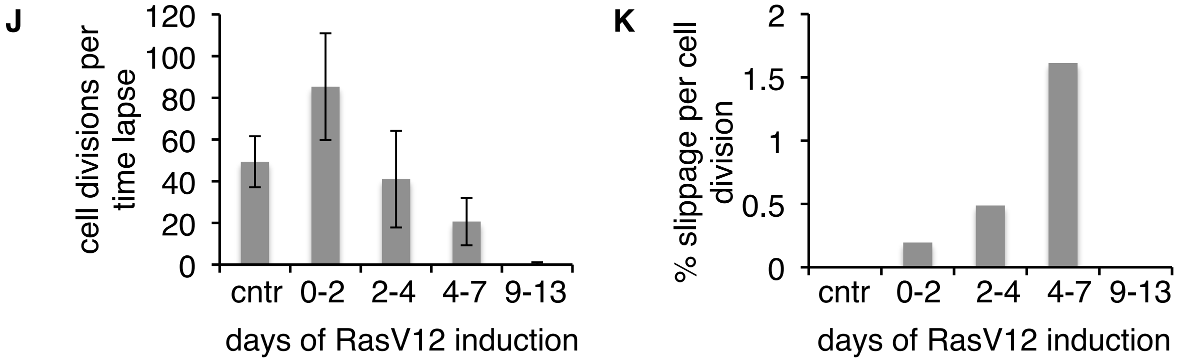


Figure S1

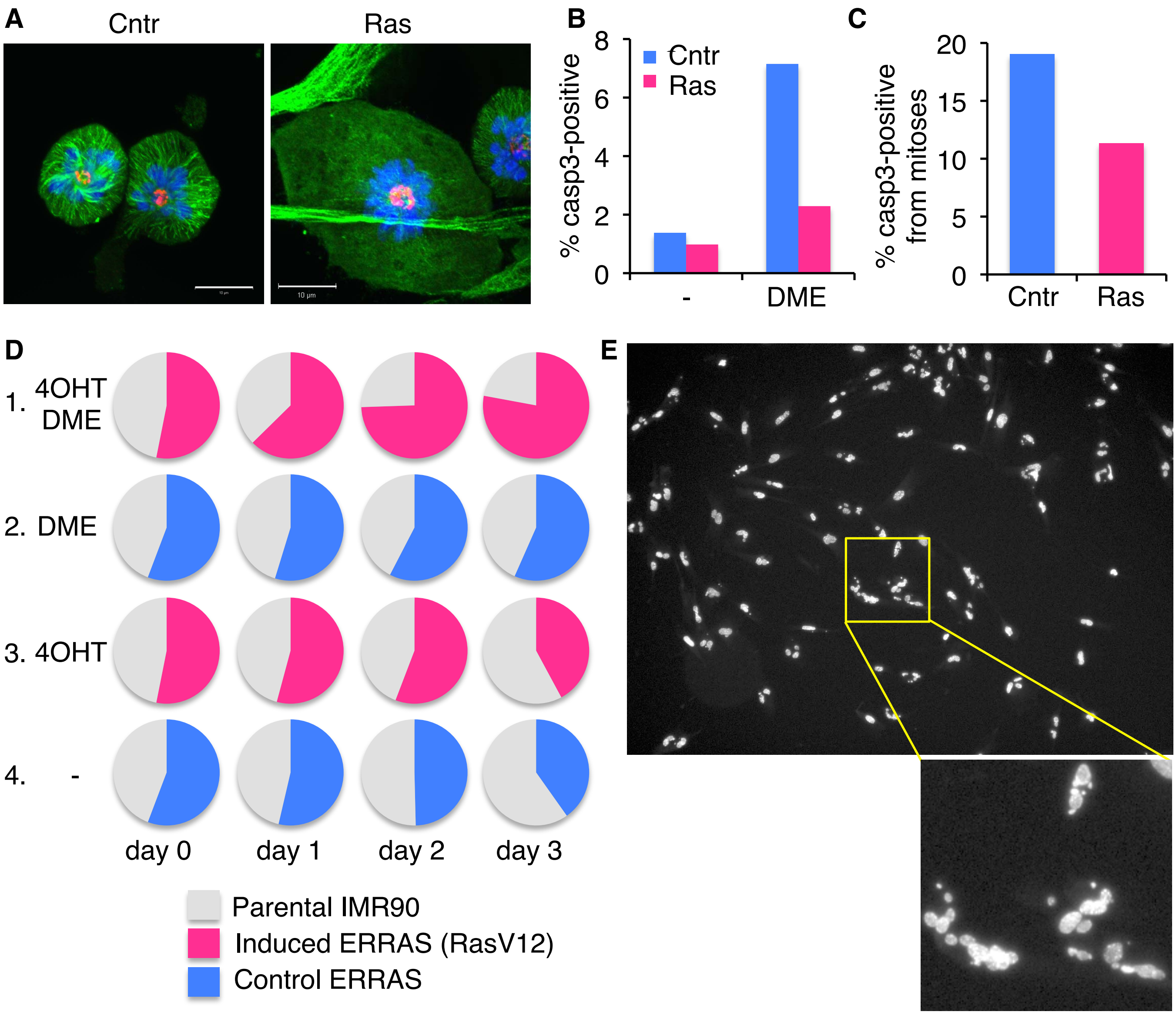

A
Cntr
Ras
10 μm
B
8
6
4
2
0
% casp3-positive
Cntr
Ras
-
DME
C
20
15
10
5
0
% casp3-positive from mitoses
Cntr
Ras
D
1. 4OHT DME
2. DME
3. 4OHT
4. -
day 0
day 1
day 2
day 3
Parental IMR90
Induced ERRAS (RasV12)
Control ERRAS
E


Figure S2

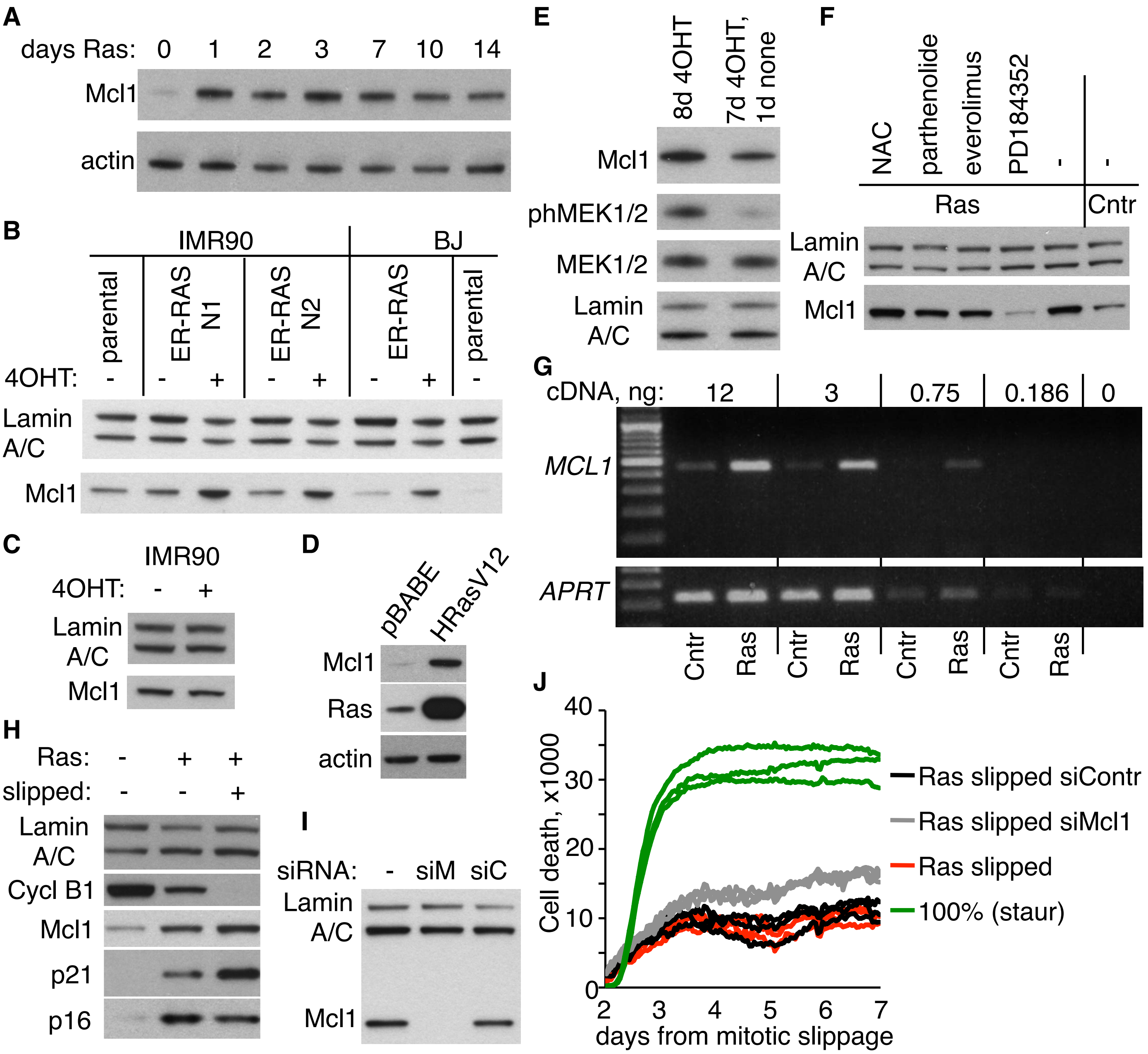
A
days Ras: 0 1 2 3 7 10 14
Mcl1
actin
B
IMR90
BJ
parental
ER-RAS N1
ER-RAS N2
ER-RAS
parental
4OHT: - - + - + - + -
Lamin A/C
Mcl1
C
IMR90
4OHT: - +
Lamin A/C
Mcl1
D
pBABE
HRasV12
Mcl1
Ras
actin
E
8d 4OHT
7d 4OHT, 1d none
Mcl1
phMEK1/2
MEK1/2
Lamin A/C
F
NAC
parthenolide
everolimus
PD184352
-
-
Ras
Cntr
Lamin A/C
Mcl1
G
cDNA, ng: 12 3 0.75 0.186 0
MCL1
APRT
Cntr Ras Cntr Ras Cntr Ras Cntr Ras
H
Ras: - + +
slipped: - - +
Lamin A/C
Cycl B1
Mcl1
p21
p16
I
siRNA: - siM siC
Lamin A/C
Mcl1
J
Cell death, x1000
40
30
20
10
0
2 3 4 5 6 7
days from mitotic slippage
Ras slipped siContr
Ras slipped siMcl1
Ras slipped
100% (staur)

Figure S3

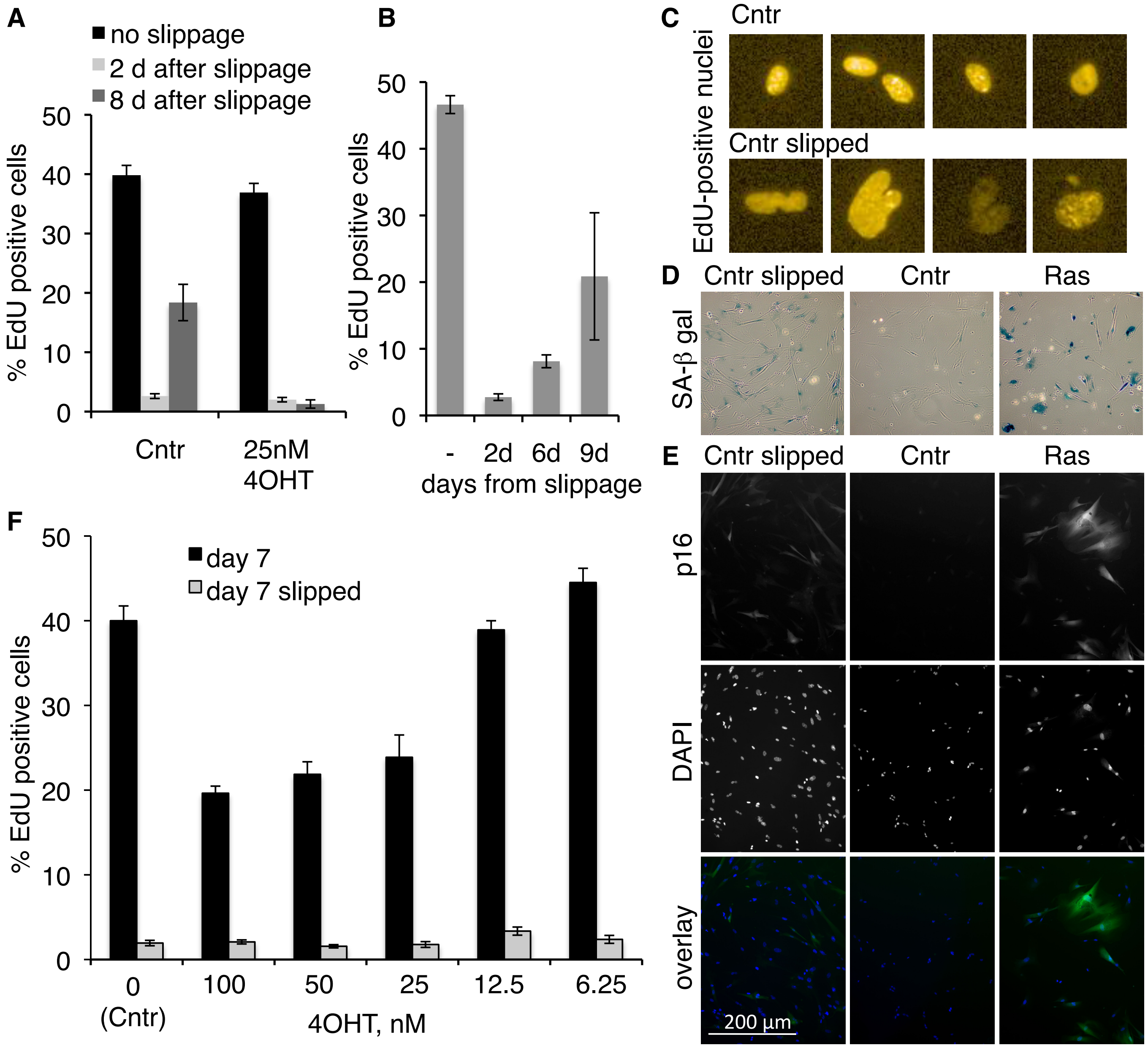
A
no slippage
2 d after slippage
8 d after slippage
% EdU positive cells
0
10
20
30
40
50
Cntr
25nM
4OHT
B
% EdU positive cells
0
10
20
30
40
50
-
2d
6d
9d
days from slippage
C
Cntr
Cntr slipped
EdU-positive nuclei
D
Cntr slipped
Cntr
Ras
SA-β gal
E
Cntr slipped
Cntr
Ras
p16
DAPI
overlay
200 µm
F
day 7
day 7 slipped
% EdU positive cells
0
10
20
30
40
50
0
(Cntr)
100
50
25
12.5
6.25
4OHT, nM

Figure S4

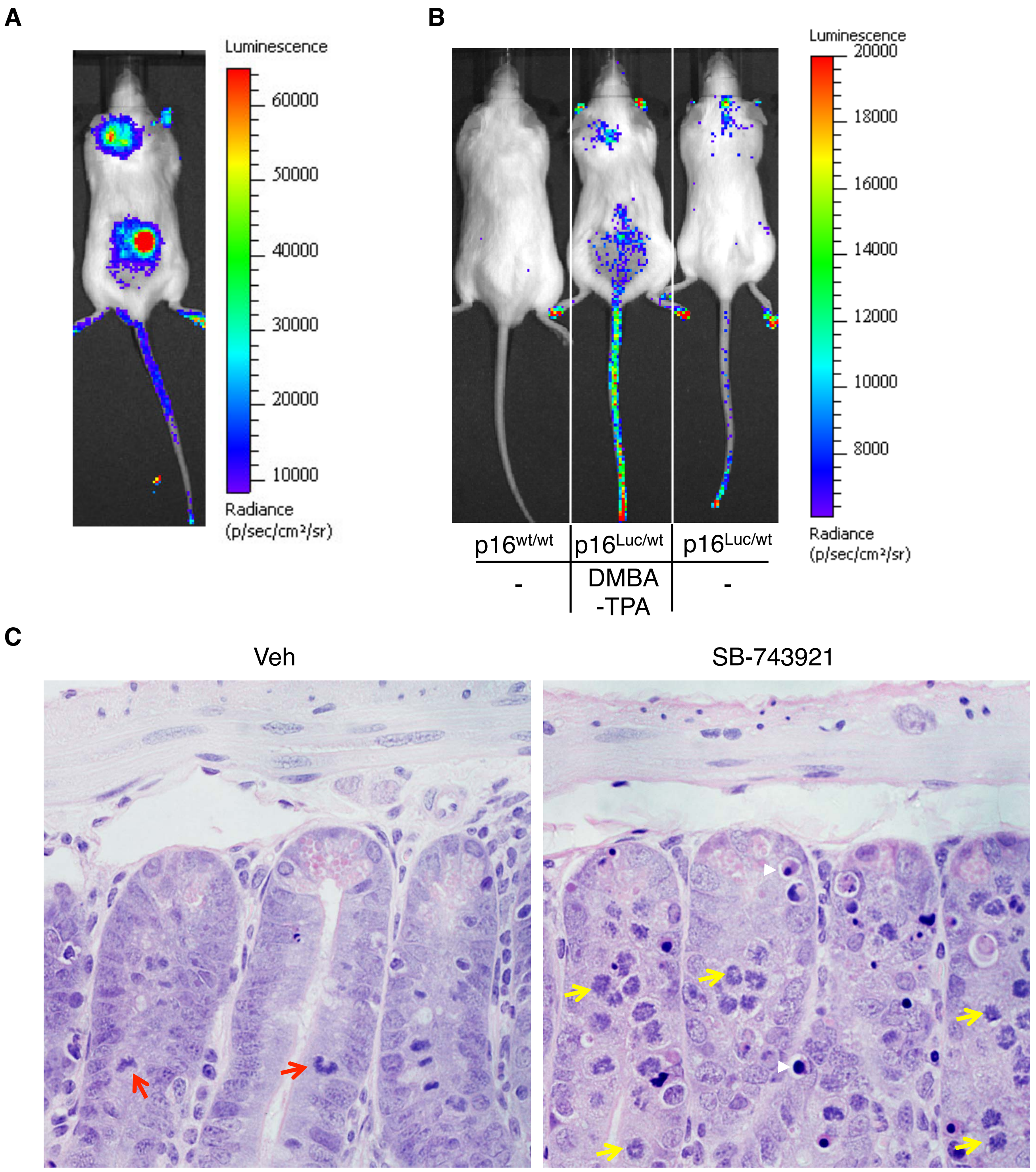
A
Luminescence
60000
50000
40000
30000
20000
10000
Radiance
(p/sec/cm²/sr)
B
Luminescence
20000
18000
16000
14000
12000
10000
8000
Radiance
(p/sec/cm²/sr)
p16wt/wt
p16Luc/wt
p16Luc/wt
-
DMBA
-TPA
-
C
Veh
SB-743921

Figure S5

**Supplemental Figure legends**

**Figure S1**, **Related to Figure 1. Induction of OIS and multinucleation in ERRAS cells. A.** Induction of expression of H-RasV12 and downstream MEK1/2 phosphorylation by tamoxifen (4OHT) in ERRAS cells but not in parental IMR90 cells. Untreated cells or cells treated with 4OHT for 1 day were lysed and blotted for Ras (endogenous and ectopic), MEK1/2 and phospho-Ser270/221 MEK1/2. Lamin A/C is a loading control. **B.** Decline in number of BrdU-incorporating cells upon H-RasV12 activation. ERRAS cells induced for indicated times were pulsed with 10 μM BrdU for 5 hours and stained with anti-BrdU antibodies. Proportion of BrdU-positive cells was calculated from 242-332 cells scored per time point. Aphidicolin-induced S-phase block during BrdU pulse was included as a negative control ("aph"). **C.** SA β-gal staining of control (left) or 14 days induced ERRAS cells (right). **D.** SAHF formation visualised by DAPI staining in 6 days induced ERRAS cells. Size bar 20 μm **E.** Kinetic of multinucleation shown as a percent of ERRAS cells with more than 2 nuclei, scored throughout first 10 days of H-RasV12 activation. Cells were fixed and stained with DAPI and antibodies against Lamin A/C and tubulin. 261-307 cells were scored per each timepoint. **F.** Immunofluorescence of IMR90 cells stably expressing GFP-Lamin A (middle, GFP fluorescence) stained with anti-lamin A/C antibodies (right) and DAPI (left). **G.** No effect of GFP-Lamin A expression on nuclear morphology. Nuclear morphology of untreated IMR90 cells or puromycin-selected IMR90 cells expressing GFP-Lamin A or GFP alone stained with DAPI and Lamin A/C antibody. Percent of cells with normal nucleus (norm), with lobulated nuclei (lobul), with nuclei containing a hole (with hole) or with two or more nuclei (MNC) scored from at least 230 cells for each condition. **H.** Example of GFP-lamin

A expressing ERRAS cells undergoing cell death. Selected frames from time-lapse sequence, with bright field images shown on a top and corresponding GFP signal shown on the bottom. Yellow arrows point to a cell undergoing apoptosis. Note that nuclear GFP-lamin A is detectable for several hours in cell that is morphologically at the late stage of apoptosis. Time stamp, hh:mm. See also Movie S2. **I.** Table summarising the multinucleation events observed in time-lapse images from GFP-Lamin A expressing control ERRAS cells or ERRAS cells induced for indicated times. Approximately 50-200 cells were observed in each time-lapse. *Average ± standard deviation was counted from 6 representative time-lapses for each condition. See also Movies S3-S6. **J.** Plot showing mean (± SD) number of cell divisions per time lapse, as in table H, last row. **K.** Percent of cell divisions (out of total number of dividing cells) that gave rise to MNC via slippage, calculated for indicated time intervals of H-RasV12 activation.

**Figure S2, Related to Figure 4. Effects of DME treatment in control and induced ERRAS cells. A.** DME arrests both 4 days H-RasV12-induced (Ras) and control (Cntr) ERRAS cells with monopolar spindles. Cells were incubated for 18 hours with DME, fixed and stained for microtubules (green), centrosome-associated pericentrin (red) and chromatin (DAPI, blue). Size bar 10 μm. **B.** H-RasV12 activation reduces apoptosis caused by DME treatment. Induced (Ras) or control (Cntr) ERRAS cells were incubated with DME for 17 hours before detecting apoptotic cells with NucView 488 caspase 3 substrate by flow cytometry. Cell debris were excluded from analysis. **C.** As in B, but only phospho-H3-positive cells in the DME-treated population were analysed. **D.** Activated H-RasV12 provides cells with a selective advantage in the presence of DME. IMR90

cells co-expressing inducible H-RasV12 and GFP (red or blue indicate a proportion of induced or control cells respectively) were mixed with equal number of GFP-negative parental cells (proportion indicated by grey). The proportion of GFP-positive cells in the mixed cultures was measured by flow cytometry immediately after mixing and for three consecutive days. In the presence of both 4OHT and DME, H-RasV12 expressing cells exhibited a selective advantage over parental H-RasV12 negative cells (row 1). However, when H-RasV12 was not induced (row 2), DME did not alter the ratio between the two cell types. In the absence of DME, both induced (row 3) and control (row 4) ERRAS cells had a slight growth disadvantage and were eventually outgrown by the parental IMR90 cells. **E.** Treatment of H-RasV12 expressing cells with DME leads to multinucleation. Induced ERRAS cells were treated with DME as in Fig 6A, and two days after drug removal (7 days of H-RasV12 induction) surviving cells were fixed, stained with DAPI and imaged using Operetta scanner. Insert shows a magnified fragment.

**Figure S3, Related to Figures 4 and 6. Upregulation of Mcl1 by activated H-RasV12 contributes to survival of post-slippage cells. A.** Increased Mcl1 level is sustained throughout the 2 weeks of OIS establishment in induced ERRAS cells. Days of 4OHT are shown at the top. Actin used as a loading control. **B.** Two independently derived lines of ERRAS IMR90 cells and one line of ERRAS BJ cells show similar level of Mcl1 upregulation upon 4 days of H-RasV12 activation with 4OHT. Level of Mcl1 in parental IMR90 and BJ cells is provided for comparison. Lamin A/C is used as loading control. **C.** No effect of 7 days of 4OHT on Mcl1 protein level in parental IMR90 cells. **D.** Upregulation of Mcl1 by constitutive H-

RasV12 in IMR90 cells. IMR90 were infected with H-RasV12 or control (pBABE) retrovirus and selected under puromycin for 8 days before cells were lysed and immunoblotted for Mcl1, Ras and actin (loading control). **E.** Increase in Mcl1 level requires continuous H-RasV12 expression. 4OHT was removed for 1 day from induced ERRAS cells at day 7 of induction, and Ras signalling (by phospo-MEK1/2) and Mcl1 level were compared to that in ERRAS cells under 8 days of continuous 4OHT-mediated activation. Lamin A/C is used as a loading control. **F.** Effect of indicated inhibitors on Mcl1 level. Lysates from control (Cntr) or 5 days induced ERRAS cells (Ras, -), or 5 days induced ERRAS cells treated with 10 mM NAC (for 17.5 hours), 20 μM parthenolide (for 17.5 hours), 50 μM Everolimus (for 4 hours) or 5 μg/ml PD184352 (for 4 hours) were separated on SDS-PAGE and immunoblotted for Mcl1 and lamin A/C (as a loading control). **G.** mRNA level of *MCL1* is increased in 4 days induced ERRAS cells, visualized by semi-quantitative PCR. The reactions were performed with decreasing amounts of source cDNAs as indicated. 443 bp band (top) detects *MCL1L* variant. *MCL1S* variant that would be recognized as 195 bp band is below detection limits. *APRT* is used as an internal control. **H.** High level of Mcl1 in slipped cells with activated H-RasV12. Cell lysates from control or 5 days induced ERRAS cells with or without DME-enhanced slippage were blotted for Mcl1 and indicators of cell cycle arrest (cyclin B1, p21 and p16), and lamin A/C as a loading control. **I.** Mcl1 depletion by siRNA in induced post-slipped ERRAS cells. Slipped ERRAS cells at day 6 of H-RasV12 induction / day 1 after slippage (asterisk in Figure 6A) were transfected with Mcl1-targeting (siM) or non-targeting (siC) siRNA or left untreated. Lysates were collected 4 days later and blotted for Mcl1 and Lamin A/C (as a loading control). **J.** Cell death kinetics measured as in Figure 6B in

induced slipped ERRAS cells after transfection with non-targeting (Ras slipped siContr, black) or Mcl1-targeting (Ras slipped siMcl1, grey) siRNA. Data were acquired simultaneously and at the same cell initial densities as data shown in Figure 6B, and identically analysed, allowing a direct comparison between these two datasets. Cell death kinetics of induced slipped ERRAS cells without transfection (Ras slipped, red, the same data as in 6B) and of staurosporine-treated control cells (100% (staur), green, the same data as in 6B) are shown for comparison.

**Figure S4, Related to Figure 7. Mitotic slippage in IMR90 cells does not lead to senescence, but cooperates with H-RasV12 to induce senescence-associated cell cycle arrest.**

**A.** Combination of slippage and subthreshold (for OIS onset) H-RasV12 expression induces effective cell cycle arrest. Percent of replicating cells measured by EdU incorporation at 2 (light grey) or 8 (dark grey) days after slippage (corresponding to 7 or 13 days after induction of H-RasV12) or without slippage (black) in control ERRAS cells (Cntr) or ERRAS cells treated with 25 nM 4OHT. Mean±SD from 7 replicates. **B.** Slippage alone does not induce long-term cell cycle arrest in control ERRAS cells. Percent of replicating cells measured by EdU incorporation at indicated times after DME-induced slippage are shown. Mean±SD from 7 replicates. **C.** Images of EdU-positive nuclei in control ERRAS cells (top) and control ERRAS cells 9 days after slippage (bottom), acquired and presented at the same magnification. Cells were pulsed with EdU for 3 hours before fixation and EdU detection. **D.** SA β-gal staining of control cells 9 days after slippage (left). Untreated uninduced control ERRAS (middle) and senescent

17 days induced ERRAS (right) are given as negative and positive control, respectively. **E.** p16 immunofluorescence in control ERRAS cells 9 days after slippage (left). Overlay colours: DAPI - blue, p16 - green. p16 staining in control (middle) and 14 days-induced (senescent, right) ERRAS cells are shown as negative and positive controls, respectively. Size bar 200 μm. **F.** Combination of mitotic slippage and low-level (sub-threshold for OIS onset) H-RasV12 expression induces effective cell cycle arrest in ERRAS cells (7 days time point). The experiment was conducted as in Fig 7B. Percent of replicating cells measured by EdU incorporation at 2 days after slippage (7 days of induction of H-RasV12) in control ERRAS cells (0, Cntr) or ERRAS cells treated with indicated concentrations of 4OHT. Mean±SD from 7 replicate experiments.

**Figure S5, Related to Figure 7. SB-743921 induces mitotic arrest and activates the p16 promoter *in vivo*. A.** Activation of p16 promoter-driven luciferase expression in p16$^{Luc/wt}$ transgenic mice upon 10 days of wound healing (at the back). The p16 promoter activity was measured by luminescence in the presence of D-licuferin substrate. The second area of luciferase activation (at the neck) is at the site of analgesic injection. The luminescence is shown on the relative scale, colour key to the radiance are provided on the right. **B.** Example of p16 promoter-driven luciferase expression activated by the DMBA-TPA protocol (3$^{rd}$ week, middle) in the p16$^{Luc/wt}$ mouse, as compared to untreated p16$^{Luc/wt}$ littermate (right). DMBA and TPA were applied to the shaved area at the back of the mouse. The area at the neck corresponds to the site of D-luciferin injection. The p16$^{wt/wt}$ mouse imaged in the same way is shown as a control (left). The p16 promoter activity was measured by luminescence in the presence of D-licuferin

substrate. The luminescence is shown on the relative scale, colour key to the radiance are provided on the right. Note that the scale is different from that shown in Fig 7F. **C.** SB-743921 causes mitotic arrest *in vivo*. Images of intestinal crypts from mice that received intraperitoneal injection of vehicle (left panel) or SB-743921 (right panel) for 3 days (Mon-Wed-Fri). H&E staining of paraffin-embedded intestine collected 6 hours after last injection revealed extensive mitotic arrest in SB-743921 but not vehicle treated mice. Red arrows point to the normal mitoses (vehicle-treated), yellow arrows point to representative drug-arrested mitotic cells (SB-743921-treated); white arrowheads point to representative apoptotic cells.

**Supplemental Tables**

**Table S1 (Related to Figure 3). Alignment Statistics**

| Replicate | Sample | Raw Sequence reads | Read Length | Aligned Reads (% of Raw Sequence reads) | Non-Duplicate Reads (% of Aligned Reads) |
|---|---|---|---|---|---|
| 1 | Control | 23,316,203 | 76PE | 18,922,073 (81.15%) | 15,772,985 (83.36%) |
| 2 | Control | 22,254,971 | 76PE | 18,149,692 (81.55%) | 15,038,872 (82.86%) |
| 3 | Control | 28,424,455 | 76PE | 22,917,818 (80.63%) | 18,570,931 (81.03%) |
| 1 | Ras | 26,605,228 | 76PE | 21,768,827 (81.82%) | 17,900,549 (82.23%) |
| 2 | Ras | 19,788,367 | 76PE | 16,210,313 (81.92%) | 13,526,592 (83.44%) |
| 3 | Ras | 22,969,273 | 76PE | 18,874,025 (82.17%) | 15,417,674 (81.69%) |

## Supplemental Table legends

**Table S1, related to Figure 3. RNA-seq alignment statistics.**

Table lists the alignment statistics for each RNA-seq sample. The total reads generated, number (and %) of aligned reads, number (and %) of uniquely aligned reads (as a fraction of aligned reads) is given.

**Table S2, related to Figure 3. RasV12 induced changes in mitotic transcripts.** Table lists genes (first column) involved in mitosis-related process (indicated as "mitosis" in a second column, corresponding to heat map shown in Figure 3D for significantly changed genes in H-RasV12 expressing mitoses), organization and assembly of mitotic spindle (indicated as "Mitotic spindle" in a second column, corresponding to Figure 3F for significantly changed genes in H-RasV12 expressing mitoses), or chromatin maintenance or function (indicated as "Chromatin" in a second column, corresponding to Figure 3G for significantly changed genes in H-RasV12 expressing mitoses). Corresponding mean expression levels for uninduced (Control) and 4 days H-RasV12 activated (Ras) samples (from 3 replicas), log fold change and False Discovery Rate (q-value) are shown.

## Supplemental Movies

**Movie S1, Related to Figure 1. Mitosis.** Typical example of normal mitosis (indicated by arrow) in a GFP-Lamin A-expressing 2 days-induced ERRAS cell. Bright field image (top) and corresponding GFP fluorescence (bottom) are shown in parallel. Time (hh:mm) is indicated at the top. Note the dispersal of nuclear envelope-associated GFP signal as cell enters mitosis (01:00 time point).

**Movie S2, Related to Figure 1 and S1. Cell death.** Typical example of death in a GFP-Lamin A-expressing ERRAS cell. Bright field images (left) and a corresponding GFP fluorescence (right) with cell of interest indicated by an arrow, are shown in parallel. Time (hh:mm) is indicated at the top. Note that nuclear envelope-associated fluorescence persists until after nuclear and cytoplasm compaction and cellular immobilisation (from 0:50 time point onwards) and is only lost simultaneously with the last (terminal) bleb (4:10 time point).

**Movie S3, Related to Figure 1. Cell fusion.** Typical example of cell fusion in induced GFP-Lamin A expressing ERRAS cell. Bright field images (left) and a corresponding GFP fluorescence (right) of 9 days induced ERRAS cells are shown in parallel. Time (hh:mm) is indicated at the top. Note that the two separate cells (indicated by arrows on GFP fluorescence images) fuse at 5:50 time point forming one binucleate cell that spreads (last frame) with two nuclei in close proximity.

**Movie S4, Related to Figure 1. Nuclear fragmentation in interphase.** An example of separation of lobulated nucleus in GFP-Lamin A-expressing 2 days-induced ERRAS cell to two nuclei during interphase. Bright field images (left) and a corresponding GFP fluorescence (right) are shown in parallel. Time (hh:mm) is indicated at the top. Note that nucleus of one of the cells (indicated by arrows in GFP images, a daughter cell generated from mitosis at 2:10) acquires 8-shaped form (21:20 time point), and eventually separates into two (possibly connected)

nuclei within one cell (last frame, right top corner), without intermittent loss of nuclear envelope fluorescence.

**Movie S5, Related to Figure 1. Binucleation.** Typical example of binucleation resulting from cytokinesis failure in induced GFP-Lamin A-expressing ERRAS cell. Bright field images (left) and corresponding GFP fluorescence (right) are shown in parallel. Time (hh:mm) is indicated at the top. Note the cell (indicated by arrow in the GFP time-lapse) that enters mitosis at 1:30 and forms 2 nuclei without cell division at 2:30. Cell spreads at 2:50 as binuclear and remains as such until the end of the time-lapse. While furrowing is not observed in this time-lapse sequence, we cannot exclude a transient furrow formed in the time between image acquisitions.

**Movie S6, Related to Figure 1. Multinucleation.** Typical example of multinucleation after prolonged mitotic arrest and slippage in induced GFP-Lamin A-expressing ERRAS cell. Bright field images (right) and a corresponding GFP fluorescence (left) are shown in parallel. Time (hh:mm) is indicated at the top. Note the cell (indicated by arrow in the GFP time-lapse) that enters mitosis at 01:10 time point and remains rounded for many hours until elongation (from approximately 9:30), constriction of cell body in several places (at 13:00) and its apparent fragmentation (from 15:30). Nuclear envelopes start to reform in several parts of the cell (from 17:30) and the cell spreads as multinucleate (visible from 22:40).

**Movie S7, Related to Figure 1. Survival of multinucleated cell.** An example of Ras-induced GFP-Lamin A expressing cells that remains viable for a long time after multinucleation via mitotic slippage. Bright field images (top) and a corresponding GFP fluorescence (bottom) are shown in parallel. Note the cell that undergoes mitotic slippage at 02:40 and remains alive, motile and multinucleated until the end of the time-lapse (indicated by arrow in the GFP-fluorescent images). Time (hh:mm) is indicated at the top.

.

**Supplemental Experimental Procedures**

*Cell culture and treatment*. IMR90 cells obtained from ATCC were maintained in DMEM supplemented with 20% fetal calf serum, 2 mM L-glutamine and 50 U/ml Pen-Strep, at 3% oxygen level. BJ cells obtained from ATCC were maintained in the same media except 10% fetal calf serum. ERRAS cells were generated by infecting low passage IMR90 or BJ cells with retrovirus carrying pLNC-Ras:ER (Barradas et al., 2009) (encoding ER-HRasV12, kind gift of Jesús Gil, Imperial College, London) that was packaged in Phoenix helper cells (Gary Nolan, Stanford University) as described (Swift et al., 2001) and cultured as above, except in phenol-free DMEM and in the presence of 0.5 mg/ml G418. For H-RasV12 activation, ERRAS cells were treated with 100 ng/ml (unless indicated otherwise) 4-hydroxytamoxifen (4OHT). Mcl1-overexpressing and appropriate control ERRAS cells were generated by infecting ERRAS cells with retrovirus carrying either pLZRS-HA-Mcl1 or pLZRS vector control (both plasmids kind gift of Stephan Tait, University of Glasgow), followed by Zeocin selection. GFP-lamin A expressing cells were generated by retroviral transduction of IMR90 or ERRAS cells with pBABE-puro-GFP-wt-Lamin A ((Scaffidi and Misteli, 2008), Addgene plasmid 17662). Retrovirus infected cells were selected and maintained under 1 μg/ml puromycin. GFP-expressing cells were generated by retroviral transduction of pQCXIP-GFP (Ye et al., 2007), followed by puromycin selection as above. For constitutive RasV12 expression, IMR90 cells were retrovirally transduced with pBabe-puro-H-RasV12 or control pBabe-puro (both plasmids kind gift of William Hahn, Dana Farber Cancer Institute, Addgene palsmid N9051) and selected with puromycin as above.

Staurosporine (Sigma), Everolimus (LC Laboratories), parthenolide (Sigma), PD184352 (Tocris Bioscience) were added to full growth media at indicated concentration for indicated time. N-acetylcysteine (NAC, Sigma) was dissolved in full media up to final concentration 10 μg/ml and filtered after adjusting pH to 7.2. For mitotic arrest, cells were treated with Eg5 inhibitor III (DME, Dimethylenastron, Calbiochem/Merck Cat.N. 324622) at 1 μM final concentration for indicated times. For harvesting mitoses, unattached cells arrested with DME for several hours were collected with 8-10 rounds of vigorous but careful pipetting in drug-containing growth media. Mitotic index was determined by visual scoring of cells with mitotically compacted chromosomes after 10 min incubation with PBS supplemented with small amounts of Hoechst DNA stain and Propidium Iodide, followed by fixation in 4% Paraformaldehyde in PBS. For harvesting cells slipped out of mitosis, collected mitoses were further incubated in full media containing DME for up to 3 days and any unattached cells and cell debris were thoroughly washed away before attached (slipped) cells were harvested by trypsinisation.

*Apoptosis assay.* Cells were incubated with media containing 5 μM NucView 488 Caspase 3 substrate (Biotium) for 40 min. Cells were collected, fixed in PBS containing 2% paraformaldehyde for 15 min and permeabilised with 0.1% triton X100 in PBS. After co-staining with required antibodies (e.g. anti-phospho-Histone H3 (Ser10), Millipore), the cellular level of activated caspase 3 substrate was measured by flow cytometry using BD FACSCalibur (BD Biosciences) and analysed using FlowJo software (Tree star).

*siRNA.* Cells were transfected with 50 nM Mcl1-targeting siRNA (siGENOME SMARTpool, M-004501-08-0005) or non-targeting siRNA (siGENOME Pool N2, D-001206-14-05, both from Dharmacon/Thermo Scientific) using Oligofectamine reagent (Invitrogen). For time-lapse imaging or cell death kinetics, cells seeded onto 24 well plates were transfected with 30 nM siRNA using 0.9 µl Lipofectamine 2000 (Invitrogen) per well 1-2 days before imaging.

*Immunofluorescence.* Cells cultured on glass coverslips were fixed with 4% paraformaldehyde in PBS or PHEM (60 mM PIPES, 4 mM $MgSO_4$, 25 mM HEPES, 10 mM K-EGTA, pH=6.9) and permeabilised with 0.1-0.5% triton X100 (for pericentrin, phospho H3 or p16 detection). Alternatively, cells were treated with 100% ice-cold methanol (for microtubules and lamin A/C detection) or 2.5% glutaraldehyde followed by 0.1% NaBrH4 in PBS (for visualisation of anaphase bridges). Immunostaining was performed in PBS containing 2% BSA, 0.1% triton X100 and sodium azide with following antibodies: anti-lamin A/C (Cell Signaling 2032S, 1:200), anti-alpha tubulin (DM1A, Sigma T6199, 1:1000), anti-pericentrin (AbCam ab4448, 1:500), anti-p16 (JC8, Santa Cruz, sc-56330, 1:100), and anti-phospho histone H3 (Ser10) (Millipore 06-570, 1:1000). Fluorescently-labelled secondary antibodies (Invitrogen) were used at 1:400 dilution. Cells were counterstained with 0.2 µg/ml DAPI (Sigma) in PBS and mounted with Prolong Gold mounting media (Invitogen).

*Imaging and file processing*

Immunofluorescently stained fixed cells were imaged either with epi-fluorescent Nikon Eclipse 80i microscope equipped with Hamamatsu ORCA-ER camera and

operated by MetaMorph software (Molecular Devices), or with Zeiss 710 (Axioimager) confocal microscope with 7 laser lines and Zeiss Quazar detectors, operated by ZEN software. B-gal and IHC sections were imaged using Olympus BX51 microscope equipped with Olympus DP70 digital camera and Cell*D imaging system (Olympus). Time-lapse microscopy was performed with Nikon Eclipse TE2000-E microscope equipped with Ocolab environmental chamber that maintains temperature, $CO_2$ and moisture. Images were acquired using CoolSNAP camera (Photometrics) operated by MetaMorph, at 10-30 min time intervals, as indicated. Routinely, 4 non-overlapping time-lapse sequences were acquired within each well of a multiwell plate with cells. For fluorescent time-lapse imaging, glass-bottom multiwell plates were used. Images acquired in MetaMorph were further processed in Image J that included linear color adjustment (identical between control and experimental samples, when used for comparison of signal intensity), cropping and combining single channels into a multi-colored or stacked image. For time-lapse microscopy, selected image sequences were assembled into movies (.avi file format) within Image J, converted into .m4v file format using HandBrake software and further converted into .mov file format in QuickTime player 7. Confocal images were processed using ZEN software, exported and further adjusted in Photoshop or Image J in a linear fashion.

*Measuring duration of mitosis/mitotic arrest and frequency of mitotic slippage*

Mitotically arrested cells in time-lapse images of DME-treated populations or untreated mitoses were tracked using Image J. The duration of mitotic arrest was calculated from the time of cell rounding (with signs of chromatin condensation

when visible) to either cell death (at the onset of extensive terminal blebbing) or slippage (cell flattening). For each experimental point, we scored all identifiable mitotic cells in 3-4 time-lapse sequences from the same well. The data were further analysed in Microscoft Excel.

*Immunoblotting.* Adherent cells were washed twice with PBS and boiled in 2xSDS lysis buffer containing 4% SDS, 12.5 mM TrisHCl pH 6.8 and 20% glycerol. Subsequently DTT was added up to 200 mM final concentration. Alternatively, cells were lysed in cold MEBC buffer containing 0.5% NP40, 50 mM Tris HCl, pH7.5, 100 mM NaCl, 5 mM EDTA, 5 mM K-EGTA. For detection of phospho-epitopes and/or unstable proteins, lysis buffer was supplemented with phosphatase and protease inhibitors (1 mM sodium orthovanadate, 50 ng/ml PMSF, protease inhibitor cocktail (Sigma P8340) used at 1:100 and phosphatase inhibitor cocktail (Sigma, P0044) used at 1:1000). Lysates were separated on 10% or 4-12% SDS-PAGE and transferred onto Whatman Protran BA85 nitrocellulose membrane (Sigma Aldrich). Total protein and protein markers were detected with reversible Ponceau S staining (Sigma, 81462) and immunoblotted in blocking solution containing 5% non-fat milk and 0.1% Tween 20 in PBS with following primary antibodies: anti-Ras (BD Transduction laboratories 610001, 1:500) anti-MEK1/2 (Cell Signaling/NEB 9122, 1:2000), anti-phospho Ser217/221 MEK1/2 (Cell signaling/NEB 9121, 1:1000), anti-p44/p42 MAPK (ERK1/2) (Cell Signaling 9102, 1:2500), anti-phospho Thr202/Tyr204 p44/p42 MAPK (ERK1/2) (Cell signaling/NEB 9101, 1:1000), anti-lamin A/C (Cell Signaling 2032S, 1:1000-1:2000), anti-XIAP (Cell Signaling 2045, 1:1000), anti-caspase 9 (Cell Signaling 9502S, 1:1000), anti-Mcl1 (BD

Pharmingen 559027 or Cell Signaling/NEB 9941, 1:1000 or 1:2000), anti-actin (Sigma 1978, 1:400000), anti-Bax, anti-Bid, anti-Bak (Cell Signaling/NEB 9942, all 1:1000); anti-Bcl2, anti-Bcl-xL (Cell Signaling/NEB 9941, all 1:1000), anti-cyclin B1 V152 (Santa Cruz sc-53236, 1:1000), anti-p21 (Santa Cruz sc-817, 1:1000), anti-p53 (DO-1, Santa Cruz sc-126, 1:1000) and anti-p16 (BD Pharmingen, 51-1325GR, 1:500). HRP-labeled secondary antibodies (Cell Signaling) were used at 1:5000 dilution in blocking solution, and detected with ECL kit (Pierce). For quantitative detection, IRDye 680 or 800 labeled secondary antibodies (Li-COR Biosciences) were used at dilution 1:5000 and detected with LiCOR scanner.

*Protein stability measurements.* Control or 1 day induced ERRAS cells were treated in triplicates with 10 μg/ml cycloheximide (Sigma) for indicated time and lysed in 2xSDS lysis buffer with protease and phosphatase inhibitors (see *Immunoblotting*). 5 μg lysates were separated on 10% PAGE, immunoblotted simultaneously for Mcl1 and actin using fluorescent secondary antibodies, scanned and quantified using LiCOR imaging system. Relative level of Mcl1 was calculated after normalization to actin signal intensity in the same sample. Mcl1 half-life value ($t_{1/2}$) was calculated from initial 80 min of exponential decay, as follows:

$$t_{1/2}=\ln(2)/k;\ \ k=(\ln(A_t)-\ln(A_{t+\Delta t}))/\Delta t,$$

where $A_t$ – relative Mcl1 amount at time t, and $\Delta t$ – time interval between two measurements.

*Measurement of DNA synthesis.* Cells seeded onto glass coverslips were pulsed for 5 hours with 10 µm BrdU, fixed with 4% PFA in PBS and permeabilised with 0.5% TritonX100 in PBS before DNase I treatment and immunostaining with anti-BrdU antibodies (DAKO). When indicated, 10 µM aphidicolin was added 1 hour before and during BrdU treatment. Cells were counterstained with DAPI and examined using Nikon Eclipse 80i microscope equipped with Hamamatsu ORCA-ER camera, operated by MetaMorph software (Molecular Devices). BrdU-positive and BrdU-negative nuclei were scored visually. Alternatively, cells seeded onto black-walled 96 well CellBIND plates (Corning) were pulsed with 10 µM EdU for 3 hours before fixation and EdU detection using Click-IT® EdU imaging kit (Life Technologies) according to the manufacturer instructions, and staining with DAPI. Plates were scanned with Operetta High Content Imaging system (PerkinElmer) at 10x magnification, with 11 images taken in each well, and percent of EdU positive cells per well was determined automatically using Harmony software. When required (eg for Fig S2E and Fig S4C), individual images were exported. We routinely seeded 8 wells per sample, of which one well was not pulsed with EdU and served as negative control.

*PCR and qPCR.*

Primers:

| assay | target | sequence |
|---|---|---|
| PCR | *MCL1L*, *MCL1S* (Gao and Koide, 2013) | 5’- ATCTCTCGGTACCTTCGGGAGC -3’<br>5’- CCTGATGCCACCTTCTAGGTCC -3’ |
| PCR | *APRT* | 5’-TGGAGATTCAGAAAGACGCCC-3’ |

| | | |
|---|---|---|
| | | 5'-GCCCTGTGGTCACTCATACTGC-3' |
| qPCR | *MCL1L* | 5'- TAAGGACAAAACGGGACTGG -3'<br><br>5'- ACATTCCTGATGCCACCTTC -3' |
| qPCR | *GAPDH* | 5'- GAGAGACCCTCACTGCTG -3'<br><br>5'- GATGGTACATGACAAGGTGC -3' |

Total RNA was extracted using RNeasy plus kit (Qiagen), followed by DNase I treatment. cDNA was produced using oligo-dT primers. Semi-quantitative PCR was performed with indicated amount of cDNA, using Recombinant Taq DNA polymerase kit (Life Technologies, 10342020) as per manufacturer instructions with following parameters: 5 min at $95^0$C, 30 cycles with 30 sec $94^0$C, 30 sec $58^0$C and 50 sec $72^0$C each, with final elongation step for 7 min at $72^0$C before cooling down to $4^0$C. Products were electrophoretically separated in 2% agarose gel containing ethidium bromide and visualized using GeneGenuis gel imaging system (Syngene). Real-time PCR was performed using separately designed primers with the SYBR®-Green master mix (Life Technologies) on the BioRad Chromo4 thermo cycler. The qPCR products were validated both electrophoretically and by examining melting curves. Each reaction was done in triplicates, and obtained C(t) data for *MCL1* species and a housekeeping gene (*GAPDH*) were analysed using REST program to calculate changes in gene expression.

*Mice*

For generation of mouse pancreatic intraepithelial neoplasias (mPanINs) we used male and female progenies of mice heterozygous for *Lox-Stop-Lox-KRas*$^{G12D}$

transgene targeted to the endogenous Ras locus, crossed with pancreas-specific *Pdx1*-Cre mice. Resulting PDX1Cre$^{+/0}$::LSL-KRas$^{G12D/wt}$ mice on mixed C57Bl6 background (Hingorani et al., 2003) spontaneously developed pre-malignant PanINs (senescent lesions) that eventually give rise to metastatic adenocarcinomas (Hingorani et al., 2003; Morton et al., 2010). PDX1Cre$^{+/0}$::LSL-KRas$^{wt/wt}$ mice were used as a control.

For measuring activity of p16 promoter *in vivo*, we used males and females heterozygous Albino C57Bl6 luciferase reporter mice, in which gene body of endogenous p16$^{INK4a}$ was replaced by a luciferase gene (Burd et al., 2013). Ras mutations were induced by ectopic application of 150 µl of 166 µg/ml 7,12-Dimethylbenz(a)anthracene (DMBA, Sigma Aldrich) in acetone to shaved dorsal skin of 7-9 week old mice, followed by 1 week rest before triweekly ectopic applications of 150 µl of 20.83 µg/ml 12-O-Tetradecanoylphorbol-13-Acetate (TPA, LC laboratories) in acetone, the protocol used for initiation of cutaneous two-stage carcinogenesis (Filler et al., 2007). Mice received the total of three 100 µl intaperitoneal injections of 0.5 mg/ml SB-743921 (Selleckchem) in 2% Cremophor, 98% sterile water pH 5.0 (final dose 2.5 mg/kg per injection) or equal volume of vehicle during the first week of TPA treatment, 6 hours after TPA application each.

In a pilot study, 7 weeks old FVB or C57Bl6 female mice received 2 weekly courses of 3 x 100 µl intaperitoneal injections of 0.5 mg/ml SB-743921 (Selleckchem) in 2% Cremophor, 98% sterile water pH 5.0 (final dose 2.5 mg/kg per injection) or equal volume of vehicle, and their health was monitored for another two weeks. Two mice per strain/treatment were sacrificed 6 hours after third injection, and the effectiveness of drugs was assessed by quantifying

mitotic and apoptotic indices in Haematoxylin & Eosin stained paraffin sections of intestinal crypts.

As a positive control for p16 activation, we used p16$^{Luc/wt}$ mouse 10-17 days after cutaneous wounding. The 5 mm diameter wound was created by dorsal skin incision of an anesthetized mouse. Subcutaneous injection of rimadyl prior the biopsy and oral rimadyl for 3 days after the biopsy was used for analgesia.

All mice were maintained in pathogen-free facilities proactive in environmental enrichment. All animal work was carried out according to UK Home Office regulations, in line with the EU directive 2010 and approved by ethical review (University of Glasgow).

*In vivo luciferase detection.*

Mice subcutaneously injected with 100 µl of 30 mg/ml RediJect D-Luciferin Bioluminescent Substrate (Perkin Elmer) were anesthetized with isoflurane and, 5 min after luciferin injection, imaged using IVIS Spectrum system (Caliper Life Sciences, Perkin Elmer). The radiance within equally sized dorsal regions (the areas subjected to the DMBA and TPA applications) was calculated using Living Image software (Caliper Life Sciences), before subtracting the background, derived by identical measurements of similarly treated p16$^{wt/wt}$ Albino C57Bl6 mouse (without a transgene).

*Histopathology, immunohistochemistry and tissue immunofluorescence*

4 µm sections cut from formalin fixed paraffin embedded blocks were baked onto poly-lysine slides for 60 minutes at 60°C. Sections were stained with

Haematoxylin & Eosin for morphological assessment or processed for immunohistochemistry (IHC) or immunofluorescence (IF) with the following antibodies: Melan A (Dako, M7196), phosho-ERK1/2 (Cell Signalling, 9101), Mcl1 (Proteintech 16225-1-AP). All sections were dewaxed in xylene, rehydrated through graded ethanols and washed in deionised water before heat-induced epitope retrieval using a Dako Pre-treatment module, either by heating for 25 minutes at $98^0$C in 10mM Sodium Citrate pH6 retrieval buffer (Thermo, TA-250-PM1X) (for pERK1/2 IHC and for IF) or in 1mM Tris-EDTA pH9 retrieval buffer (TA-250-PM4X) (for Melan A); alternatively, antigen retrieval was performed in a microwave-heated pressure cooker for 5 minutes in citrate buffer pH6 (Dako) (for Mcl1).

Endogenous biotin and peroxidase activity were quenched using Dako's peroxidase block (S2023), and sections were further blocked in 10% normal goat serum (Dako) in antibody diluent (Dako) (for Mcl1) or in 5% normal goat serum (for IF). Primary antibodies applied for 45 minutes at room temperature or at $4^0$C overnight (for Mcl1) were followed by appropriate secondary antibody: for pERK1/2 and Mcl1, Rabbit EnVision (Dako, K4003), for Melan A, Mouse EnVision (Dako, K4001). IHC sections were washed in TBST before application of Dako Liquid DAB (K4011) for 10 minutes to allow visualisation of the antigen. The reaction was terminated in deionised water before counterstaining the nuclei using Gills Haematoxylin. The sections were rinsed in water, dehydrated through graded ethanol, taken through xylene and then mounted using DPX mounting media (CellPath). The IF sections were stained in DAPI (Sigma) diluted 1:1000 in PBS. IHC staining was carried out on a Dako Autostainer Link48.

*Statistical analysis*

P-values were obtained using paired or unpaired two-tailed T-test, chi-square or two-way ANOVA, using PRISM or Microsoft Excel software.

*RNA sequence and data analysis*

RNA was isolated from cell pellets using RNAeasy kit (Qiagen), and DNA was removed using RNase-free DNase set (Qiagen). Quality of RNA was assessed on Agilent 2100 Bioanalyser using Agilent RNA 6000 Nano Kit (Agilen Technologis). To prepare the oligo-dT - based library, we used Illumina TruSeq RNA Sample Prep Kit, v2.0. The library was sequenced on a NextSeq500, using a NextSeq500 Mid Output kit 150 cycles flowcell. Reads were trimmed using Trim Galore (v0.3.0) (http://www.bioinformatics.babraham.ac.uk/projects/trim_galore/) and quality assessed using FastQC (v0.10.0) (http://www.bioinformatics.bbsrc.ac.uk/projects/*fastqc/*). Paired-end reads were aligned to the human genome (hg19) using a splicing-aware aligner (tophat2) (Kim et al., 2013). Duplicate reads were identified using the Picard tools (1.98) script mark duplicates (http://picard.sourceforge.net.). Reference splice junctions were provided by a reference transcriptome (Ensembl build 73), and novel splicing junctions were determined by detecting reads that span exons that are not in the reference annotation. Aligned reads were processed to assemble transcript isoforms, and abundance was estimated using the maximum likelihood estimate function (cuffdiff) from which differential expression and splicing can be derived (Trapnell et al., 2013). Genes of significantly changing expression were defined as FDR corrected p-value <0.05. For generation of RNA-

seq heatmaps, the FPKM value was calculated for each gene based on aligned reads, using Cufflinks (Trapnell et al., 2013). Z-Scores were generated from FPKMs. Hierarchical clustering was performed using the R library heatmap.2 and the distfun='pearson' and hclustfun='average'. Principal component analysis (PCA) was performed using the FPKM values of all ensembl 73 genes of status 'known' and biotype 'coding'. For ontological analysis, differentially expressed genes were analysed using the DAVID (Huang da et al., 2009) tool Functional Annotation Chart, with the default human background. To calculate the significance of gene set enrichment, empirical p-values were generated using the USeq (v7.1.2) tool IntersectLists (Nix et al., 2008). The -t value used was 22,008, as the total number of genes of status 'known' and biotype 'coding' in ensembl genes 73. The number of iterations used was 10,000.

## Supplemental References


Cruickshanks, H.A., McBryan, T., Nelson, D.M., Vanderkraats, N.D., Shah, P.P., van Tuyn, J., Singh Rai, T., Brock, C., Donahue, G., Dunican, D.S., *et al.* (2013). Senescent cells harbour features of the cancer epigenome. Nat Cell Biol *15*, 1495-1506.

Filler, R.B., Roberts, S.J., and Girardi, M. (2007). Cutaneous two-stage chemical carcinogenesis. CSH Protoc *2007*, pdb prot4837.

Gao, Y., and Koide, K. (2013). Chemical perturbation of Mcl-1 pre-mRNA splicing to induce apoptosis in cancer cells. ACS Chem Biol *8*, 895-900.

Gao, Y., and Koide, K. (2013). Chemical perturbation of Mcl-1 pre-mRNA splicing to induce apoptosis in cancer cells. ACS Chem Biol *8*, 895-900.

Huang da, W., Sherman, B.T., and Lempicki, R.A. (2009). Systematic and integrative analysis of large gene lists using DAVID bioinformatics resources. Nat Protoc *4*, 44-57.

Kim, D., Pertea, G., Trapnell, C., Pimentel, H., Kelley, R., and Salzberg, S.L. (2013). TopHat2: accurate alignment of transcriptomes in the presence of insertions, deletions and gene fusions. Genome Biol *14*, R36.

Nix, D.A., Courdy, S.J., and Boucher, K.M. (2008). Empirical methods for controlling false positives and estimating confidence in ChIP-Seq peaks. BMC Bioinformatics *9*, 523.

Scaffidi, P., and Misteli, T. (2008). Lamin A-dependent misregulation of adult stem cells associated with accelerated ageing. Nat Cell Biol *10*, 452-459.

Swift, S., Lorens, J., Achacoso, P., and Nolan, G.P. (2001). Rapid production of retroviruses for efficient gene delivery to mammalian cells using 293T cell-based systems. Curr Protoc Immunol *Chapter 10*, Unit 10 17C.

Trapnell, C., Hendrickson, D.G., Sauvageau, M., Goff, L., Rinn, J.L., and Pachter, L. (2013). Differential analysis of gene regulation at transcript resolution with RNA-seq. Nat Biotechnol *31*, 46-53.

Ye, X., Zerlanko, B., Kennedy, A., Banumathy, G., Zhang, R., and Adams, P.D. (2007). Downregulation of Wnt signaling is a trigger for formation of facultative heterochromatin and onset of cell senescence in primary human cells. Mol Cell *27*, 183-196.
